# Controlled Dimerization of Rhodium(I) Isocyanides Enables Photophysical Properties Beyond Mononuclear Complexes

**DOI:** 10.1021/jacsau.6c00461

**Published:** 2026-05-20

**Authors:** Alexander J. Bukvic, Leander Spierling, Daniel Häussinger, Oliver S. Wenger

**Affiliations:** Department of Chemistry, 27209 University of Basel, St. Johanns-Ring 19, 4056 Basel, Switzerland

**Keywords:** pincer-ligands, self-assembly, aggregation, metal−metal interactions, luminescence

## Abstract

Recent developments in photoactive transition metal complexes have largely centered on mononuclear systems. Polynuclear architectures that are capable of metal–metal interactions and cooperative effects, such as enhanced luminescence or multielectron photoreactivity, have received less attention, however. A key challenge in advancing such systems lies in achieving structural precision to prevent the formation of complex mixtures, including ill-defined oligomers of variable lengths. Here, we report the controlled assembly of discrete dimers composed of stacked square-planar complexes, notably without the use of bridging ligands, but also with them. Using new tridentate pincer-type isocyanide ligands with varying backbones and coordination bite angles, we obtain rhodium­(I) complexes that can be further modulated at the fourth coordination site. Pincer bite angle, the auxiliary ligand at the fourth coordination site, and solvent polarity control the distinct aggregation behavior of the complexes. The resulting dimers exhibit metal–metal interactions that produce near-infrared fluorescence and substantially longer triplet excited-state lifetimes than the nonemissive monomers. This work demonstrates how molecular design and synthetic control over cooperative interactions between individual metal complexes can give rise to emergent photophysical properties. It establishes design principles for precise supramolecular assembly of photoactive coordination units, highlighting new opportunities for photonic applications beyond mononuclear systems.

## Graphical abstract

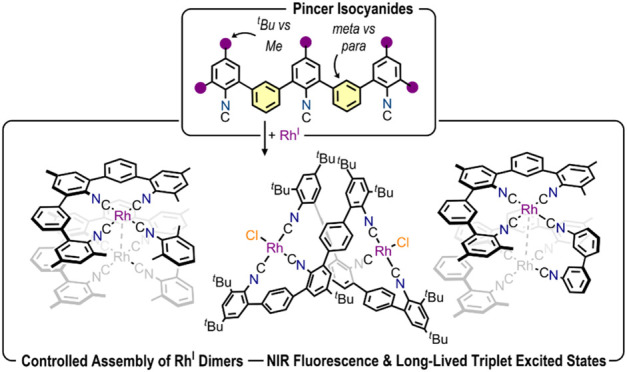

## 1 Introduction

Harnessing cooperative effects between individual metal complexes offers fundamentally new opportunities for photochemistry beyond current limitations.^1–5^ The prospect of photoinduced multielectron transfer, as opposed to conventional single-electron transfer reactivity, holds significant promise for applications in artificial photosynthesis and photoredox catalysis.^6–9^ In photophysical contexts, metal–metal cooperativity can be exploited to improve luminescence quantum yields and photostability through increased excited-state delocalization.^10^ In addition, such cooperativity offers opportunities to control intersystem crossing and, in combination with excited-state delocalization, can enable near-infrared phosphorescence relevant to (bio)­imaging applications.^11,12^

One approach to developing polynuclear complexes with emergent photophysical and photochemical properties is the assembly of individual transition metal complexes into structurally well-defined supramolecular architectures that incorporate metal–metal interactions.^13–16^ Control upon such assemblies enables detailed optical spectroscopic studies, gaining fundamental insights into structure–property relationships.^17^ Ligand design plays a central role in achieving this level of control, while additional parameters such as concentration, solvent, and temperature can guide the formation of well-defined polynuclear aggregates.^18,19^

Under appropriate conditions, square-planar d^8^-metal complexes can aggregate into one-dimensional stacks where the aggregation of individual monomers into oligomeric chains is achieved (Scheme 1a).^2^ The formed supramolecular assemblies feature noncovalent metal–metal interactions, resulting in distinctive luminescent properties. Interactions between filled d_*z* ^2^_ orbitals establish a new set of fully occupied dσ bonding and dσ* antibonding orbitals.^20,21^ These orbitals jointly possess net bonding character and, together with dispersion interactions, provide the thermodynamic driving force for aggregation, effectively overcoming Coulombic repulsion between cationic monomers.^2^

**Scheme 1:**
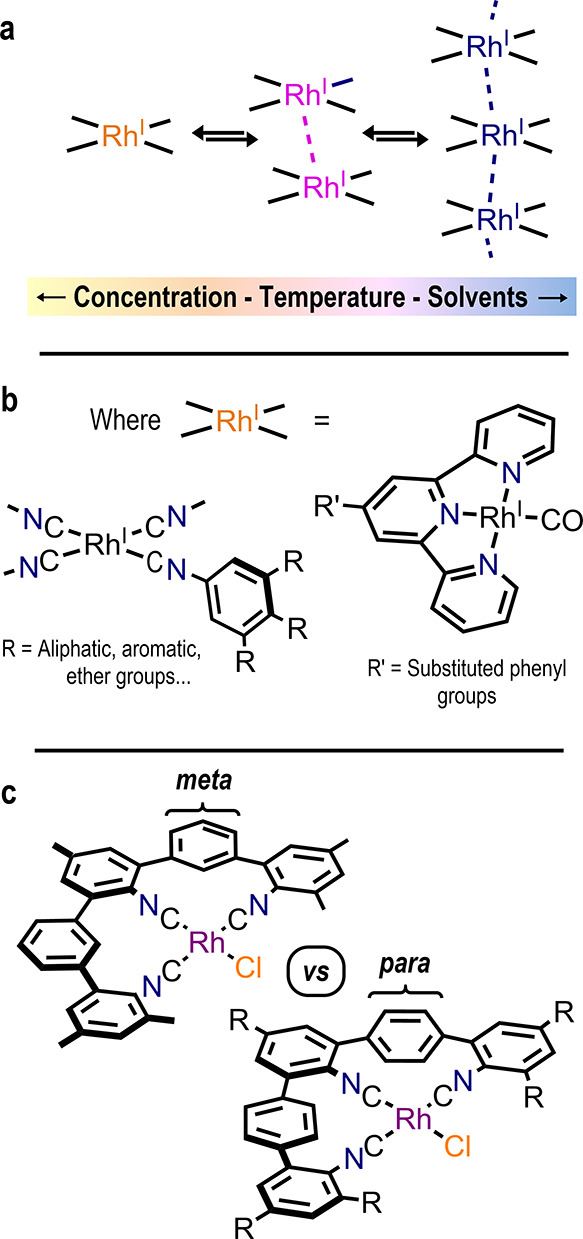
(a) Overview of the Speciation Equilibrium of Rhodium­(I) Complexes Showing Monomers, Dimers, and Oligomers, as Influenced by Concentration, Temperature and Solvent. (b) Selected Examples of Monomers That Exhibit Stacking Behavior.^23,35^ (c) Pincer-Type Ligands with Different Bite Angles and R-Groups (R = Me or ^*t*^Bu) Used for Rhodium­(I) Complexes in This Work. Chloride at the Fourth Coordination Site is Further Replaceable

From a photophysical and photochemical perspective, it is especially relevant that the new dσ* antibonding orbitals become the highest occupied molecular orbitals (HOMOs). The lowest-unoccupied molecular orbitals (LUMOs) are generally either ligand-based π* orbitals or pσ orbitals resulting from the overlap of unoccupied metal-centered p_*z*_ orbitals.^2^ As a result, the lowest-energy excitations that determine the photophysical and photochemical behavior of these systems correspond to either metal–metal-to-ligand charge transfer (MMLCT) excited states or,^22^ in the case of Rh^I^, mixing with 4dσ*→ 5pσ excitations to overall form transitions with mixed excited-state character.^23,24^ As a property emerging from metal–metal interactions across individual monomers, these new absorption bands directly correlate with the extent of stacking and typically cause new absorptions in the visible to near-infrared regions.^18,19^ Ligand based intermolecular forces between monomers, such as π–π stacking or dispersive forces, can provide additional stabilization within the aggregates that also serve as a means of directing supramolecular aggregation. Complexes of Pt^II^,^18,25–28^ Au^III^,^29,30^ Rh^I^,^19,31–36^ and mixed-metal systems^14,37^ are among the most studied in this context.

Achieving precise control over defined architectures and their equilibria remains a challenge due to the intrinsic sensitivity and dynamic nature of many of these systems.^19,38^ An alternative strategy is to utilize tailor-made, bridging ligands to force the aggregation process. This has provided valuable insights into metal–metal interactions and the resulting unique photophysical and photochemical behaviors in several Pt^II^,^5,20,22,39–41^ Rh^I^,^42–47^ Ir^I^,^48^ and Ni^II^ dimers.^13^ Fine-tuning of metal–metal distances through ligand design offers a means to modulate the relevant electronic excitations. This is often not straightforward with bridging ligands, however, due to inherent structural constraints. An alternative approach would be to control the assembly into discrete dimers (or well-defined higher oligomers) without the need for bridging ligands.^49^

We aimed to explore ligand design strategies that enable a higher level of control toward the formation of discrete stacks from simple monomers without relying on bridging ligands. We chose Rh^I^ isocyanide complexes to explore this, as to expand upon the prominent class of compounds known to form aggregates,^5,23,35^ while introducing chelating aryl isocyanides as a novel structural feature. This is in contrast to the many prior studies that employed only monodentate isocyanide ligands.^23,32,35,50^ Rh^I^ is a suitable metal center for exploiting metal–metal interactions due to its spatially extended 4d and 5p orbitals, compared with the more compact 3d and 4p orbitals of more abundant first-row transition metals.^51^

Isocyanide ligands are valued for their strong σ-donor and π-acceptor properties, which match the electronic demands of electron-rich Rh^I^ and disfavor oxidation, affording air- and moisture-stable complexes.^52,53^ The phenyl units of aryl isocyanides and auxiliary modifications can promote secondary stabilization through π–π stacking and solvophobic effects, alongside metal–metal interactions. Applications of tetrakis­(isocyanide)­rhodium­(I) aggregates range from nanowire formation,^54,55^ within material sciences^33,56,57^ and toward bioimaging tools.^11,12,50^

Expanding away from monodentate isocyanide ligands,^58^ the potential of chelating ligands has been demonstrated with multidentate phosphines,^44,45^ nitrogen-donors (Scheme 1b),^35,59^ and carbenes.^1,35,60^ These promote favorable stacking and enhanced stability in d^8^-metal complexes. Tridentate, pincer-type ligands further offer the potential to form highly stable complexes with strong resistance to dissociation.^61,62^ Their rigid, typically planar geometry, is also advantageous toward complex stacking,^59^ and are overall well-suited for self-assembly processes. Combining this structural pincer motif with isocyanide donors represents a promising yet unexplored strategy for designing effective supramolecular assemblies. Although pincer–isocyanide ligands are known,^63,64^ they have not been studied in the context of self-assembly.^42^ Overall, this strategy builds on our recent study of a macrocyclic, four-coordinate isocyanide ligand, which allowed for partial control of the aggregation of corresponding Rh^I^ complexes using solvent as the primary control factor.^36^

Herein, we report a new family of tridentate, pincer-type arylisocyanide ligands, with systematic tuning of bite angles and peripheral substituents to control intermolecular stacking (Scheme 1c). The resulting supramolecular aggregation of the corresponding Rh^I^ complexes was highly controlled through isocyanide ligand, solvent choice and variations at the fourth coordination site. Under appropriate conditions, we can direct the formation of three distinct, well-defined dimeric Rh^I^ systems while avoiding the commonly observed formation of ill-defined mixtures of dimers and higher oligomers. This, in turn, allows the study of dimer luminescence and excited states properties and facilitates the establishment of structure–property correlations that are usually difficult to achieve without precise control over dimer formation unless bridging ligands are used. Photoluminescence from the unsupported stacked dimers extends into the near-infrared, and triplet excited-state lifetimes on the order of 60 ns are observed, representing a substantial enhancement relative to the nonemissive monomers. Such fine control over aggregation, established through deliberate ligand design, allows systematic tuning of properties that are critical for applications in luminescence, photocatalysis and imaging.^65^

## 2 Results and Discussion

### 2.1 Molecular Design and Prototype Complex

In previous work, we developed aryl isocyanide chelating ligands to access photoactive complexes of first- and second-row transition metals that mimic the photophysical and photochemical properties of precious metal-based complexes,^63,64,66^ albeit efforts concentrated on mononuclear complexes. The bi- and tridentate chelating aryl isocyanides that we developed often incorporate a central *meta*-terphenyl backbone,^67,68^ which is favorable for complex stability and photoactive properties. While bidentate aryl isocyanide ligands are relatively easy to synthesize, tridentate ligands that coordinate meridionally, so-called pincer ligands, required significantly more effort.^63,64^ Against this background, we report here a modular synthetic route for tridentate ligand backbones. This approach allows facile interchange of substitution positions and auxiliary groups, and it also enables the simple expansion of the pincer-type ligand library and their use in controlling the dimerization of Rh^I^ complexes.

The multistep synthesis (Scheme 2) involves the borylation of commercial 2,6-dibromo-4-(methyl)­aniline followed by Suzuki-Miyaura cross coupling with 1-bromo-3-iodobenzene, introducing the *meta-*linker between eventual isocyanide-bearing phenyl rings. Subsequent coupling with borylated 2-bromo-4,6-dimethylaniline (**1**) yields the trianiline product **4**. Formylation of the amine groups with acetic formic anhydride and subsequent dehydration with POCl_3_ afforded the isocyanide groups. The product is a *C*,*C*,*C*-tridentate isocyanide ligand shown in Scheme 2 as ***meta*** **-Me**, isolated as a white, odorless, air- and moisture-stable powder. The route proceeds in excellent yields and is amenable to gram-scale synthesis. NMR spectroscopy confirms the assigned structure (see Section S.2.1), consistent with previously reported multidentate isocyanides.^63,64^

**Scheme 2:**
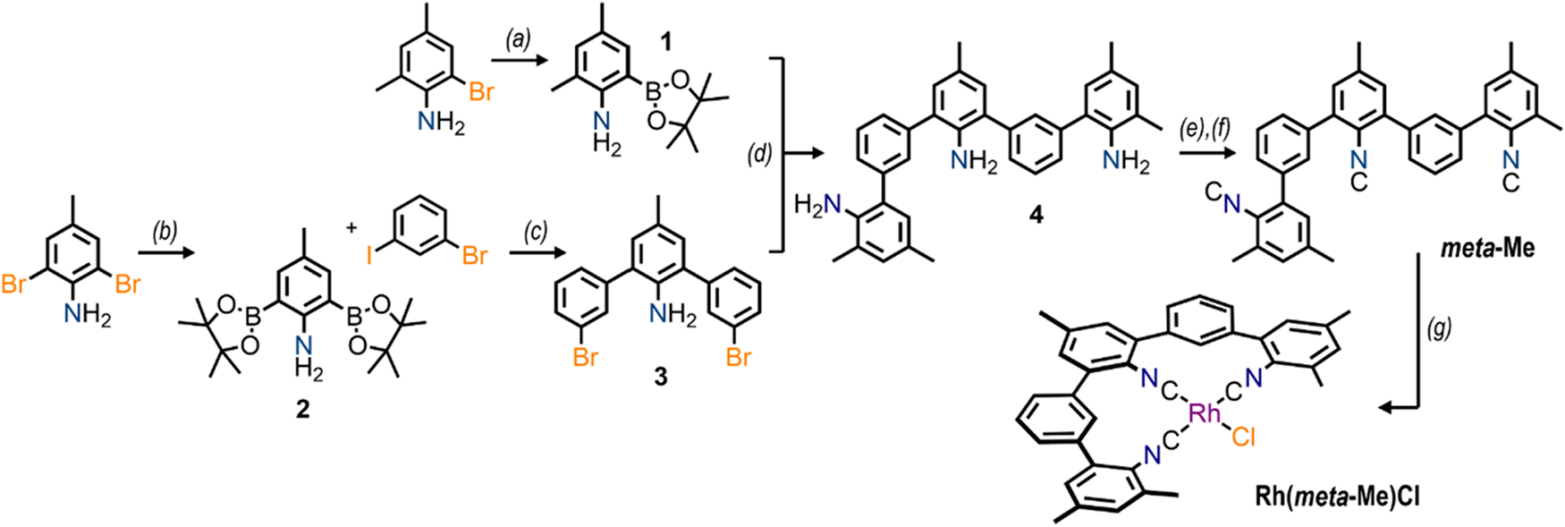
Synthesis of ***meta*** **-Me** and **Rh­(*meta*-Me)­Cl^a^**

a Reagents and conditions: (*a*) Pd­(dppf)­Cl_2_·CH_2_Cl_2_, B_2_Pin_2_, KOAc, 1,4-dioxane, 85°C, 18 h, 63%. (*b*) Pd­(dppf)­Cl_2_·CH_2_Cl_2_, B_2_Pin_2_, KOAc, 1,4-dioxane, 90°C, 18 h, 72%. (*c*) 1-bromo-3-iodobenzene, Pd­(dppf)­Cl_2_·CH_2_Cl_2_, K_2_CO_3_, THF: H_2_O, 65°C, 18 h, 68%. (*d*) Pd­(dppf)­Cl_2_·CH_2_Cl_2_, K_2_CO_3_, DMF, 110°C, 2 h, 80%. (*e*) Acetic formic anhydride, THF, 25°C, 18 h, 86%. (*f*) POCl_3_, NEt_3_, THF, 0°C, 16 h, 85%. Overall yield: 18%. (*g*) [Rh­(COD)­Cl]_2_, CH_2_Cl_2_, 25°C, 1 h, 91%.

The rhodium­(I) complex was synthesized in a simple one-pot reaction of ***meta*** **-Me** with 0.5 *eqv*. [Rh­(COD)­Cl]_2_ in CH_2_Cl_2_ under aerobic conditions (COD = 1,5-cyclooctadiene, Scheme 2, step (*g*)), affording **Rh­(*meta*-Me)­Cl** as a pale-yellow powder in 91% yield. Coordination was confirmed by ^1^H NMR spectroscopy, where phenyl resonances on the coordinating side arm shifted from δ 7.17 and 7.14 in ***meta*** **-Me** to δ 7.13 and 7.03 in **Rh­(*meta*-Me)­Cl**. The ^13^C­{^1^H} NMR spectrum showed two new doublets at δ 151 and 148 (∼2:1 ratio) with coupling constants of 58 and 66 Hz, independent of field strength. These are assigned to the *cis* and *trans* (respective to chloride) isocyanide ^13^C environments (^1^ *J*_Rh–C_, ^103^Rh, I = 1/2), consistent with reported Rh^I^ isocyanide systems.^23,42,69^ High-resolution mass spectrometry (HRMS) and elemental analysis further support the proposed structure of **Rh­(*meta*-Me)­Cl** (Scheme 2).

Unlike previously reported *N*-donor ligated rhodium­(I)-chloride complexes that exhibit stacking,^70^ **Rh­(*meta*-Me)­Cl** shows no such behavior. When measured using concentration-dependent UV–vis absorption spectroscopy (100 μM → 2 mM) in either MeCN, DMSO or CH_2_Cl_2_ solvents, only absorptions at 330 and 410 nm were recorded (Figure S48b). The most important observation is the absence of absorption bands at wavelengths longer than 520 nm, which suggests the lack of significant metal–metal interactions. The 330 nm band is assigned to intraligand (π–π*) absorption bands, while the 410 nm band is commonly attributed to 4d_*z* ^2^_(Rh) → 5p_*z*_(Rh) transitions with admixed metal-to-ligand charge transfer (MLCT) character. The shared symmetry between 5p_*z*_(Rh) and the relevant π* orbital of the isocyanide ligand leads to an overall [4d_*z* ^2^_(Rh) → 5p_*z*_(Rh)/π*­(isocyanide)] transition.^18,19,23^ These absorption bands display linear Beer–Lambert behavior across all concentrations (Figure S48a) and solutions remained uniformly yellow throughout. These data are consistent with the presence of only monomeric species and the absence of stacking events.

### 2.2 Promoting Complex Stacking

Our molecular design leaves the fourth coordination site, occupied by the auxiliary chloride ligand, open to variation. Halide substitution on **Rh­(*meta*-Me)­Cl** was first explored to promote aggregation. Treatment of **Rh­(*meta*-Me)­Cl** with commercially available 2,6-dimethylphenyl isocyanide (**1-CN**) and KPF_6_ in CH_2_Cl_2_ afforded the four-coordinate complex **[Rh­(*meta*-Me)­(1-CN)]­[PF**_**6**_ **]** (Figure 1a). The solution remains yellow during this reaction, and an amorphous, pale-yellow material was isolated. In CD_2_Cl_2_, the ^1^H NMR spectrum now displays additional resonances at δ 7.32 (t, 1H), 7.19 (d, 2H), and 2.44 (s, 6H), attributable to coordinated **1-CN**, alongside minor shifts in the **Rh­(*meta*-Me)** ^**+**^ fragment (e.g., central isocyanide–phenyl ring protons at δ 7.35 to 7.21). The ^13^C­{^1^H} NMR spectrum revealed a new resonance at δ 149 (^1^ *J*_Rh–C_ = 57 Hz) consistent with the bound isocyanide carbon of **1-CN**. Together, these data confirm successful auxiliary ligand exchange.

**Figure 1:**
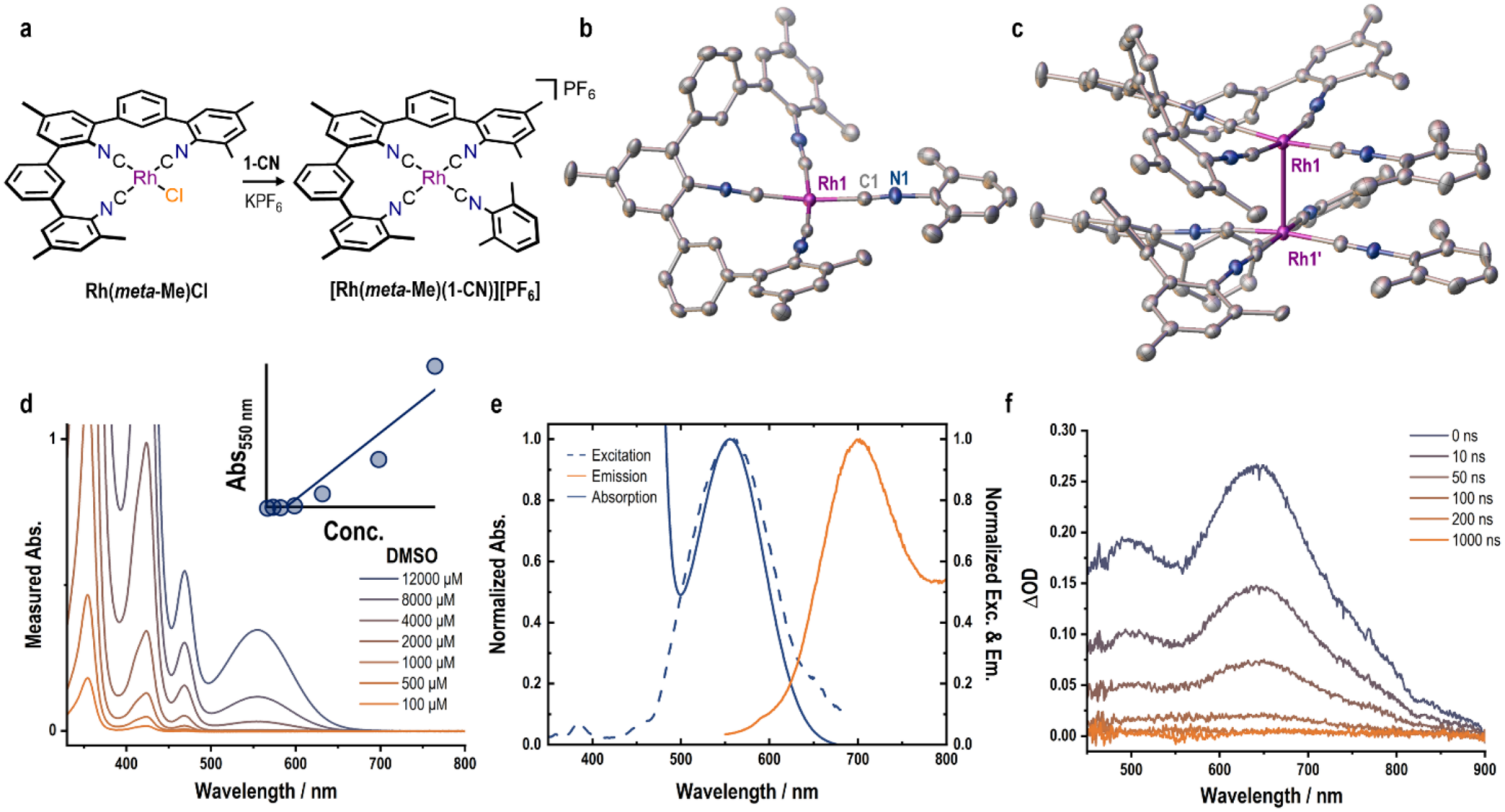
(a) Synthesis of **[Rh­(*meta*-Me)­(1-CN)]­[PF**_**6**_ **]**. Solid-state structure of **[Rh­(*meta*-Me)­(1-CN)]** ^**+**^ highlighting (b) molecular connectivity of one **[Rh­(*meta*-Me)­(1-CN)]** ^**+**^ unit within the (c) dimeric stack. Hydrogen atoms and PF_6_^–^ counteranions removed for clarity, displacement ellipsoids at 50%. (d) UV–vis steady state absorption spectra (100 → 12000 μM, 293 K, DMSO) of **[Rh­(*meta*-Me)­(1-CN)]­[PF**_**6**_ **]**. The inset illustrates the deviation from the Beer–Lambert law at 550 nm. The concentrations shown in the inset correspond to those indicated in the legend. (e) UV–vis absorption (blue, solid), excitation (blue, dashed) and fluorescence emission (orange, solid) traces (4 mM, 293 K, degassed DMSO) of **[Rh­(*meta*-Me)­(1-CN)]­[PF**_**6**_ **]** following excitation at 532 nm. (f) Transient absorption spectra of **[Rh­(*meta*-Me)­(1-CN)]­[PF**_**6**_ **]** (4 mM, 293 K, degassed DMSO), time-integrated over 50 ns after the indicated delays following excitation at 532 nm.

Single crystals of **[Rh­(*meta*-Me)­(1-CN)]­[PF**_**6**_ **]** suitable for X-ray diffraction were obtained by MeCN: Et_2_O layering. The asymmetric unit contains a single cation–anion pair, confirming ***meta*** **-Me** connectivity, overall +1 charge and chloride substitution with **1-CN** (Figure 1b). Rh–C bond lengths (1.951(9)–2.002(9) Å) and C–N bond distances (1.123(12)–1.160(11) Å) are consistent with strong isocyanide triple-bond character and align with reported values.^18,42,57^ The complex adopts an expected square-planar coordination, although with significant deviations from planarity. The central isocyanide–phenyl ring of ***meta*** **-Me** is twisted ∼33° out of plane (relative to a perfect square-planar structure), propagating distortions into the side arm isocyanide–phenyl rings (∼38°). This geometric strain gives *cis*–C–Rh-C angles of 85.7(3)° and 86.0(3)°. We attribute this significant distortion from perfectly square-planar coordination geometry to constrictive distance imposed by the two *meta*-linked peripheral coordination units of ***meta*** **-Me**, preventing flexibility required for a fully planar system.

**[Rh­(*meta*-Me)­(1-CN)]** ^**+**^ units crystallize as dimeric pairs (Figure 1c). The intradimer Rh···Rh distance is 3.1596(12) Å, well within appropriate distances for d_*z* ^2^_ orbital interactions (∼3.1 Å to ∼ 3.3 Å).^19,71^ π–π stacking is only observed between **1-CN** phenyl rings (∼3.6 Å), and is absent between ***meta*** **-Me** phenyl groups, consistent with their twisted geometry. The π–π stacking observed exclusively on the **1-CN** substituents may explain the lack of aggregation in **Rh­(*meta*-Me)­Cl**. **1-CN** may further act to modulate electron density at the Rh^I^ center, a known factor affecting self-assembly.^37^ Each individual Rh···Rh dimer is separated by ∼5.7 Å from a neighboring dimer which, along with Rh···Rh···Rh angles of ∼138°, demonstrating the nonmolecular wire characteristics of the resulting structure (see Figure S66).^55^

UV–vis spectroscopy of **[Rh­(*meta*-Me)­(1-CN)]­[PF**_**6**_ **]** reveals clear evidence of concentration-dependent aggregation. In dilute DMSO solutions (<1 mM), multiple bands between 465–330 nm are observed, assigned to monomer-based transitions (Figure 1d). Upon increasing concentration (1 mM → 12 mM), these features persist with the emergence of a new band at 550 nm (Figure 1d), accompanied by a visible color change from pale yellow to red orange. The 550 nm band is characteristic of Rh^I^···Rh^I^ stacked dimers, and is attributed to a 4dσ*­(Rh_2_) → 5pσ­(Rh_2_)/π*­(isocyanide) transition,^19,30^ consistent with the dimeric stacking seen in the solid state. This 550 nm absorption band shows a clear deviation from Beer–Lambert behavior (Figure 1d, inset).^23^ A similar concentration dependence is observed in MeCN (Figure S49a) yet little evidence of stacking is detected in dichloromethane, even at solubility limits (12 mM, Figure S49c). This suggests a requirement of polar solvents for stacking, likely driven by solvophobic nature of the methyl substituents at the periphery of ***meta*** **-Me**.

Despite evidence for dimer formation, **[Rh­(*meta*-Me)­(1-CN)]­[PF**_**6**_ **]** displays a relatively low stacking propensity. No significant 550 nm absorption is observed below 2 mM, whereas previous systems,^11,12,23^ including our recently reported macrocyclic system,^36^ show strong dimer absorption bands at concentrations as low as 50 μM. A monomer–dimer equilibrium model was applied to quantify the stacking behavior of **[Rh­(*meta*-Me)­(1-CN)]­[PF**_**6**_ **]**.^18,19,23^ A linear relationship from a dimerization plot ([[Rh]/(A_550_)^0.5^) *vs* (A_550_)^0.5^], where [Rh] = total concentration of **[Rh­(*meta*-Me)­(1-CN)]­[PF**_**6**_ **]** and A_550_ is the measured absorbance at 550 nm, confirms the dimeric character above 2 mM (Figure S49). The equilibrium constant was measured at log­(*K*_d_) = 1.0 with Δ*G* = −5.9 kJ mol^–1^ in acetonitrile. This is significantly weaker than typical reported values of ∼−20 kJ mol^–1^.^23^ The weak stacking tendency is attributed to the twisted geometry of **[Rh­(*meta*-Me)­(1-CN)]** ^**+**^, which hinders close metal···metal contacts. Furthermore, only discrete dimers are formed in polar solvents; no signatures of higher aggregates (600–750 nm)^18,23,72^ are detected. This restriction is again consistent with the distortion imposed by the ***meta*** **-Me** framework and relatively poor stacking thermodynamics.

The preference of **[Rh­(*meta*-Me)­(1-CN)]­[PF**_**6**_ **]** for the formation of discrete dimers enables detailed photophysical characterization without significant interference from higher oligomers. Photoexcitation in degassed DMSO at 532 nm yields a fluorescence emission at 705 nm (Figure 1e) with a lifetime of 112 ps (Figure S52), consistent with reported dimeric species.^18,19,44,73–75^ Transient absorption (TA) spectroscopy shows a strong excited-state absorption with overlapping ground-state bleach in the spectral region of the dimer-related (550 nm) band (Figure 1e). Kinetic analysis at 500 nm reveals a single-exponential decay with τ ∼ 67 ns, attributable to a triplet excited-state (T_1_) that is populated from the fluorescent S_1_ state *via* intersystem crossing.^36,47^ Any phosphorescence from this aggregate lies beyond the detection range of the photomultiplier tube employed for this experiment. Solvent-controlled aggregation of this complex thus offers access to excited-state properties beyond those of isolated monomers: A fluorescent S_1_ state and a longer-lived T_1_ state. Due to spectral overlap between the absorption of the excited state and the bleaching of the ground state across the entire detectable region, we could not determine the quantum yield for intersystem crossing using relative actinometry.^76^

### 2.3 Strengthening Stacking Interactions

To address the limited stacking propensity of **Rh­(*meta*-Me)** ^**+**^, we investigated the possibility of forcing dimerization using a bidentate isocyanide ligand that acts as a molecular bridge between two monomers. This methodology has been successfully employed before with small bite-angle (nonisocyanide) bidentate ligands in Pt^II^ complexes^22,28,40,41^ and similar Rh^I^ systems.^31,42–44^ Guided by these precedents,^77^ we developed the wider bite-angle bidentate isocyanide of [1,1′-biphenyl]-3,3′-diisocyanide, **2-CN** (Figure 2a), readily synthesized in only three steps (see Section S.2.4.).

**Figure 2:**
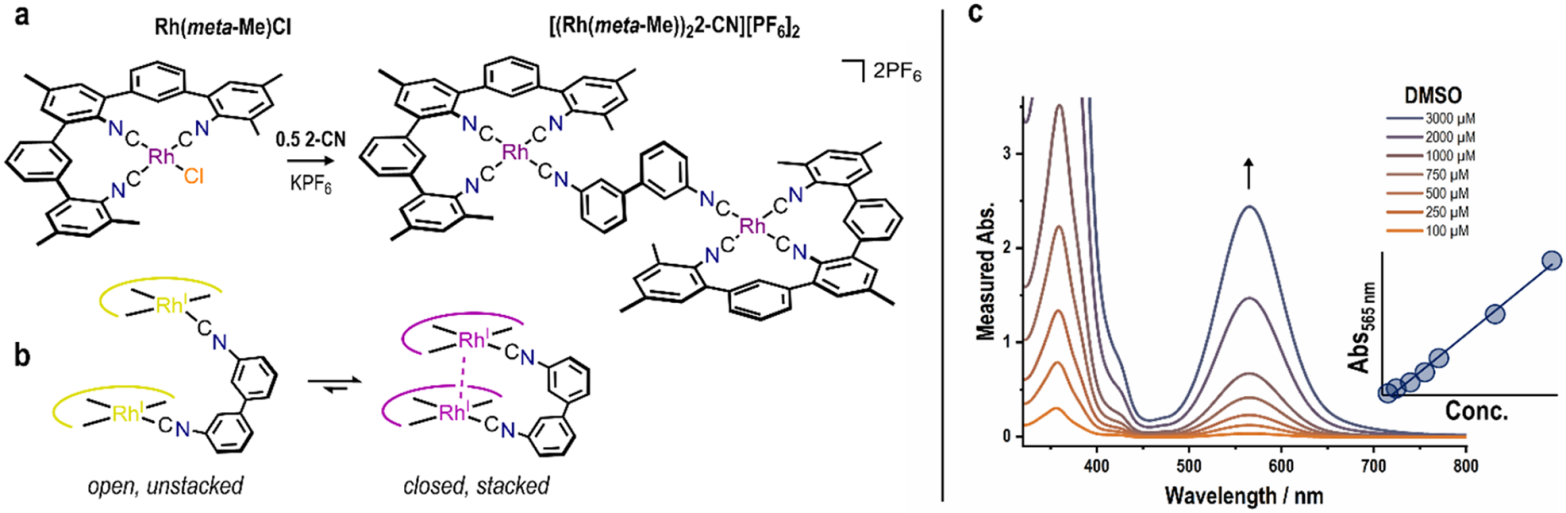
(a) Synthesis of **[(Rh­(*meta*-Me))**_**2**_ **(2-CN)]­[PF**_**6**_ **]**_**2**_, displayed in unstacked arrangement and (b) showing plausible open and closed clamshell stacking arrangement. (c) UV–vis steady state absorption spectra (100 → 3000 μM, 293 K) of **[(Rh­(*meta*-Me))**_**2**_ **(2-CN)]­[PF**_**6**_ **]**_**2**_ in DMSO. The inset illustrates the relationship with Beer–Lambert law at 565 nm. The concentrations shown in the inset correspond to those indicated in the legends.

Addition of 0.5 *eqv*. **2-CN** to **Rh­(*meta*-Me)­Cl** and KPF_6_ in CH_2_Cl_2_ immediately generated a dark-purple solution. The ^1^H NMR spectrum at high concentration is broad and poorly defined, however dilution yields a yellow-orange solution with well-resolved signals (Figure S41). The ^1^H NMR spectrum measured under diluted conditions matches the expected number, integration, and coupling patterns for a cationic species featuring a single **2-CN** bridge between two **Rh­(*meta*-Me)** ^**+**^ units. HRMS supports this assignment ((calc. for [(Rh­(*meta*-Me))_2_(2-CN)]^2+^): *m*/*z* 732.1765 (732.1755), see Figure S42), with EA analysis consistent, confirming **[(Rh­(*meta*-Me))**_**2**_ **(2-CN)]­[PF**_**6**_ **]**_**2**_ (Figure 2a). High-concentration broadening prevents meaningful ^13^C­{^1^H} NMR characterization in this case.

The visible color change and the NMR broadening indicate that aggregation again is concentration related in DMSO, confirmed with UV–vis spectroscopy. At low concentrations (100 μM, DMSO, yellow solution), the spectrum resembles that of **[Rh­(*meta*-Me)­(1-CN)]** ^**+**^, showing mostly monomer-assigned bands between 465 and 350 nm (Figure 2c). At these low concentrations, we suggest **[(Rh­(*meta*-Me))**_**2**_ **(2-CN)]­[PF**_**6**_ **]**_**2**_ adopts an open-faced structure (left part of Figure 2b) with no metal–metal interactions. Increasing concentration to 3 mM (purple solution) generates an aggregated state (right part of Figure 2b), exhibiting a strong absorption band at 565 nm characteristic of a dimeric, Rh^I^···Rh^I^ interaction.^23^ This is again assigned to a 4dσ*­(Rh_2_) → 5pσ­(Rh_2_)/π*­(isocyanide) transition.^19,30^

The precise conformational configuration of this aggregated dimer is not definitively determined. A simple rotation around the biphenyl coupling bond within coordinated **2-CN** results in the switching on and off the Rh^I^···Rh^I^ interactions, however this is typically common for longer, alkyl or arene based linkers.^78–80^ Literature precedent also suggests **[(Rh­(*meta*-Me))**_**2**_ **(2-CN)]­[PF**_**6**_ **]**_**2**_ forms a clamshell arrangement (Figure 2b), which opens and closes the structure with respect to solvent and concentration, enabling and disabling stacking events.^28,29,81–83^ This, mechanistically, operates *via* a pivot-hinge located within the **2-CN** backbone (Figure 2b).^84–88^ In addition, a simple intermolecular stack between two **[(Rh­(*meta*-Me))**_**2**_ **(2-CN)]** ^**2+**^ units is further plausible.^89^ Aggregation from CH_2_Cl_2_ induces stacking aligned to classical rhodium­(I) stacked systems,^5^ exhibiting additional shouldering absorbance bands at ∼675 nm (indicative of possible trimeric-stacked form),^23^ as well as broader features at even longer wavelengths, suggesting higher order oligomers. These do not conform to either pivot-hinge or rotational dimer models, and this may suggest that a simple, intermolecular stack form is predominant in dichloromethane solvent, which further highlights the strong solvent dependence of stacking.

Regardless of the structural model for aggregation, a significant measurement of the 565 nm band is recorded for **[(Rh­(*meta*-Me))**_**2**_ **(2-CN)]** ^**2+**^ as low as 250 μM, while **[Rh­(*meta*-Me)­(1-CN)]­[PF**_**6**_ **]** requires 4 mM to induce stacking. A monomer–dimer equilibrium constant (log­(*K*_d_)) for **[(Rh­(*meta*-Me))**_**2**_ **(2-CN)]­[PF**_**6**_ **]**_**2**_ in DMSO is measured at 4.8 and Δ*G*_DMSO_ = −19.1 kJ mol^–1^ (see Figure S53), is in line with standard reported values.^18,19,23^ In acetonitrile moreover, **[(Rh­(*meta*-Me))**_**2**_ **(2-CN)]­[PF**_**6**_ **]**_**2**_ behaves as concentration-independent aggregate, and is constantly stacked (see Figure S53). This is juxtaposed to the relatively small Δ*G*_MeCN_ (−5.9 kJ mol^–1^) present for **[Rh­(*meta*-Me)­(1-CN)]­[PF**_**6**_ **]**. Switching to the **2-CN** bridging ligand has markedly enhanced stacking, therefore. This suggests the poor aggregation nature of **[Rh­(*meta*-Me)­(1-CN)]** ^**+**^ is likely entropically hindered, as opposed to stacking being only thermodynamically limited *via* its warped ligand structure.

Fluorescence emission from aggregated **[(Rh­(*meta*-Me))**_**2**_ **(2-CN)]­[PF**_**6**_ **]**_**2**_ is measured at 700 nm in DMSO with a lifetime of 109 ps (Figure S56), analogous to stacked **[Rh­(*meta*-Me)­(1-CN)]­[PF**_**6**_ **]** and other reported dimeric systems.^11,12,23^ The fluorescence lifetime was modeled with a biexponential decay, where we attribute a second, minor (0.4%) component to higher-order oligomers that cannot be detected in the UV–vis spectra. Transient absorption measurements reveal intense excited-state absorption between 500–630 nm, with kinetic analysis at 500 nm yielding a first-order decay of τ ∼ 64 ns for the relevant T_1_ excited state. Thus, simple solvent-based aggregation has again provided access to an excited-state lifetime exceeding that of the isolated monomers (in which no evidence for the formation of a long-lived triplet excited state was found), which is potentially relevant for photocatalysis. Absorbance, emission and TA spectroscopy indicate that the Rh^I^···Rh^I^ contacts are essentially identical compared to the discrete, well-defined **[Rh­(*meta*-Me)­(1-CN)]** ^**+**^ stacked dimer in polar solvents.

### 2.4 Modulation of Stacking through Bite-Angle Changes

Bite-angles within chelate ligands are known to influence complex stability, reactivity,^61,62^ and photophysical properties.^90–92^ To explore this in the context of supramolecular aggregation, and to potentially relieve the strained, buckled nature of **Rh­(*meta*-Me)** ^**+**^ (Figure 1c and Scheme 2), we targeted a more facially open system. Exchange of the linker phenyl ring (1,3-substituted in ***meta*** **-Me**) to a 1,4-substituted ring achieves this. This molecular design, which uses *para*- instead of *meta*-phenyl linkages, represents a significant departure from the design principles previously investigated for chelating aryl isocyanide ligands.^66,93^

Following the methodology outlined in Scheme 2, 1-bromo-3-iodobenzene was replaced with 1-bromo-4-iodobenzene in step (*c*), installing a *para*-substituted phenyl linker between the central aryl isocyanide unit and the two peripheral aryl isocyanides. Upon completion of the synthesis following the same steps and using the same methyl-substituted reagents, the new ligand, ***para*** **-Me** (Figure 3a), was prepared again on half-gram scale as a colorless, air-stable solid in excellent yields (see Section S.2.2.).

**Figure 3:**
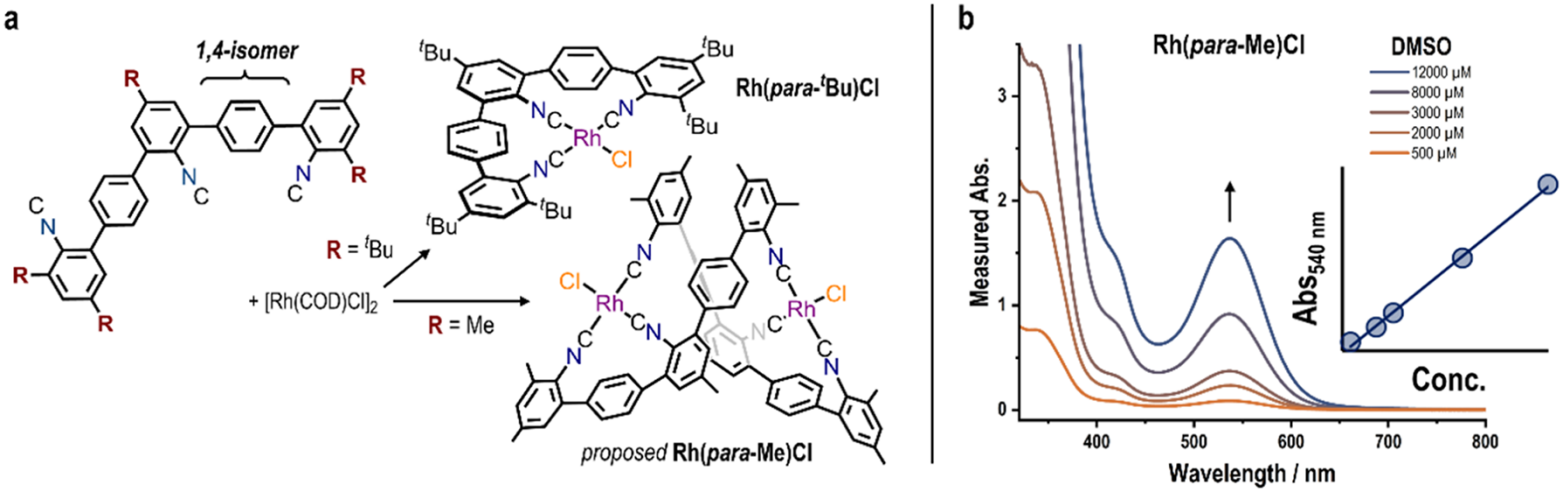
(a) Synthesis of **Rh­(** ***para*** **-Me)­Cl** and **Rh­(** ***para*** **-** ^***t***^ **Bu)­Cl**. (b) UV–vis steady state absorption spectra (0.5 → 12 mM, 293 K) of **Rh­(** ***para*** **-Me)­Cl** in DMSO. The inset illustrates the validity of the Beer–Lambert law at 540 nm. The concentrations shown in the inset correspond to those indicated in the legend.

Reaction of ***para*** **-Me** with 0.5 *eqv*. [Rh­(COD)­Cl]_2_ in dichloromethane immediately produced a deep purple solution (*cf*. pale yellow with ***meta*** **-Me**). ^1^H NMR spectra in CD_2_Cl_2_ and DMSO-*d*_6_ were poorly resolved (see Figure S43), consistent with the stacked complexes observed here and previously.^54^ The deep purple color was retained in all solvents, as well as isolated solid material. Elemental analysis and HRMS data indicate a 1:1:1 Rh: ***para*** **-Me**: Cl stoichiometry, suggesting the formation of the complex **Rh­(** ***para*** **-Me)­Cl** (Figure 3a).

UV–vis absorption spectroscopy in DMSO reveals a discrete absorption band at 540 nm, consistent with dimeric 4dσ*­(Rh_2_) → 5pσ­(Rh_2_)/π*­(isocyanide) transitions (Figure 3b). No deviation from Beer–Lambert behavior is detected from 500 μM → 12 mM, as well as no higher order oligomeric bands in DMSO solvent (Figure 3b), indicating **Rh­(** ***para*** **-Me)­Cl** exists as concentration-independent dimer. The intense purple color throughout and broad NMR signals are further consistent with a permanently stacked formation. Furthermore, the 540 nm band is also observed in dichloromethane and MeCN solvents, demonstrating that stacking is independent of both solvent and concentration.

Diffusion-ordered spectroscopy (DOSY)^94^ was used to determine the diffusion coefficients of isomeric complexes **Rh­(*meta*-Me)­Cl** and **Rh­(** ***para*** **-Me)­Cl**. In DMSO-*d*_6_ solvent, diffusion coefficients of 1.519 × 10^–10^ m^2^ s^–1^ and 1.112 × 10^–10^ m^2^ s^–1^ were measured, respectively. As **Rh­(*meta*-Me)­Cl** is monomeric in DMSO solvent, the resulting relative-volume ratio is 2.55 for **Rh­(** ***para*** **-Me)­Cl**, suggesting that the *para* isomer exists as predominantly larger species in solution—consistent with dimeric suggestion from UV–vis absorption measurements. This inferred dimer exhibits a slightly expanded hydrodynamic volume, potentially arising from the specific stacking arrangement and/or the sensitivity of the hydrodynamic radius to subtle structural variations.^36,59^ Overall, this switching of 1,3- (*meta*) to 1,4- (*para*) substituted phenyl linkers has therefore enabled metal–metal interactions from the corresponding complex. Clearly, there is a stark contrast between **Rh­(*meta*-Me)­Cl**, where no stacking events were initiated, and **Rh­(** ***para*** **-Me)­Cl**, which readily dimerizes. This highlights how pivotal ligand construction is within photoactive complex designs.

Single-crystals of **Rh­(** ***para*** **-Me)­Cl** suitable for X-ray diffraction analysis were regrettably not achieved, inhibiting precise information upon the structural conformation. To overcome this, and knowing the sensitivity of aggregation to ligand modifications,^18,35^ we explored bulkier substituents within the ligand backbone. Following the procedure outlined in Scheme 2, upon substituting 2-bromo-4,6-di­(methyl)­aniline for 2-bromo-4,6-di­(*tert*-butyl)­aniline in step (*a*), and 2,6-dibromo-4-(*tert*-butyl)­aniline in step (*b*), this affords ***para*** **-** ^***t***^ **Bu**, a novel ligand that retains the skeletal framework and bite angle of ***para*** **-Me** but replaces all methyl groups with *tert*-butyl substituents.

Addition of 0.5 *eqv*. of ***para*** **-** ^***t***^ **Bu** to [Rh­(COD)­Cl]_2_ in dichloromethane yielded a pale-yellow solution with fully resolved ^1^H and ^13^C­{^1^H} NMR spectra signals (see Section S.3.5.). This new complex, **Rh­(** ***para-*** ^***t***^ **Bu)­Cl**, shows no evidence for stacking, as indicated by the absence of bands above 465 nm in UV–vis absorption spectra in MeCN, DMSO, or dichloromethane. DOSY measurements reveal a ∼1:1 diffusion coefficient volume-ratio with **Rh­(*meta*-Me)­Cl** (see Table S1), also indicating the monomeric nature. Stacking inhibition here is simply assigned to the bulky ^*t*^Bu groups, preventing close Rh···Rh contacts.

Crystallization of **Rh­(** ***para-*** ^***t***^ **Bu)­Cl** was achieved with slow addition of acetonitrile into a concentrated dichloromethane solution. Upon analysis with single-crystal X-ray diffraction analysis, the structure shown in Figure 4 confirms the expected ligand stoichiometries, however with unexpected interconnecting configuration. This arrangement is formed from one isocyanide decoordinating, and bridging to a second Rh center, which reciprocally coordinates back to the first. Analogous interconnected-dimeric structures are also reported in pincer pyrrolate-type platinum­(II) complexes.^95^ The solution NMR data collected in dichloromethane indicates that **Rh­(** ***para*** **-** ^***t***^ **Bu)­Cl** exists as a solution based-monomer; so this structure therefore is not representative of the solution species. Interconversion between monomer and dimer pincer pyrrolate-type platinum­(II) was facilitated with additional coordinating ligands, *via* an associative substitution mechanism.^95^ We suggest this interconversion also occurs here, where acetonitrile acts as the substitution reagent. The poor solubility of crystalline **Rh­(** ***para*** **-** ^***t***^ **Bu)­Cl** toward acetonitrile however hinders meaningful quantitative analysis of this interconversion and elemental analysis could neither help distinguish these two forms, either.

**Figure 4:**
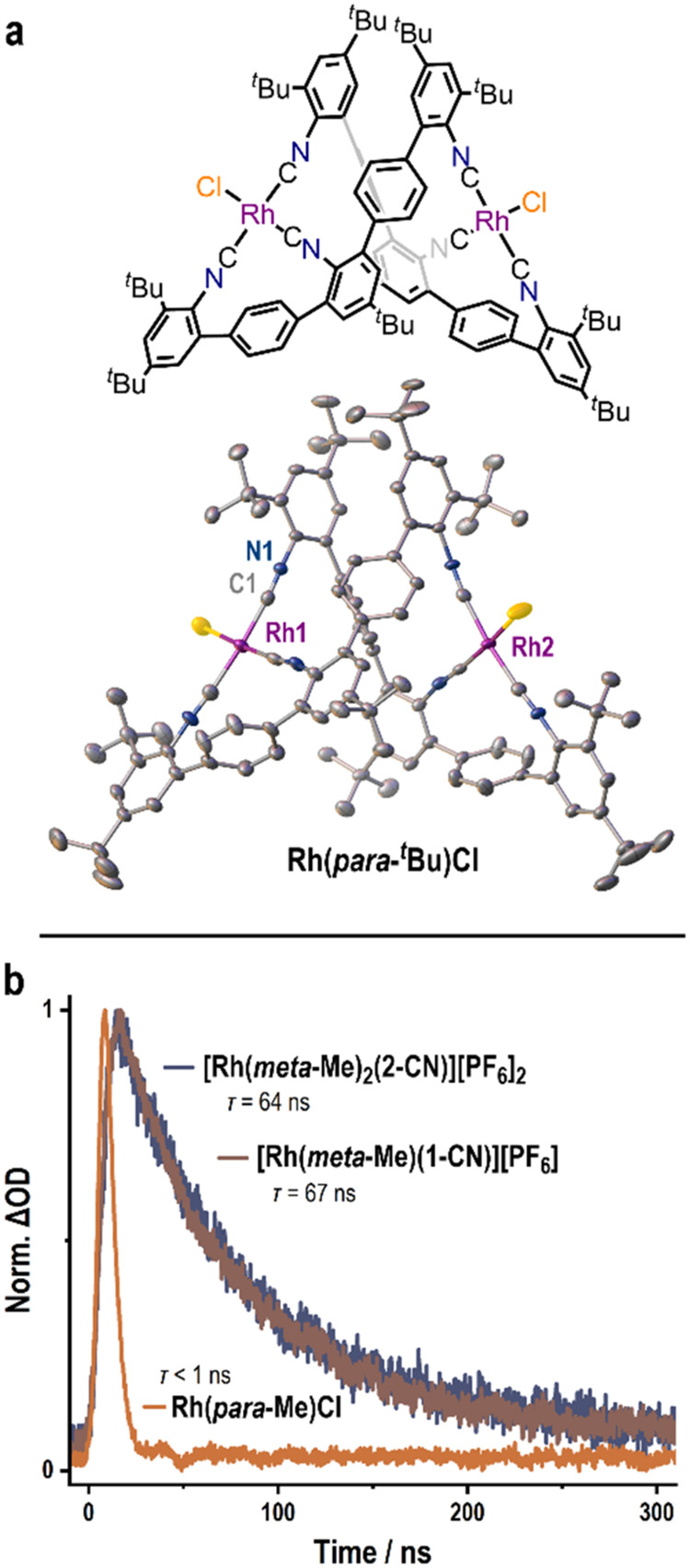
(a) Solid-state structure of **Rh­(** ***para*** **-** ^***t***^ **Bu)­Cl**. Crystals grown by slow diffusion of MeCN into a dichloromethane solution. Hydrogen atoms and solvent molecules removed for clarity. Displacement ellipsoids at 50%. (b) Excited-state absorption (ESA) decays at 500 nm for different complexes in deaerated DMSO at room temperature. All measurements were conducted with 7 mJ pulses with pulse widths of ∼10 ns after excitation at 532 nm.

The interconnected structural configuration of **Rh­(** ***para*** **-** ^***t***^ **Bu)­Cl** observed in the solid-state is tentatively assigned to the unknown aggregated structure discussed above for **Rh­(** ***para*** **-Me)­Cl**. The same interconnected skeletal configuration is present, yet less-bulky methyl groups might allow for discrete, dimeric Rh···Rh interactions to form. The 1,4-phenyl linker within *para*-ligand likely facilitates this geometry by opening the coordination angle and enabling bridged metal centers, inaccessible in *meta*-systems. Unlike **Rh­(** ***para*** **-** ^***t***^ **Bu)­Cl**, the interconnected configuration appears to be present in both solution and solid-state **Rh­(** ***para*** **-Me)­Cl**, highlighted with consistent purple-color and UV–vis measurements. This is likely driven through formally thermodynamically favorable metal–metal interactions,^2^ accessible in **Rh­(** ***para*** **-Me)­Cl** with reduced bulky groups. The discrete dimeric pair observed for **Rh­(** ***para*** **-Me)­Cl** is distinctly different from the *meta* systems described here or for dimers in previous reports.^11,12,23^ Definitive proof that **Rh­(** ***para*** **-Me)­Cl** adopts this configuration has evaded us, however.

The photophysical behavior of **Rh­(** ***para*** **-Me)­Cl** was examined and upon 532 nm excitation in deaerated DMSO, fluorescence emission was observed at 655 nm. The fluorescence in this case decays faster than the instrument response function, indicating a fluorescence lifetime below 65 ps (Figure S61). In transient absorption spectroscopy, there is no evidence for a long-lived triplet excited state, but the observation of a bleach on the ns time scale could reflect a photochemical reaction. This contrasts with **[Rh­(*meta*-Me)­(1-CN)]­[PF**_**6**_ **]**, which displays dimeric absorption bands (∼530 nm), fluorescence emission at 705 nm with a lifetime of 112 ps (Figure S52), and a T_1_ excited state with a lifetime of ∼65 ns, underscoring that the ancillary ligand is essential for achieving longer-lived S_1_ and T_1_ excited states. Attempts to substitute the chloride ligand of **Rh­(** ***para*** **-Me)­Cl**, aimed at promoting intermolecular aggregation and to activate photophysical properties as achieved for **Rh­(*meta*-Me)­Cl** → **[Rh­(*meta*-Me)­(1-CN)]­[PF**_**6**_ **]**, were unsuccessful. Only complex degradation was observed, indicating a more fragile system with the *para*-phenyl ligand, linking to coordination/decoordination observed upon crystallization.

## 3 Conclusions

In summary, we report the synthesis of a family of new isocyanide containing pincer ligands and their subsequent coordination to rhodium­(I). The resulting square-planar complexes exhibit controlled aggregation, forming metal–metal interactions that result in red to near-infrared fluorescence emission and triplet excited states with lifetimes on the order of tens of nanoseconds. The corresponding monomers do not exhibit such photophysical properties, indicating that the fluorescent S_1_ states and the nanosecond-lived T_1_ states are emergent properties arising from aggregation. The ability to control aggregation to produce well-defined dimeric structures rather than ill-defined oligomeric mixtures enables these photophysical insights. Extending beyond the specific complexes studied herein, we demonstrate four distinct molecular design strategies for achieving structural control and enhanced photophysical properties

- Modulating the pincer ligand bite angle (***meta*** **-Me** *vs* ***para*** **-Me**; Scheme 1c).
- Tuning the electronic character of the auxiliary ligand at the fourth coordination site, ranging from π-donor to π-acceptor (Figure 1a).
- Comparing spontaneous aggregation with aggregation assisted by a conformationally flexible bridging ligand (Figure 2a/b).
- Exploring solvophobic and steric effects by varying the alkyl substituents at the ligand periphery (***para*** **-Me** *vs* ***para*** **-** ^***t***^ **Bu**; Figure 3a).

The **Rh­(*meta*-Me)­Cl** complex (Figure 5a) does not undergo aggregation, making it of limited utility in photophysical and photochemical applications. Upon modification of the fourth coordination site, replacing the chloride ligand with an arylisocyanide, the resulting **[Rh­(*meta*-Me)­(1-CN)]** ^**+**^ complex (Figure 5b) exhibits solvent- and concentration-dependent supramolecular stacking behavior. This leads to weak Rh^I^···Rh^I^ interactions, consistent with the formation of discrete dimeric supramolecular assemblies (λ_max_ ∼ 550 nm). A simple change to a conformationally flexible bidentate bridging ligand links two metal complexes directly, enabling the formation of aggregated conformers of **[(Rh­(*meta*-Me))**_**2**_ **(2-CN)]** ^**2+**^ (Figure 5c), which display stronger dimer absorption bands at a similar wavelength. Both aggregates have long-lived triplet excited states (∼65 ns), making them promising candidates for photocatalytic applications, alongside fluorescence in the red to near-infrared spectral range. Tuning the bite angle of the pincer ligand and modulating the steric environment at the ligand by varying alkyl substituents from ***para*** **-Me** to ***para*** **-** ^***t***^ **Bu** ligands leads to further significant changes in structural and photophysical behavior (Figure 5d).

**Figure 5:**
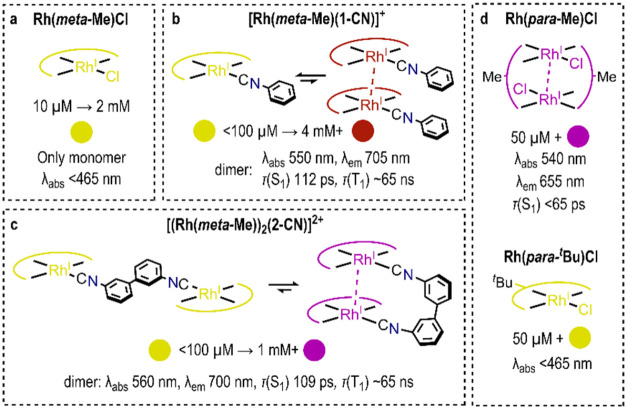
Summary of dimerization processes and key photophysical studies of a) **Rh­(*meta*-Me)­Cl**, b) **[Rh­(*meta*-Me)­(1-CN)]­[PF_6_]**, c) **[Rh­((*meta*-Me))_2_(2-CN)]­[PF_6_]_2_**, d) **Rh­(*para*-Me)­Cl** *vs* **Rh­(*para*-^*t*^Bu)­Cl** in DMSO solvent. λ_abs_ and λ_em_ denote the characteristic UV–visible absorption or fluorescence emission band maxima. τ­(S_1_) denotes the lifetime of the fluorescent S_1_ state, while τ­(T_1_) represents the lifetime of the lowest triplet excited state. The colored filled circles correspond to the solution colors of the respective compounds, with yellowish hues indicating the absence of metal–metal interactions and red-to-purple hues indicating significant metal–metal interactions.

The **Rh­(** ***para*** **-Me)­Cl** complex undergoes concentration-independent dimerization, resulting in a slightly blue-shifted absorption maximum (λ_max_ ∼ 540 nm) and a short-lived S_1_ excited state (<65 ps). Although the exact solution structure of these dimers remains unclear, X-ray structural analysis of **Rh­(** ***para*** **-** ^***t***^ **Bu)­Cl** indicates that the tridentate ligand no longer behaves as a classical pincer, as in the corresponding monomers. Instead, it covalently links two Rh^I^ coordination units, likely facilitated by acetonitrile-induced ligand exchange reactions that reorganize the primary coordination spheres upon dimer formation (Figure 4a). Variation in the peripheral alkyl substituents further distinguish between dimers lacking metal–metal interactions (with the ***para*** **-** ^***t***^ **Bu** ligand; Figure 4a) and those exhibiting such interactions to varying degrees (with the ***para*** **-Me** ligand; Figure 3a).

The observed differences in stacking propensity and excited-state lifetimes underscore the sensitivity of metal–metal interactions to subtle ligand modifications. While monodentate systems have typically produced uncontrolled oligomeric mixtures, we have successfully accessed a new series of well-defined dimers through precise molecular design guided by the four clear-cut control factors listed above. These findings establish general design rules for the rational development of photoactive supramolecular aggregates of square-planar d^8^ metal complexes with potential applications in materials science and photochemistry. This work opens new avenues for advancing inorganic photophysics and photochemistry beyond the predominantly studied mononuclear species toward polynuclear systems with emergent properties.

## Supplementary Material



## Notes

Experimental details, crystallographic information, spectroscopic characterization, HR-ESI mass spectra, and photophysical data (PDF)
