# Supplementary material for: Controlled Dimerization of Rhodium(I) Isocyanides Enables Photophysical Properties Beyond Mononuclear Complexes
Source: JACS Au. 2026 May 20;6(6):3494–505. doi: 10.1021/jacsau.6c00461 (PMC13292004; doi:10.1021/jacsau.6c00461)
Supplement: Supplementary file 1 [file au6c00461_si_001.pdf]

Supporting Information

Controlled Dimerization of Rhodium(I) Isocyanides Enables Photophysical Properties Beyond Mononuclear Complexes

Alexander J. Bukvic, Leander Spierling, Daniel Häussinger and Oliver S. Wenger*

Department of Chemistry, University of Basel, St. Johanns-Ring 19, 4056 Basel, Switzerland.

E-mail: oliver.wenger@unibas.ch

TABLE OF CONTENTS

S.1. EXPERIMENTAL DETAILS....S4
S.1.1. General Methods....S4

S.2. ORGANIC SYNTHESIS....S6
S.2.1. *meta*-Me....S6
S.2.1.1. Step A....S7
S.2.1.2. Step B....S8
S.2.1.3. Step C....S9
S.2.1.4. Step D....S11
S.2.1.5. Step E....S13
S.3.1.6. Step F....S15
S.2.2. *para*-Me....S18
S.2.2.3. Step C....S19
S.2.2.4. Step D....S20
S.2.2.5. Step E....S22
S.2.2.6. Step F....S24
S.2.3. *para*-^{t}Bu....S27
S.2.3.1. Step A....S28
S.2.3.2. Step B....S28
S.2.3.3. Step C....S29
S.2.3.4. Step D....S31
S.2.3.5. Step E....S33
S.2.3.6. Step F....S35
S.2.3.7. Step G....S37
S.2.3.8. Step H....S39
S.2.4. [1,1'-biphenyl]-3,3'-diisocyanide, 2-CN....S42
S.4.1.1. Step B....S43
S.4.1.2. Step C....S45

S.3. ORGANOMETALLIC SYNTHESIS....S48
S.4.1. Rh(*meta*-Me)Cl....S48
S.4.2. [Rh(*meta*-Me)(1-CN)][PF_6]....S51
S.4.3. [(Rh(*meta*-Me))$_2$(2-CN)][PF_6]$_2$....S54
S.4.4. Rh(*para*-Me)Cl....S57
S.3.5. Rh(*para*-^{t}Bu)Cl....S58

S.5. OPTICAL SPECTROSCOPY....S61
S.5.1. Rh(*meta*-Me)Cl....S61
S.5.1.1. UV-Vis Spectra....S61
S.5.2. [Rh(*meta*-Me)(1-CN)][PF_6]....S62

S.5.2.1. UV-Vis Spectra S62
S.5.2.2. Emisson Spectra S63
S.5.2.3. Transient Absorption Measurements S63
S.5.2.4. Time-Resolved Emission S64
S.5.3. [(Rh(meta-Me))$_2$(2-CN)][PF$_6$]$_2$ S65
S.5.3.1. UV-Vis Spectra S65
S.5.3.2. Emisson Spectra S66
S.5.3.3. Transient Absorption Measurements S66
S.5.3.4. Time-Resolved Emission S67
S.5.4. Rh(*para*-Me)Cl S68
S.5.4.1. UV-Vis Spectra S68
S.5.4.2. Emisson Spectra S70
S.5.4.3. Transient Absorption Measurements S70
S.5.4.4. Time-Resolved Emission S71
S.5.5. Rh(*para*-^{t}Bu)Cl S72
S.5.5.1. UV-Vis Spectra S72

S.6. DOSY MEASUREMENT DATA S73

S.7. CRYSTALLOGRAPHIC AND REFINEMENT DATA S75
S.7.1. Crystal structure determinations S75
S.7.2. Refinement and Data Tables S75

S.8. REFERENCES S77

S.1. Experimental Details

S.1.1. General Methods

Air sensitive synthetic manipulations were performed under inert conditions, using standard Schlenk techniques on a dual vacuum/inlet manifold or by employment of an MBraun glovebox. Glassware was dried in an oven at 120°C overnight prior to use. Acetonitrile, dichloromethane and diethyl ether were dried using a solvent purification system and degassed by three freeze-pump-thaw cycles. Dimethylsufoxide-d_6 and dichloromethane-d_2 (abbreviated to DMSO-d_6 or CD_2Cl_2 respectively) and all other chemicals were purchased from commercial vendors and used as received.

NMR spectra were recorded using a Bruker Avance III spectrometer operating at proton frequencies of 250, 400, or 500 MHz. Residual protio solvent resonances were used as an internal reference for ^{1}H and $^{13}C\{^{1}H\}$ NMR spectra and externally referenced to $SiMe_4$. $^{31}P\{^{1}H\}$ NMR spectra were referenced externally to 85% H_3PO_4 (D_2O). $^{19}F\{^{1}H\}$ NMR chemical shifts are externally referenced to $CFCl_3$. All chemical shifts (δ) are quoted in ppm. Coupling constants (*J*) are given in Hz and refer to following multiplicities (s = singlet, d = doublet, t = triplet, q = quartet, quin = quintet, m = multiplet, app = apparent, br. = broad signal, dd = doublet of doublets, etc.). The ^{1}H NMR spectra are reported as follows: chemical shift (multiplicity, number of protons, coupling constants). Chemical environments have been assigned through COSY, HSQC/HMBC or NOE NMR spectroscopic experiments and are shown where appropriate.

Self-diffusion measurements were performed with the bipolar gradient pulse sequence from Wu *et al.*[S1] using a Bruker Avance III NMR spectrometer operating 600.13 MHz proton frequency. The instrument is equipped with a 5-mm broadband direct observe BBFO probe with a shielded z-gradient coil and a GAB gradient amplifier (10 Ampere, maximum gradient strength 52.5 G/cm). All samples contained 5% D_2O as lock substance. The diffusion experiments were performed at 298 K and the temperature was calibrated using a methanol standard showing accuracy within +/- 0.2 K. The gradient strength was calibrated using a Shigemi tube filled with H_2O to a height of 4.0 mm and imaging this water cylinder.[S2] The resulting gradient calibration was validated by determining the diffusion coefficient of water at 310 K and reproduced the literature value within 5%.

The diffusion experiments were performed by varying the gradient strength between 5% and 95% of the maximum strength in 32 single experiments using an acquisition time of 2 s while keeping the diffusion times and gradient lengths constant. The diffusion time was set to 30 ms (big delta) and a gradient duration of 1.5 ms was applied (little delta). The intensity decrease of the signal of interest was determined and fitted with a Bruker t1/t2 software package suitable for DOSY experiments, which is included in the Topspin 3.6 software.[S3]

Elemental analysis was performed by Sylvie Mittelheisser, with a Vario Micro Cube instrument from Elementar. HRMS analyses were conducted by Dr. Michael Pfeffer on a Bruker maxis 4G ESI-Q-TOF under direct injection conditions with CH_3CN or THF as solvent.

Thin layer chromatography (TLC) was performed on pre-coated aluminum plates (Merck: silica gel 60 with a fluorescence indicator F254, layer thickness of 0.25 mm). Compound visualization was done under UV light, using either the 254 nm or 365 nm wavelength from a UV lamp. For the isolation of reaction products via column chromatography, silica gel (Silicycle: silica flash® P60, 40-63 µm, 230-400 mesh) was used.

Steady-state UV-Vis absorption spectra were recorded using a Cary 5000 spectrophotometer (Agilent Technologies). Steady-state emission and excitation spectra were recorded on either a Fluorolog-322 instrument (Horiba Jobin-Yvon) equipped with iHR320, a Xenon lamp 450 W Illuminator (FL-1039A/40A) and a water-cooled photomultiplier tube (PMT Hamamatsu R13456), where the cuvette holder was equipped with a temperature controller (TC 1, Quantum Northwest) or a FP-8600 spectrophotometer (JASCO).

Photoluminescence lifetimes with picosecond resolution were conducted using a LifeSpec II from Edinburgh Instruments with a pulsed laser with excitation (EPL Series, Edinburgh Instruments, pulse width at 635 nm: 61.4 ps). Fluorescence emission was accumulated *via* time-correlated single photon counting (TCSPC). The cuvette holder was equipped with a temperature controller (TC 1, Quantum Northwest).

Transient UV-Vis absorption spectroscopy with nanosecond time resolution was performed on an LP920KS apparatus from Edinburgh Instruments. A frequency-tripled Nd:YAG laser (Quantel Brilliant, ca. 10 ns pulse width) equipped with an OPO from Opotek was used for excitation at 355 and 680 nm. An iCCD camera from Andor was used to detect transient absorption spectra, and single-wavelength kinetics were recorded with a photomultiplier tube.

S.2. Organic Synthesis

S.2.1. *meta*-Me

Procedure Overview:

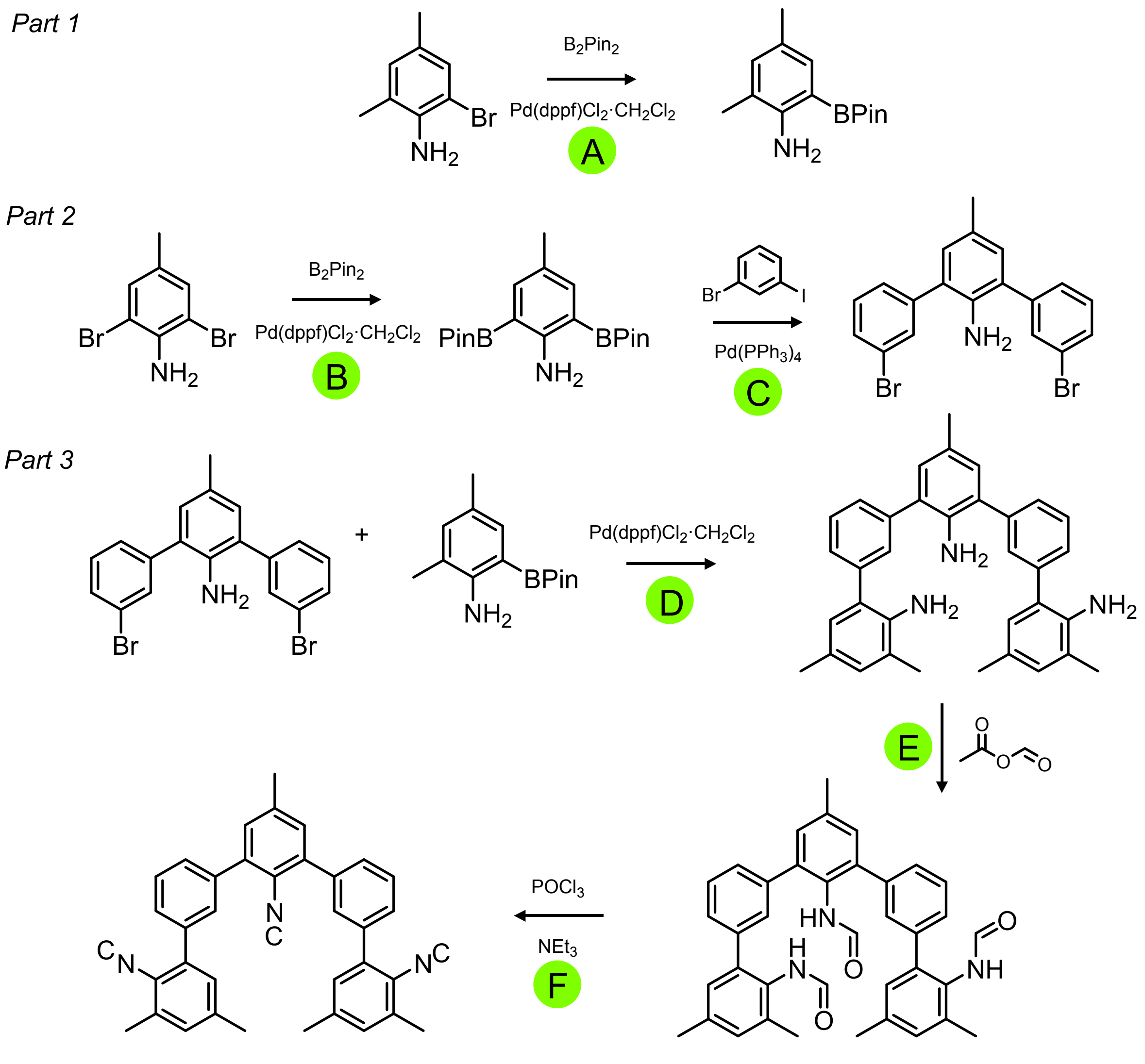

***meta*-Me**

Note: Steps A and B have been reported by Smith *et. al.* (*Nature,* **2023,** *615*, 430)[S4] and Wang *et. al.* (*Macromolecules*, **2015**, *48*, 5504)[S5] respectively, however have been adapted and improved for our purposes in this preparation. Characterization data and spectra are given for all new products beneath each procedure.

S.2.1.1. Step A

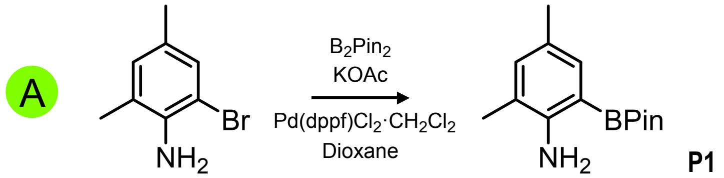

A 250 mL round bottom flask was charged with 2-bromo-4,6-dimethylaniline (4.90 g, 24.5 mmol, 50 *eqv.* M_r: 200), bis(pinacolato)diboron (8.09 g, 31.9 mmol, 65 *eqv.* M_r: 254) and KOAc (7.20 g, 73.5 mmol, 150 *eqv.*, M_r: 98). Dry 1,4-dioxane (100 mL) was added and solution deoxygenated with bubbling N_2 for 30 minutes, before addition of $Pd(dppf)Cl_2 \cdot CH_2Cl_2$ (400 mg, 0.49 mmol, 1 *eqv.*, M_r: 816) and a further 10 minutes degassing. The mixture was heated for 18 hours at 85°C.

After cooling to room temperature, the reaction mixture was poured into ice-cooled distilled water (~200 mL) and left to stir until a permanent precipitate formed. The brown solids were filtered, washed with water (3 × 50 mL) and dissolved in dichloromethane. This solution was dried over anhydrous Na_2SO_4 and solution passed through a short Silica plug. The now pale-yellow solution was concentrated to a minimum (~5 mL) and methanol added (~40 mL). The resulting precipitate was filtered and washed with ~5°C methanol (2 × 5 mL) to yield **P1**. The methanol washings were further combined, concentrated under reduced pressure and left to stand at 5°C for 24 hours. After this time, the crystalline material was filtered to yield **P1** as a white solid (total: 3.80 g, 15.4 mmol, 63%, M_r: 247).

^{1}H NMR ($CDCl_3$, 298 K, 400 MHz): δ 7.51 (d, 1H, $^4J_{HH}$ = 2.3 Hz), 7.40 (d, 1H, $^4J_{HH}$ = 2.3 Hz), 4.95 (br. s, 2H), 2.32 (s, 3H), 2.25 (s, 3H), 1.37 (s, 12H).

S.2.1.2. Step B

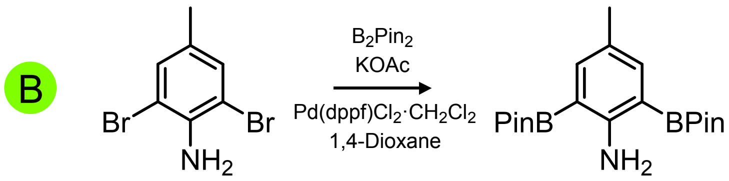

P2

A 250 mL round bottom flask was charged with 2,6-dibromo-4-methyl-aniline (4.00 g, 15.1 mmol, 50 *eqv.*, M_r: 264), bis(pinacolato)diboron (11.5 g, 50.0 mmol, 150 *eqv.,* M_r: 254) and KOAc (8.90 g, 99.9 mmol, 300 *eqv.,* M_r: 98). The solids were placed under a N_2 atmosphere and suspended in dry 1,4-dioxane (80 mL). The solution was deoxygenated with bubbling N_2 for 30 minutes, before addition of Pd(dppf)Cl_2·CH_2Cl_2 (247 mg, 0.30 mmol, 1 *eqv.,* M_r: 816) and a further 10 minutes degassing. The mixture was heated for 18 hours at 80°C.

After cooling to room temperature, the reaction mixture was poured into ice-cooled distilled water (~200 mL) and left to stir until a permanent precipitate forms. The black solids were filtered, washed with water (3 × 20 mL) and dissolved in dichloromethane. This solution was dried over anhydrous Na_2SO_4 then passed through a short Silica plug. The now pale-yellow solution was removed to a minimum (~5 mL) under reduced pressure and product precipitated with methanol (~20 mL). The white solids were collected, washed with cooled (~5°C) methanol (2 × 5 mL) dried under reduced pressure to yield **P2**. The methanol washings were combined, concentrated and left to stand at 5°C for 24 hours. After this time, the crystalline material was filtered to further yield **P2** as a white solid (total: 3.92 g, 10.9 mmol, 72%, M_r: 359).

^{1}H NMR ($CDCl_3$, 298 K, 400 MHz): δ 7.51 (s, 2H), 5.87 (br. s, 2H), 2.18 (s, 3H) 1.32 (s, 24H).

S.2.1.3. Step C

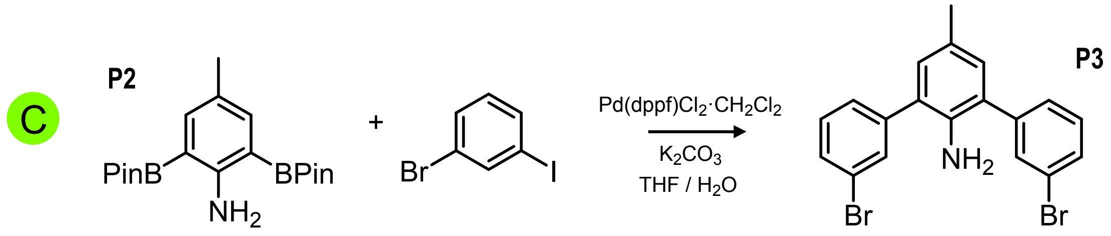

A 100 mL round bottom flask was charged with **P2** (1.00 g, 2.79 mmol, 50 *eqv*., M_r: 359), 1-bromo-3-iodobenzene (2.36 g, 8.36 mmol, 150 *eqv*., M_r: 238) and K_2CO_3 (2.31 g, 16.7 mmol, 300 *eqv.,* M_r: 138). The solids were suspended in THF (40 mL) and H_2O (10 mL) and deoxygenated with bubbling N_2 for 30 minutes, before addition of Pd(dppf)Cl_2·CH_2Cl_2 (45 mg, 0.06 mmol, 1 *eqv*., M_r: 816) and a further 10 minutes degassing. The mixture was heated for 18 hours at 65°C.

After cooling to room temperature, the suspension was poured into a separation funnel and ethyl acetate (50 mL) and water (50 mL) added. The organic layer was extracted, washed with water (50 mL) and then brine (50 mL). The organic layer was dried over anhydrous Na_2SO_4, and filtrate dry loaded onto Silica. Upon purification by column chromatography (25: 75 CH_2Cl_2: pentane (R_f = 0.6)) the product **P3** was afforded as a colourless oil (790 mg, 1.89 mmol, 68%, M_r: 417).

^{1}H NMR ($CDCl_3$, 298 K, 400 MHz): δ 7.68 (t., 2H, $^4J_{HH}$ = 1.8 Hz), 7.51 (d., 2H, $^3J_{HH}$ = 7.7 Hz), 7.45 (d., 2H, $^3J_{HH}$ = 7.7 Hz), 7.33 (t., 2H, $^3J_{HH}$ = 7.9 Hz), 7.51 (s., 2H), 3.68 (s., 2H), 2.32 (s., 3H).

^{13}C{^{1}H} NMR (**$CDCl_3$, 298 K, 101 MHz**): δ 141.9, 138.2, 132.4, 130.8, 130.4, 130.4, 128.0, 127.6, 126.8, 123.0, 20.4.

ESI-MS (calc. for [$C_{19}H_{15}Br_2N$-H]$^+$): *m/z* 417.9635 (417.9624).

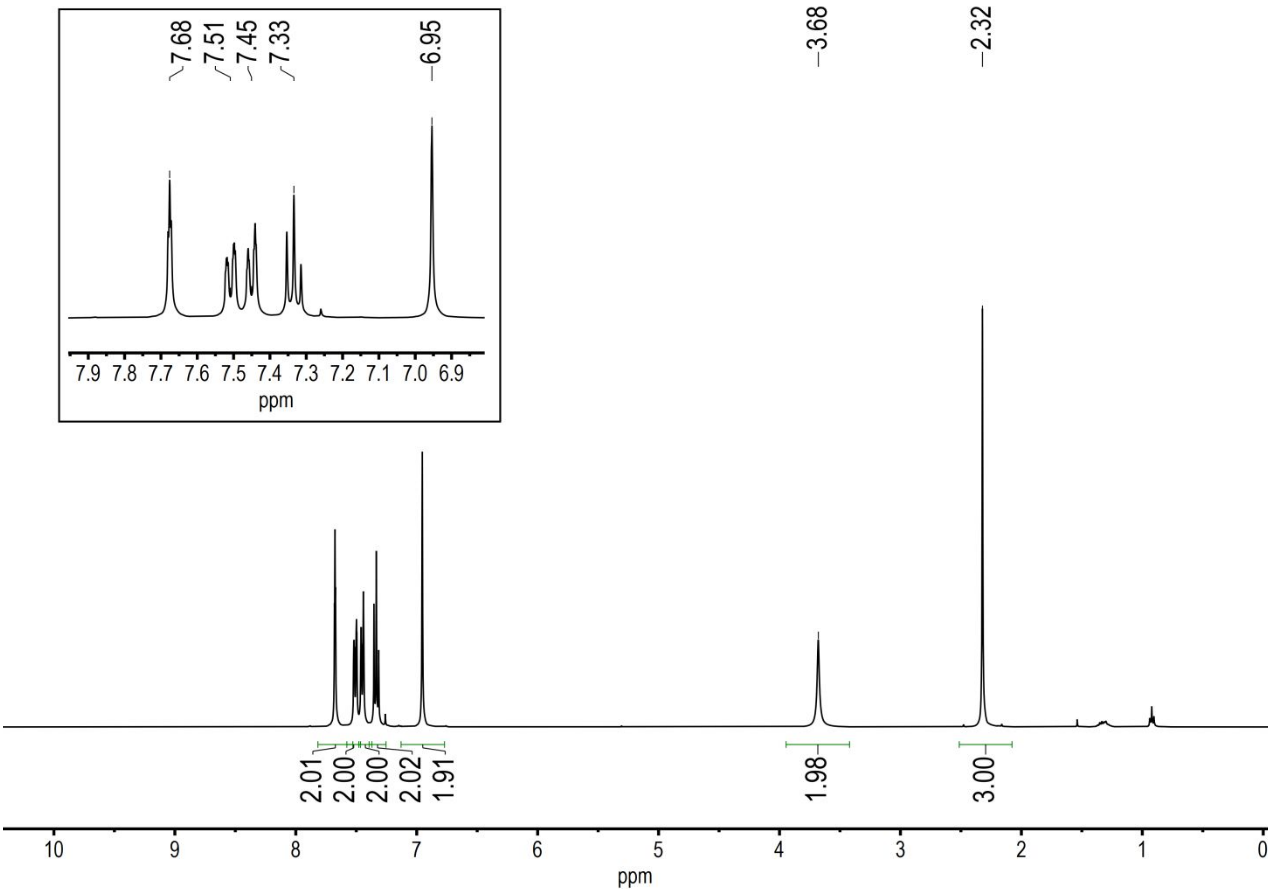

Figure S1: The solution ^{1}H NMR spectrum ($CDCl_3$, 298 K, 400 MHz) of **P3**.

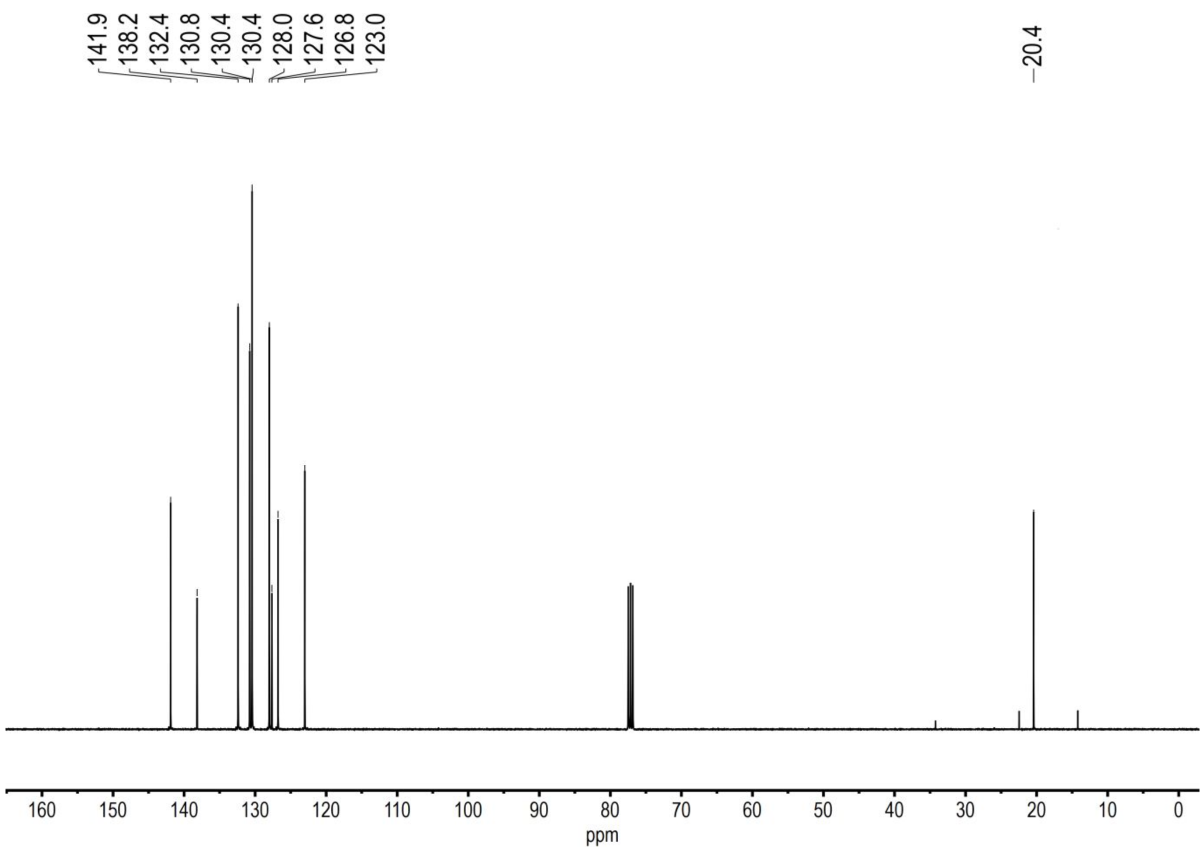

Figure S2: The solution $^{13}C\{^{1}H\}$ NMR spectrum ($CDCl_3$, 298 K, 101 MHz) of **P3**.

S.2.1.4. Step D

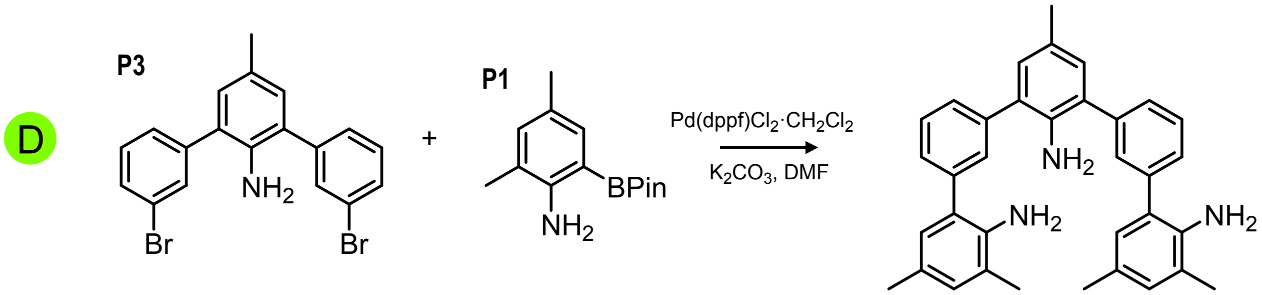

P4

A 50 mL round bottom flask was charged with **P3** (750 mg, 1.80 mmol, 25 *eqv.*, M_r: 417), **P1** (1.33 g, 5.40 mmol, 75 *eqv.*, M_r: 247) and K_2CO_3 (1.49 g, 10.8 mmol, 150 *eqv.,* M_r: 138). The solids were suspended in dry DMF (30 mL), the solution was deoxygenated with bubbling N_2 for 30 minutes before addition of Pd(dppf)Cl_2·CH_2Cl_2 (59 mg, 0.07 mmol, 1 *eqv.*, M_r: 816) and a further 10 minutes degassing. The mixture was heated for 2 hours at 110°C.

After cooling to room temperature, the suspension was poured into ice-cooled water (~150 mL) and grey precipitate collected. The solids were washed with further water (3 × 30 mL), before being extracted into dichloromethane (3 × 30 mL). The organic layers were combined, washed with brine (50 mL) and dried over anhydrous Na_2SO_4. The solution was filtered and filtrate loaded onto Silica. Upon purification by column chromatography (30: 70 ethyl acetate: pentane (R_f = 0.6)) **P4** was afforded as an off-white solid (720 mg, 1.45 mmol, 80%, M_r: 498).

^{1}H NMR ($CDCl_3$, 298 K, 400 MHz): δ 7.62 (s., 2H), 7.51 (m., 6H), 7.02 (s., 2H), 6.92 (m., 4H), 3.86 (s., 2H), 3.67 (s., 4H), 2.32 (s., 3H), 2.28 (s., 6H), 2.23 (s., 6H).

^{13}C{^{1}H} NMR (**$CDCl_3$, 298 K, 101 MHz**): δ 140.8, 140.6, 139.2, 138.4, 130.7, 130.6, 130.3, 129.3, 128.9, 128.1, 128.0, 128.0, 127.5, 127.4, 127.4, 122.9, 20.5, 20.5, 18.0.

ESI-MS (calc. for $[C_{35}H_{35}N_3\text{-}H]^+$): *m/z* 498.2900 (498.2904).

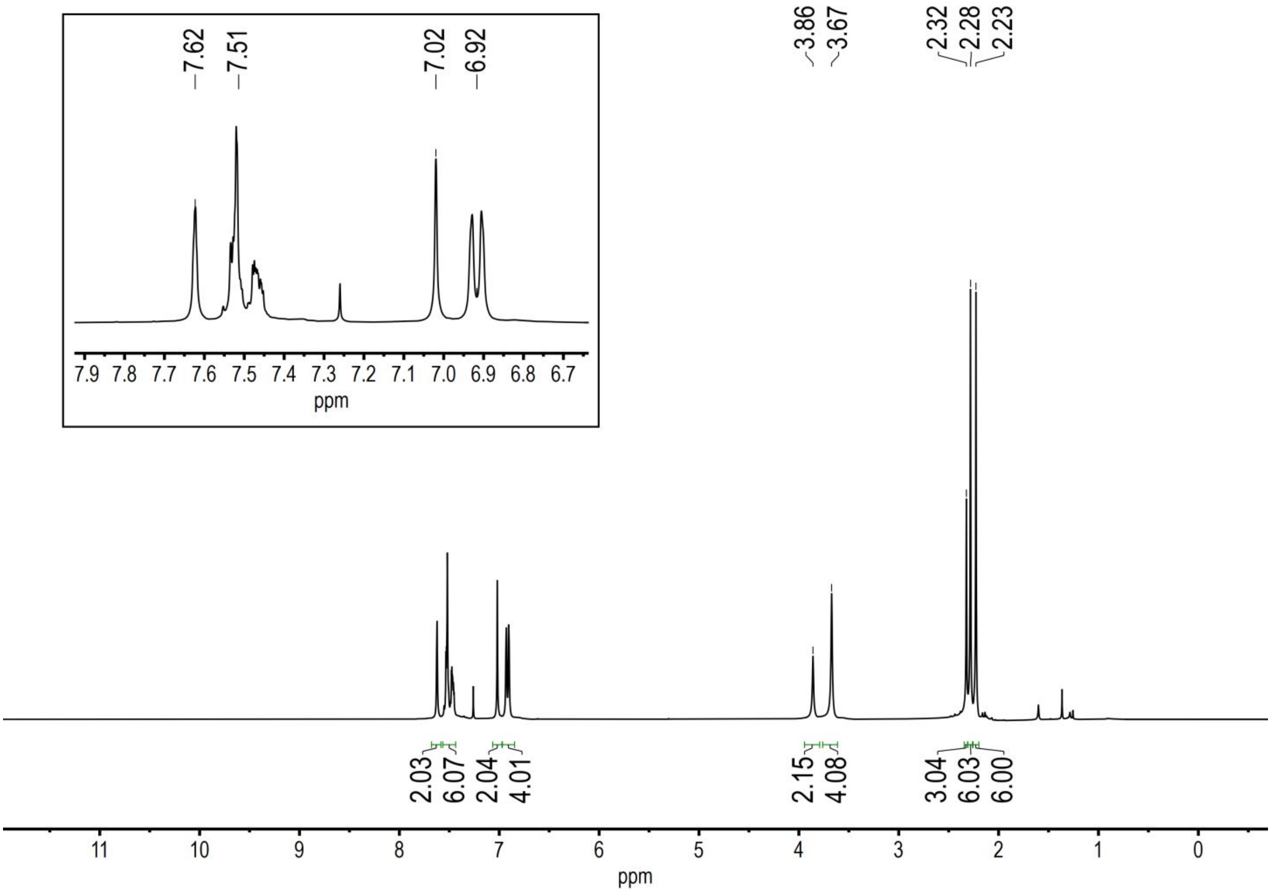

Figure S3: The solution ^{1}H NMR spectrum ($CDCl_3$, 298 K, 400 MHz) of **P4**.

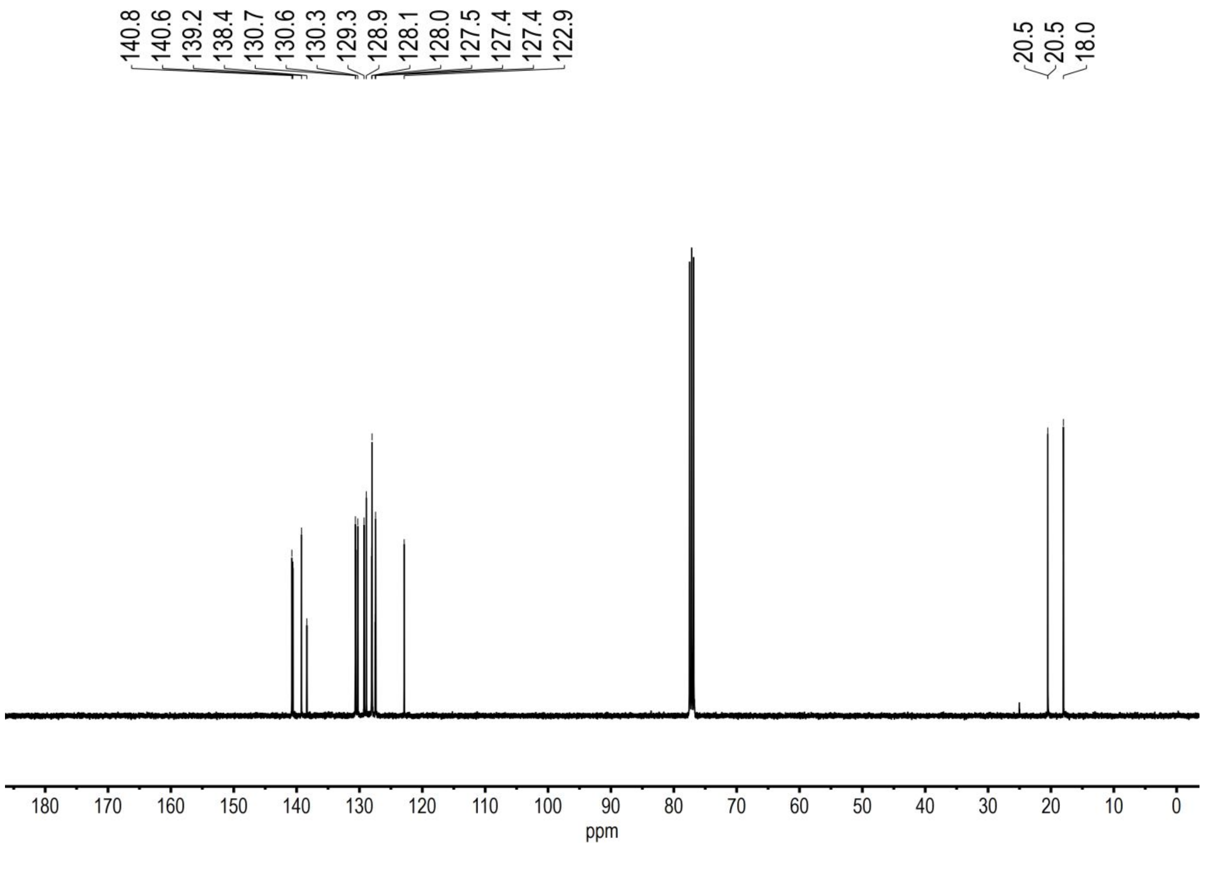

Figure S4: The solution $^{13}C\{^{1}H\}$ NMR spectrum ($CDCl_3$, 298 K, 101 MHz) of **P4**.

S.2.1.5. Step E

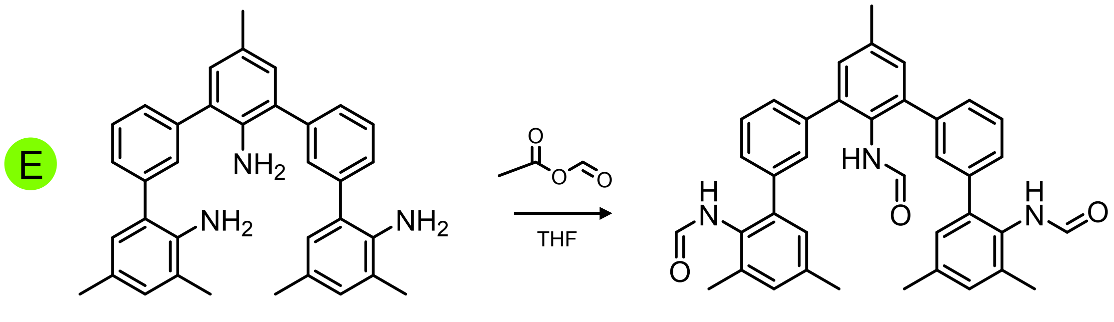

P4 **P5**

A 50 mL round bottom flask was charged with **P4** (700 mg, 1.41 mmol, M_r: 498) and dissolved in THF (30 mL). Freshly synthesised acetic formic anhydride (6.3 mL) was added and reaction left to stir for 16 hours at room temperature.

After this time, the now homogeneous solution was poured into ice-cooled distilled water (~150 mL) and left to stir until a permanent precipitate formed. The precipitate was filtered, washed with water (3 × 50 mL) then pentane (3 × 10 mL). The product was extracted into ethyl acetate (3 × 25 mL), dried over anhydrous Na_2SO_4 and solvent removed to yield **P5** as an off-white solid (700 mg, 1.20 mmol, 86%, M_r: 582).

Note: Acetic formic anhydride was synthesised by slow addition of formic acid (1.5 mL) to acetic anhydride (3 mL) at 0°C followed by deoxygenating with bubbling N_2 for 30 minutes. The mixture was heated for 50°C for 2 hours, cooled and opened to air to use as a neat reagent. A ratio of 1.5 mL acetic formic anhydride: 1 mmol $–NH_2$ was used.

^{1}H NMR (DMSO-*d*$_6$, 298 K, 500 MHz): δ 9.6 – 9.1 (br. m., 3H), 8.2 – 6.8 (br. m., 17H), 2.4 – 2.0 (m., 15H).

ESI-MS (calc. for $[C_{38}H_{35}N_3O_3\text{-}H]^+$): *m/z* 582.2759 (582.2751).

Note: Due to the rotameric effects and zwitterion forms of the formamide group, the full characterisation could not be deciphered through spectroscopy methodologies. This is consistent with previous reported systems by Bilger *et. al.* (*J. Am. Chem. Soc.*, **2021**, *143*, 1651)[S6] and Herr *et. al.* (*Nat. Chem.*, **2021**, *13,* 956).[S7]

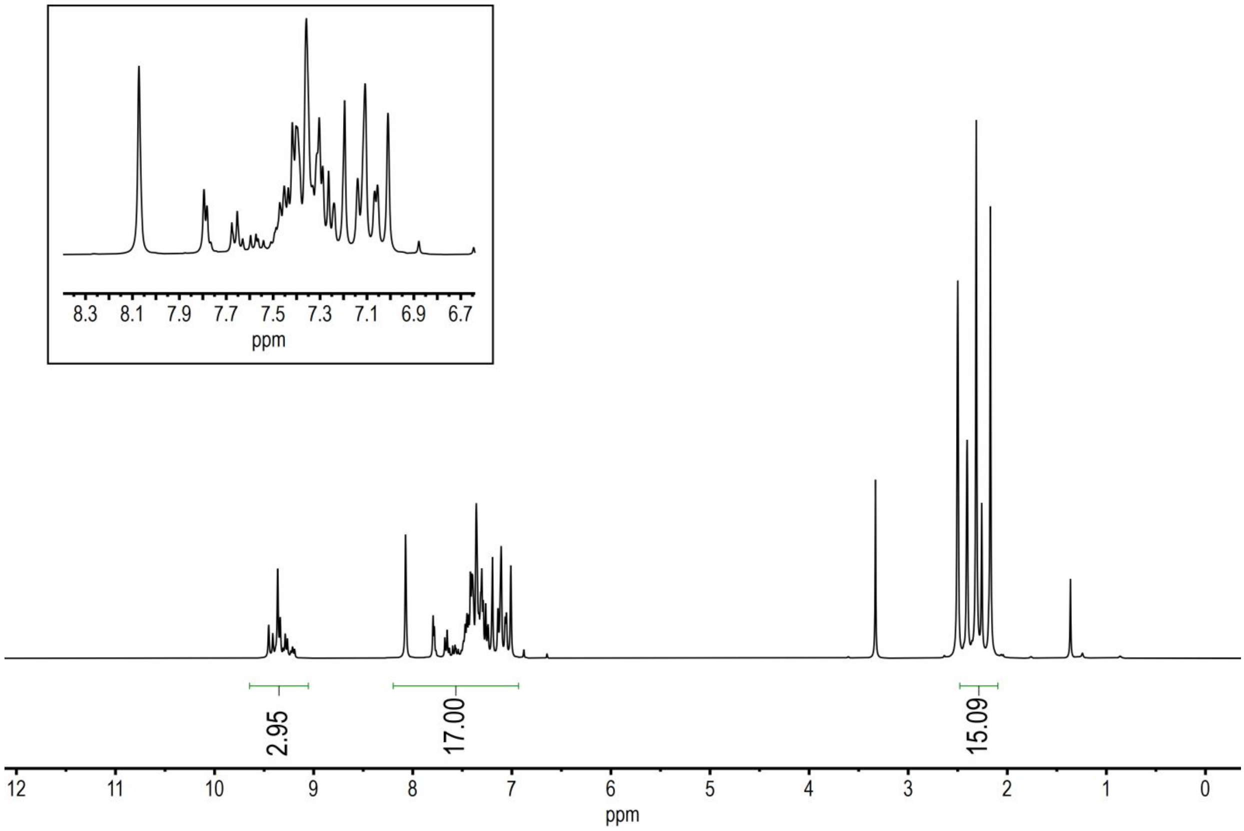

Figure S5: The solution ^{1}H NMR spectrum (DMSO-d_6, 298 K, 500 MHz) of **P5**. The inset is an enlargement of the resonances between δ 8.3 and 6.7.

S.3.1.6. Step F

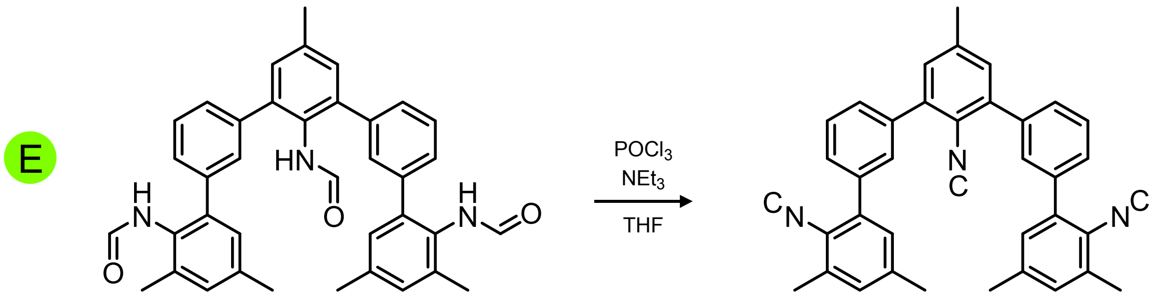

A 25 mL round bottom flask was charged with **P5** (700 mg, 1.20 mmol, 1 *eqv.*, M_r: 582) and placed under a nitrogen atmosphere. Dry THF (20 mL) and triethylamine (4.02 mL, 28.9 mmol, 24 *eqv.*) were added and suspension cooled to 0°C. $POCl_3$ (1.68 mL, 18.0 mmol, 15 *eqv.*) was added dropwise over 5 minutes before being left to warm to room temperature and stir for 16 hours.

After this time, and working under air, the pale-orange suspension was added dropwise over 10 minutes to an ice-cooled solution of saturated Na_2CO_3 solution (20 mL) and water (20 mL). The reaction was left to quench for 30 minutes at this temperature. The off-white precipitate was then filtered, washed with water (3 × 50 mL) and extracted into dichloromethane. The solution was dried over anhydrous Na_2SO_4, filtered and dry loaded onto Silica. Purification by column chromatography (90: 10 CH_2Cl_2: pentane (R_f = 0.8)) yielded ***meta*-Me** as a white solid (540 mg, 1.02 mmol, 85%, M_r: 528).

^{1}H NMR (CD_2Cl_2, 298 K, 500 MHz): δ 7.67 (m., 2H, **H_e**), 7.63 (m., 6H, **H_b**, **H_c**, **H_d**), 7.35 (s., 2H, **H_a**), 7.17 (s, 2H, **H_f**), 7.14 (s, 2H, **H_g**), 2.47 (2 × s., 3H & 6H, **H_h** and **H_i**), 2.38 (s., 6H, **H_j**).

^{13}C{^{1}H} NMR (**CD_2Cl_2, 298 K, 126 MHz**): δ 169.4 (**C_r**), 168.6 (**C_q**), 140.0 (**C_a**), 139.5 (**C_m**), 139.3 (**C_o**), 138.1, 138.0, 138.0, 135.7 (**C_k**), 130.5 (**C_b**), 130.2 (**C_n**), 129.9, 128.8, 128.8, 128.7, 128.5 (**C_l**), 122.2 (**C_p**), 120.3 (**C_d**), 21.1 (**C_u**), 21.0 (**C_t**), 19.0 (**C_s**).

Selected NOESY ^{1}H NMR (CD_2Cl_2, 298 K, 500 MHz) peaks: Cross-peak between δ 7.35 with 2.47, 7.17 with 2.38, 7.14 with 2.47 & 2.38.

ESI-MS (calc. for $[C_{38}H_{29}N_3\text{-Na}]^+$): *m/z* 550.2253 (550.2254).

Elemental Analysis (calc. for $C_{38}H_{29}N_3$): C 86.31 (86.50), H 5.54 (5.55), N 7.88 (7.96).

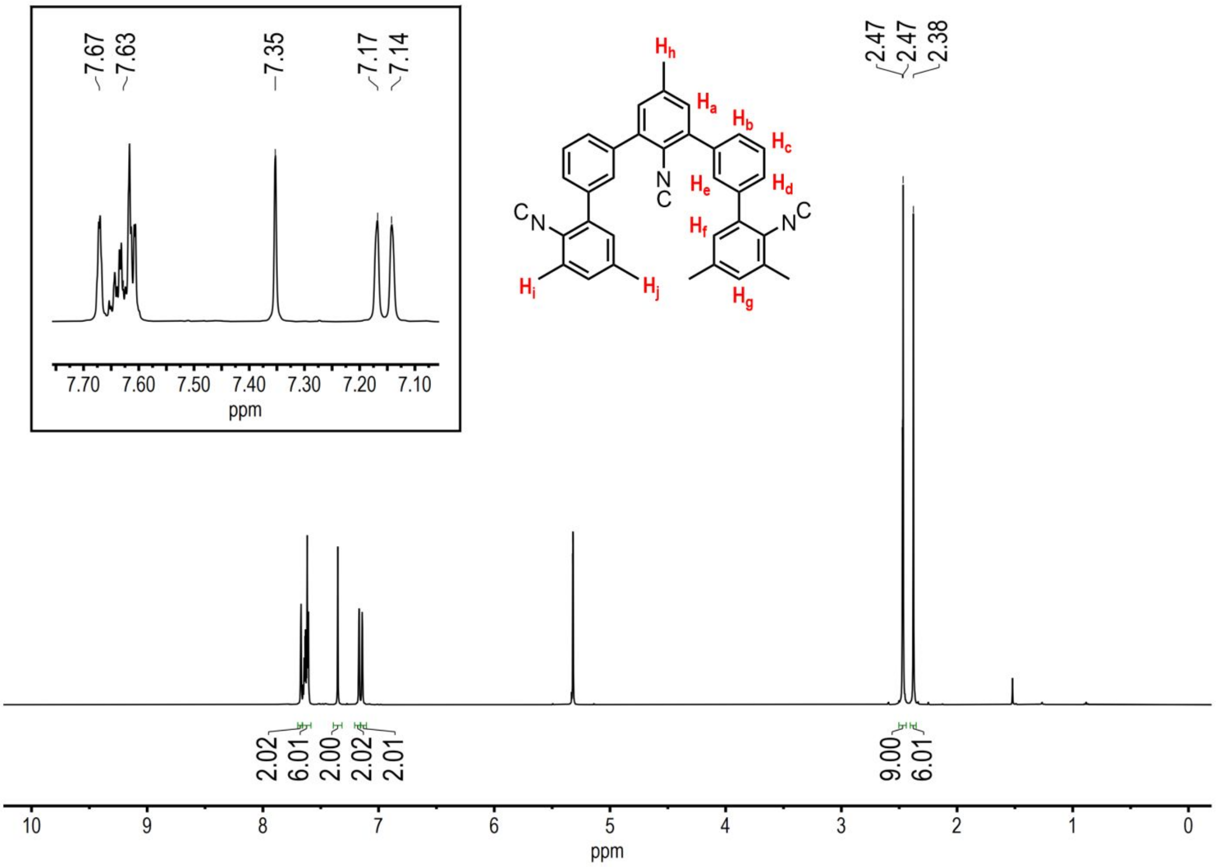

Figure S6: The solution ^{1}H NMR spectrum (CD_2Cl_2, 298 K, 500 MHz) of ***meta*-Me**. The inset is an enlargement of the resonances between δ 7.7 and 7.1.

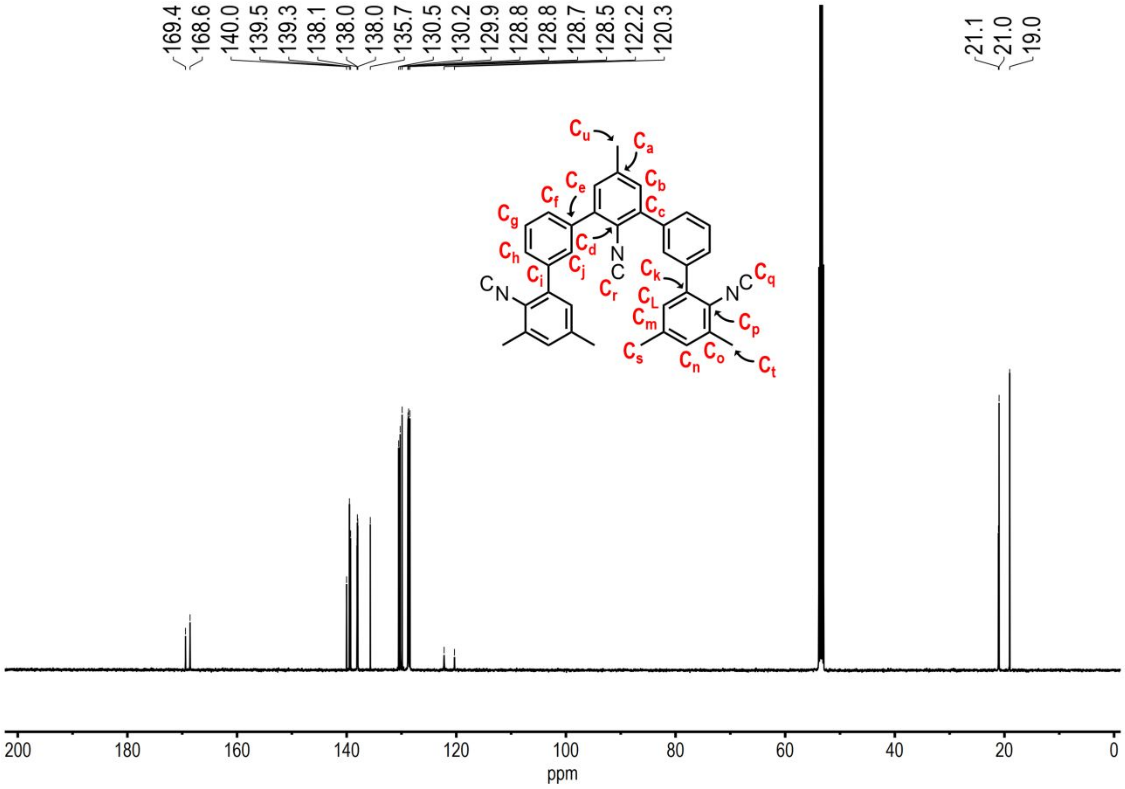

Figure S7: The solution ^{13}C{^{1}H} NMR spectrum (CD_2Cl_2, 298 K, 126 MHz) of ***meta*-Me**.

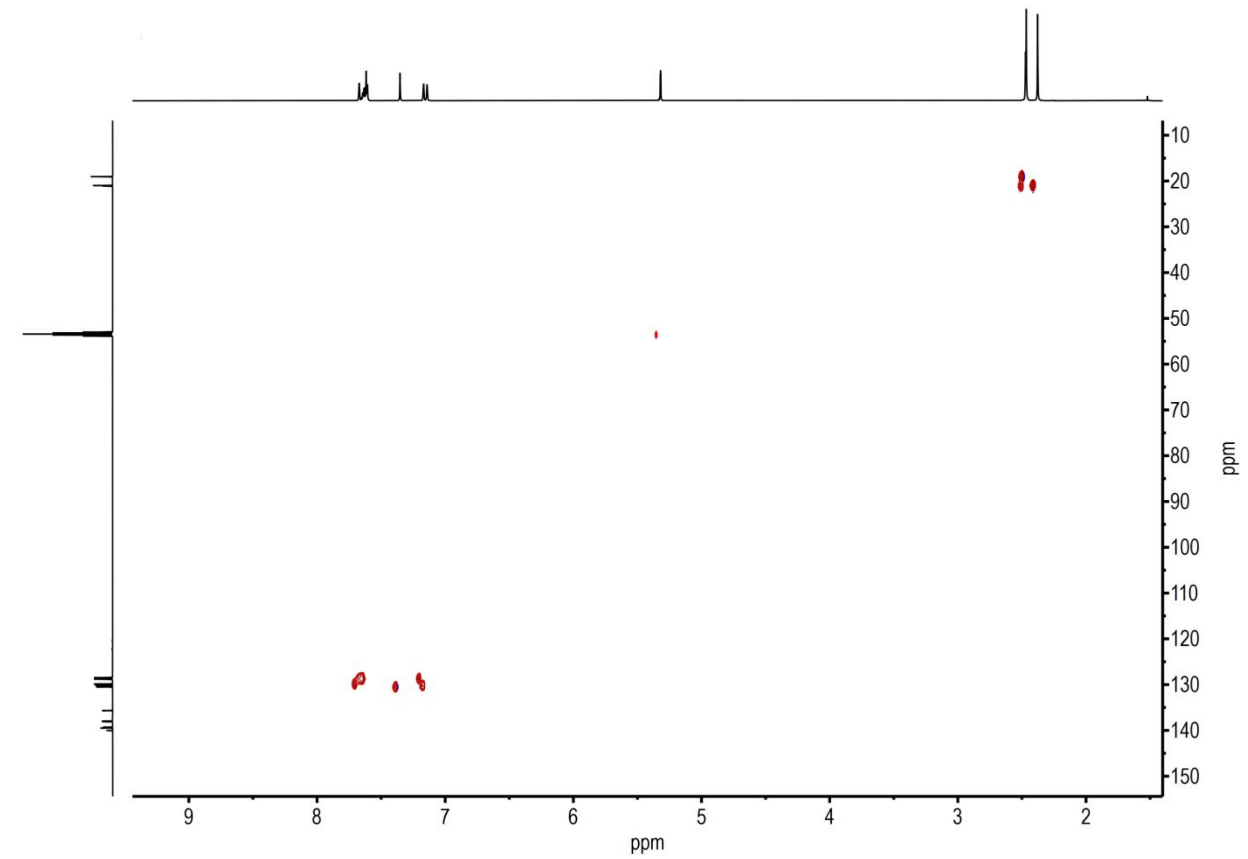

Figure S8: The solution ^{1}H-^{13}C HMQC spectrum (CD_2Cl_2, 298 K) of ***meta*-Me**.

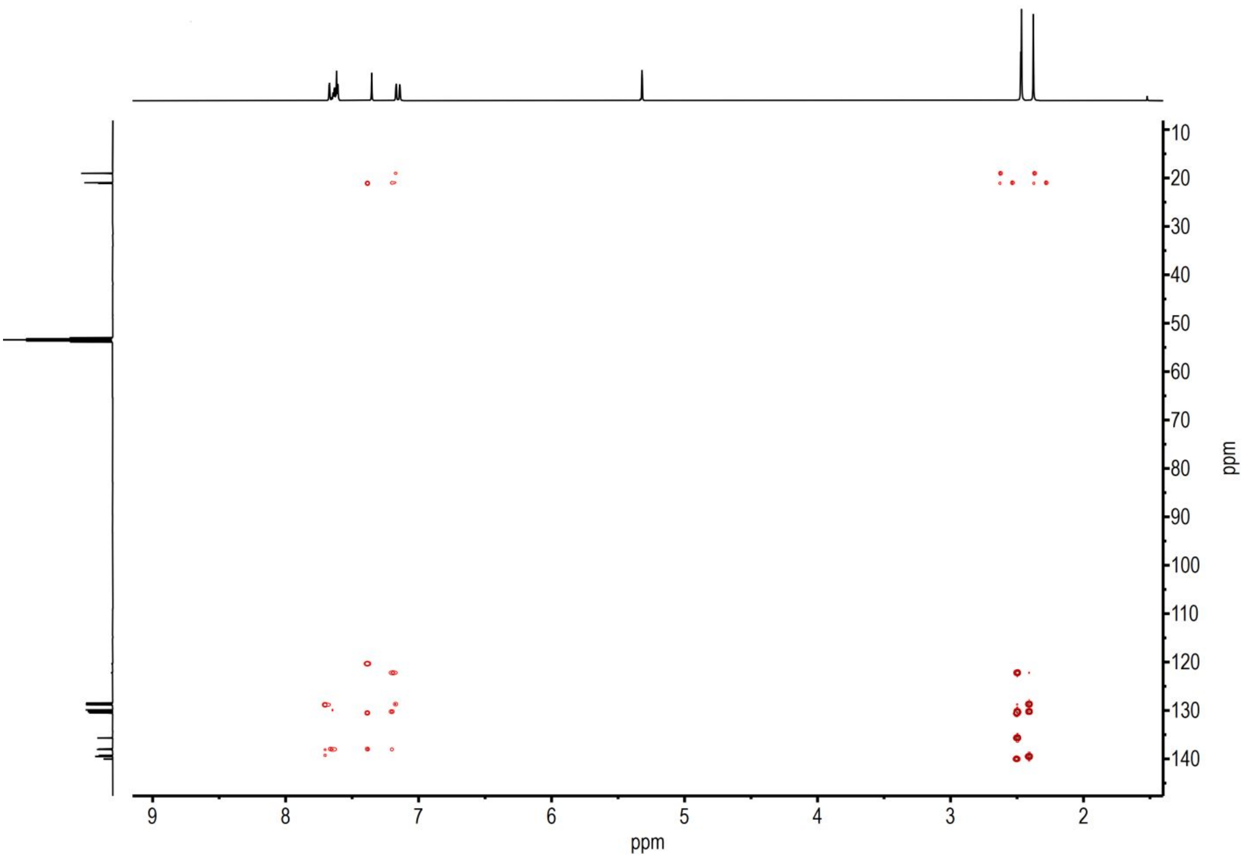

Figure S9: The solution ^{1}H-^{13}C HMBC spectrum (CD_2Cl_2, 298 K) of ***meta*-Me**.

S.2.2. *para*-Me

Procedure Overview:

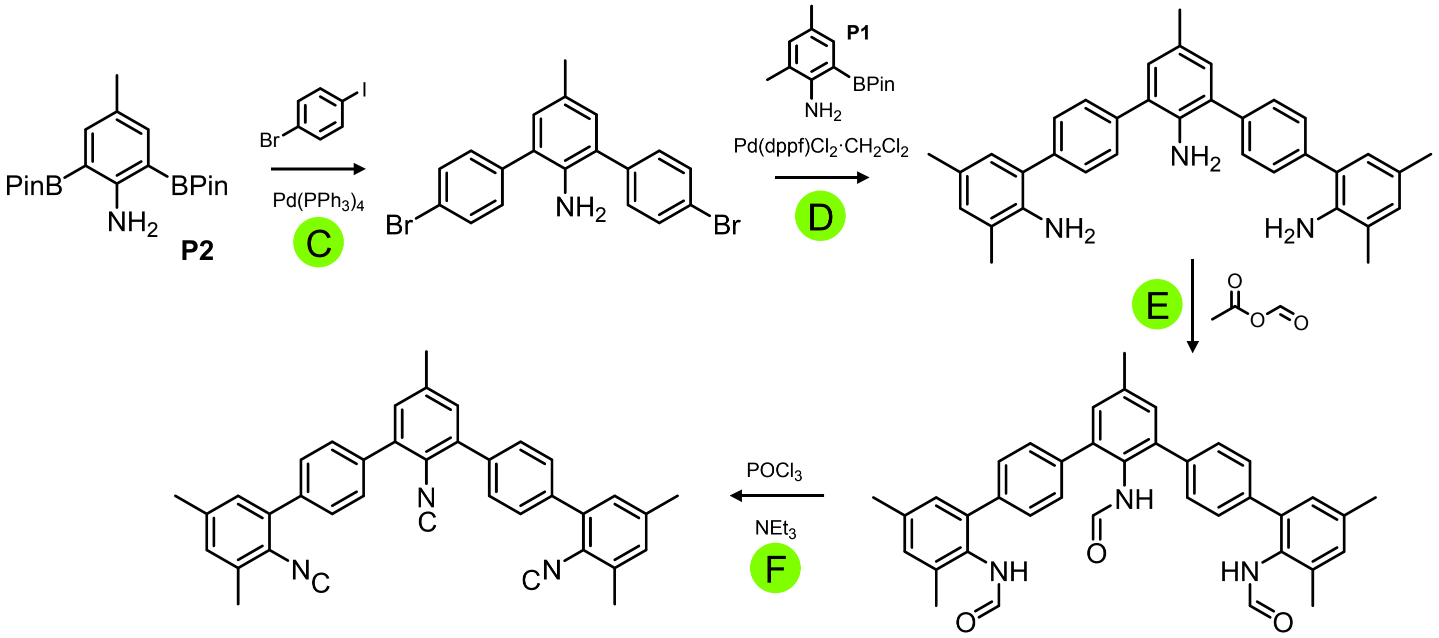

***para*-Me**

Note: Preparation for **P2** and **P1** are given in Section S.2.1., Steps A & B. Step C has been reported by Hu *et. al*. (*Angew. Chem. Int. Ed.*, **2022**, *61*, e2022073),[S8] however, has been adapted and improved in this preparation. Characterization data and spectra are given for all new products beneath each procedure.

S.2.2.3. Step C

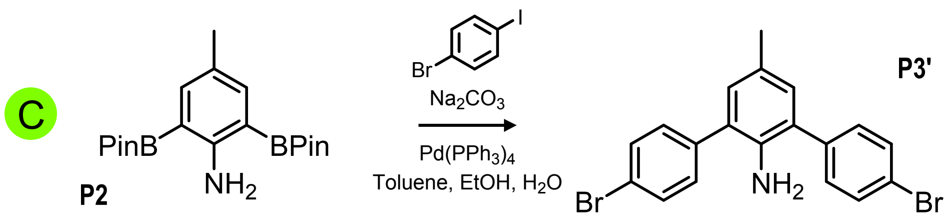

A 100 mL round bottom flask was charged with **P2** (1.50 g, 4.18 mmol, 25 *eqv.*, M_r: 359), 1-bromo-4-iodobenzene (3.55 g, 12.5 mmol, 75 *eqv.*, M_r: 283) and Na_2CO_3 (2.66 g, 25.1 mmol, 150 *eqv.*, M_r: 106). The solids were suspended in toluene (40 mL), ethanol (10 mL) and H_2O (10 mL). The solution was deoxygenated with bubbling N_2 for 30 minutes, before addition of $Pd(PPh_3)_4$ (193 mg, 0.17 mmol, 1 *eqv.,* M_r: 1156) and a further 10 minutes degassing. The mixture was heated for 18 hours at 90°C.

After cooling to room temperature, the suspension was poured into a separation funnel and ethyl acetate (50 mL) and water (50 mL) added. The organic layers were extracted and water layer washed with further ethyl acetate (2 × 20 mL). The organic layers were combined, dried over anhydrous Na_2SO_4, solvent removed under vacuum. The solids were re-dissolved in minimum dichloromethane and passed through a short Silica plug. The now bright yellow solution was again removed to a minimum (~5 mL) and methanol added (~40 mL). The resulting precipitate was filtered, washed with methanol (2 × 10 mL) then dried under reduced pressure to yield **P3'**. The methanol washings were combined, concentrated under reduced pressure and left to stand at 5°C for 24 hours. After this time, the crystalline material was filtered, washed rapidly with cooled (~5°C) methanol (5 mL) to yield **P3'** as a white solid (total: 1.45 g, 3.48 mmol, 83%, M_r: 417).

^{1}H NMR ($CDCl_3$, 298 K, 400 MHz): δ 7.57 (d, 4H, $^3J_{HH}$ = 8.4 Hz), 7.37 (d, 4H, $^3J_{HH}$ = 8.4 Hz), 6.92 (d, 2H), 3.62 (br. s, 2H), 2.29 (s, 3H).

S.2.2.4. Step D

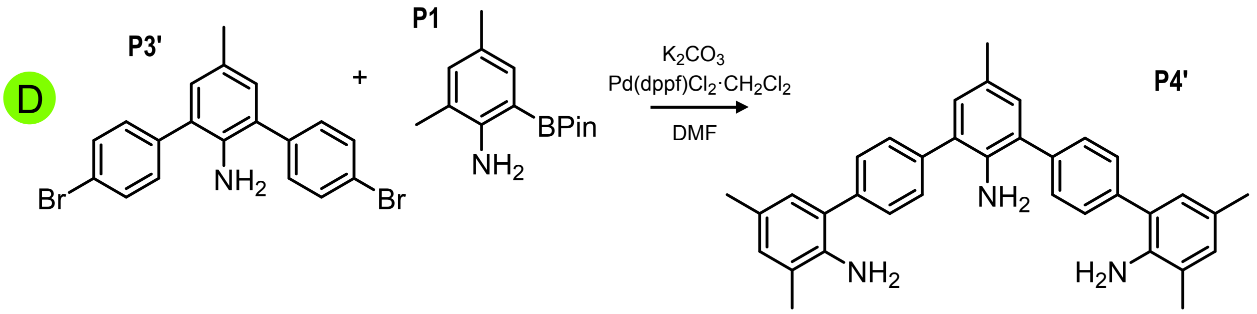

A 50 mL round bottom flask was charged with **P3'** (1.50 g, 3.60 mmol, 25 *eqv.*, M_r: 417), **P1** (2.67 g, 10.8 mmol, 75 *eqv.*, M_r: 247) and K_2CO_3 (2.98 g, 21.6 mmol, 150 *eqv.,* M_r: 138). The solids were suspended in dry DMF (30 mL), solution was deoxygenated with bubbling N_2 for 30 minutes, before addition of Pd(dppf)Cl_2·CH_2Cl_2 (115 mg, 0.14 mmol, 1 *eqv.*, M_r: 816) and a further 10 minutes degassing. The mixture was heated for 2 hours at 110°C.

After cooling to room temperature, the suspension was poured into water (~150 mL) and grey precipitate collected. The solids were washed with further water (3 × 50 mL), before being extracted into dichloromethane (3 × 20 mL). The organic layers were combined, dried over anhydrous Na_2SO_4, solvent removed under vacuum. The solids were then re-dissolved in minimum dichloromethane and passed through a short Silica plug. The yellow solution was removed to a minimum (~5 mL) and methanol added (~40 mL). The resulting precipitate was filtered and used in the next step without any further purification.

Pure material can be obtained from column chromatography (20: 80 ethyl acetate: cyclohexane (R_f = 0.3)) after dry loading the crude extraction onto Silica. This yields **P4'** as an off-white solid (1.15 g, 2.31 mmol, 65%, M_r: 498).

^{1}H NMR ($CDCl_3$, 298 K, 400 MHz): δ 7.60 (d, 4H, $^3J_{HH}$ = 8.01 Hz), 7.55 (d, 4H, $^3J_{HH}$ = 8.01 Hz), 7.03 (s, 2H), 6.93 (br. s, 2H), 6.91 (br. s, 2H), 3.86 (br. s, 2H), 3.68 (br. s, 4H), 2.35 (s, 3H), 2.29 (s, 6H), 2.33 (s, 6H).

^{13}C{^{1}H} NMR (**$CDCl_3$, 298 K, 101 MHz**): δ 139.3, 139.0, 138.7, 138.5, 130.7, 130.6, 129.8, 129.8, 128.9, 128.0, 127.6, 127.5, 127.3, 122.9, 20.6, 20.5, 18.1.

ESI-MS (calc. for $[C_{35}H_{35}N_3\text{-H}]^+$): *m/z* 498.2903 (498.2904).

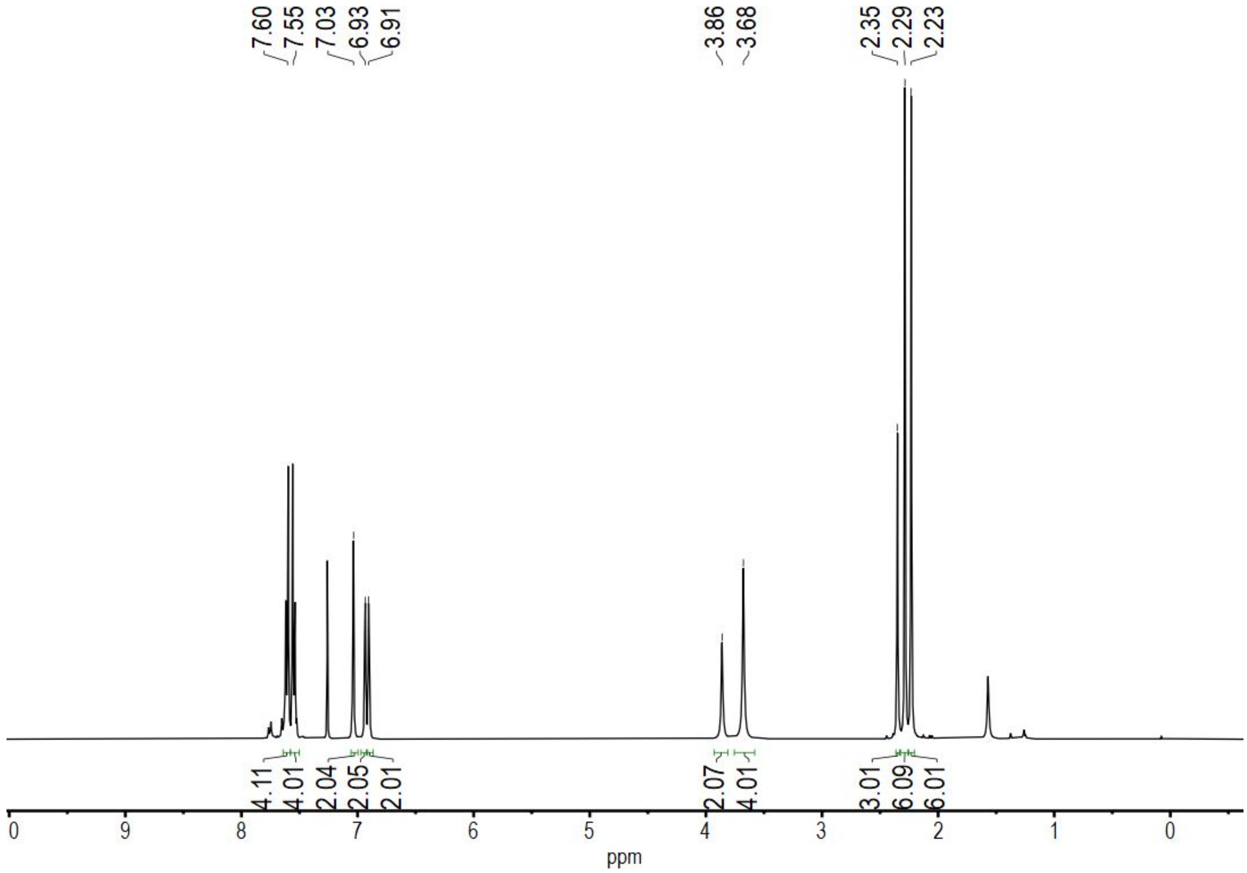

Figure S10: The solution ^{1}H NMR spectrum ($CDCl_3$, 298 K, 400 MHz) of **P4'**.

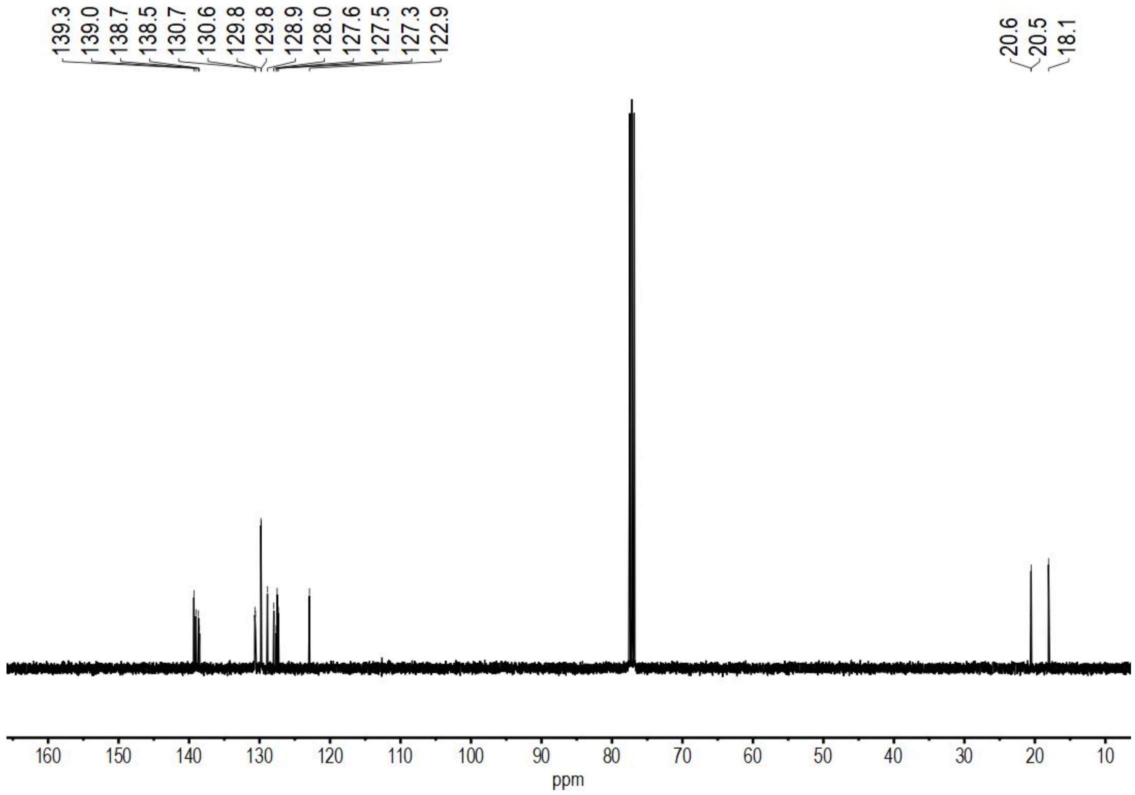

Figure S11: The solution $^{13}C\{^{1}H\}$ NMR spectrum ($CDCl_3$, 298 K, 101 MHz) of **P4'**.

S.2.2.5. Step E

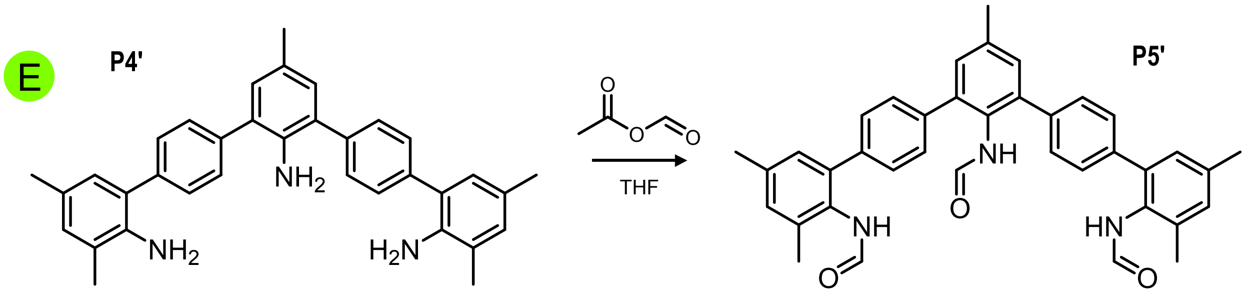

A 50 mL round bottom flask was charged with **P4'** (700 mg, 1.41 mmol, M_r: 498) and suspended in THF (30 mL). Freshly synthesised acetic formic anhydride (6.3 mL) was added and reaction left to stir for 16 hours at room temperature.

After this time, the reaction mixture was poured into ice-cooled distilled water (~150 mL) and left to stir until a permanent precipitate forms. The precipitate was filtered, washed with water (3 × 50 mL), dichloromethane (3 × 5 mL) and finally pentane (3 × 20 mL) before drying under reduced pressure to yield **P5'** as an off-white solid (580 mg, 1.00 mmol, 72%, M_r: 581).

Note: Acetic formic anhydride was synthesised by slow addition of formic acid (1.5 mL) to acetic anhydride (3 mL) at 0°C followed by deoxygenating with bubbling N_2 for 30 minutes. The mixture was heated for 50°C for 2 hours, cooled and opened to air to use as a neat reagent. A ratio of 1.5 mL acetic formic anhydride: 1 mmol $–NH_2$ was used.

^{1}H NMR (DMSO-*d*$_6$, 298 K, 500 MHz): δ 9.7 – 9.2 (br. m., 3H), 8.3 – 6.8 (br. m., 17H), 2.4 – 2.1 (m., 15H).

ESI-MS (calc. for $[C_{38}H_{35}N_3O_3\text{-}Na]^+$): *m/z* 604.2567 (604.2571).

Note: Due to the rotameric effects and zwitterion forms of the formamide group, the full characterisation could not be deciphered through spectroscopy methodologies. This is consistent with previous reported systems by Bilger *et. al.* (*J. Am. Chem. Soc.*, **2021**, *143*, 1651)[S6] and Herr *et. al.* (*Nat. Chem.*, **2021**, *13,* 956).[S7]

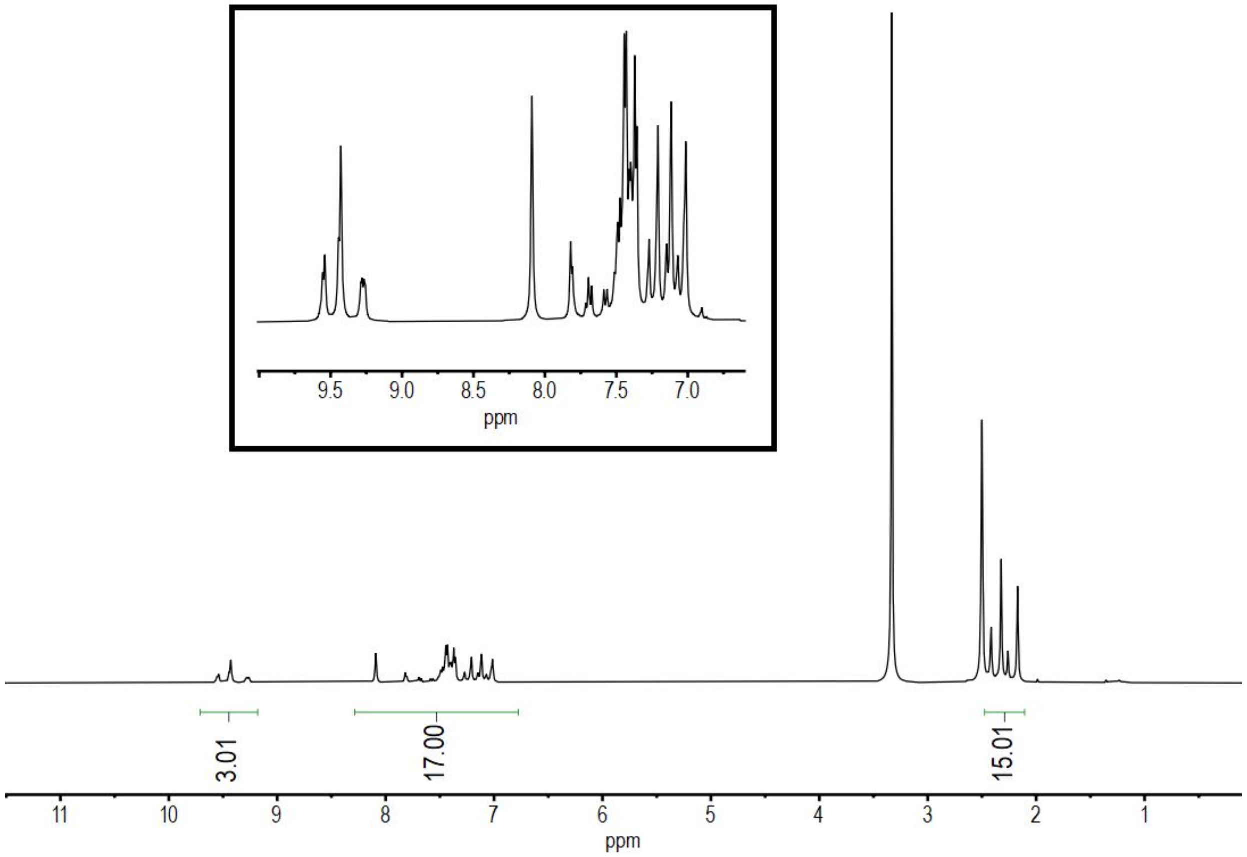

Figure S12: The solution ^{1}H NMR spectrum (DMSO-d_6, 298 K, 500 MHz) of **P5'**. The inset is an enlargement of the resonances between δ 10.0 and 6.5.

S.2.2.6. Step F

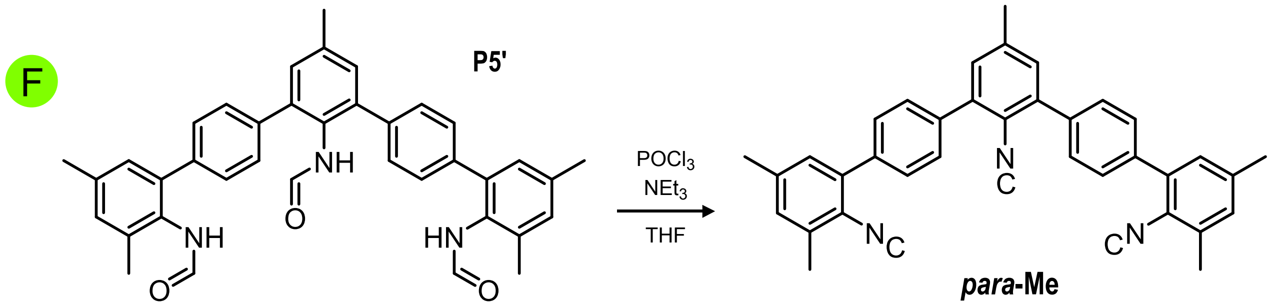

A 25 mL round bottom flask was charged with **P5’** (500 mg, 0.86 mmol, 1 *eqv.*, M_r: 581) and placed under a nitrogen atmosphere. Dry THF (20 mL) and triethylamine (2.87 mL, 20.65 mmol, 24 *eqv.*) were added and suspension cooled to 0°C. $POCl_3$ (1.20 mL, 12.91 mmol, 15 *eqv.*) was added dropwise over 5 minutes before being left to warm to room temperature and stir for 16 hours.

After this time, and working under air, the pale-orange suspension was added dropwise over 10 minutes to an ice-cooled solution of saturated Na_2CO_3 solution (20 mL) and water (20 mL). The reaction was left to quench for 30 minutes at this temperature. The off-white precipitate was then filtered, washed with water (3 × 50 mL) and extracted into dichloromethane. The solution was dried over anhydrous Na_2SO_4, filtered and dry loaded onto Silica. Purification by column chromatography (80: 20 CH_2Cl_2: pentane (R_f = 0.6)) yielded ***para*-Me** as a white solid (350 mg, 0.66 mmol, 77%, M_r: 528).

^{1}H NMR (CD_2Cl_2, 298 K, 400 MHz): δ 7.58 (m., 8H, **H_b** and **H_c**), 7.25 (s, 2H, **H_a**), 7.06 (m., 4H, **H_d** and **H_e**), 2.48 (s, 3H, **H_f** or **H_g**), 2.47 (s, 6H, **H_h**), 2.38 (s, 6H, **H_f** or **H_g**).

^{13}C{^{1}H} NMR (**CD_2Cl_2, 298 K, 101 MHz**): δ 169.8 (**C_r**), 168.9 (**C_q**), 140.3, 139.8, 139.5, 138.3, 138.2, 138.0, 136.1, 130.9 (**C_b**), 130.6 (**C_i** or **C_k**), 129.6 (**C_e** or **C_f**), 129.5 (**C_e** or **C_f**), 129.1 (**C_i** or **C_k**), 122.6, 120.7,f 21.5 (**C_p**), 21.4 (**C_n** or **C_o**), 19.5 (**C_n** or **C_o**).

ESI-MS (calc. for $[C_{38}H_{29}N_3\text{-}Na]^+$): *m/z* 550.2252 (550.2254).

Elemental Analysis (calc. for $C_{38}H_{29}N_3$): C 86.20 (86.50), H 5.44 (5.54), N 7.91 (7.96).

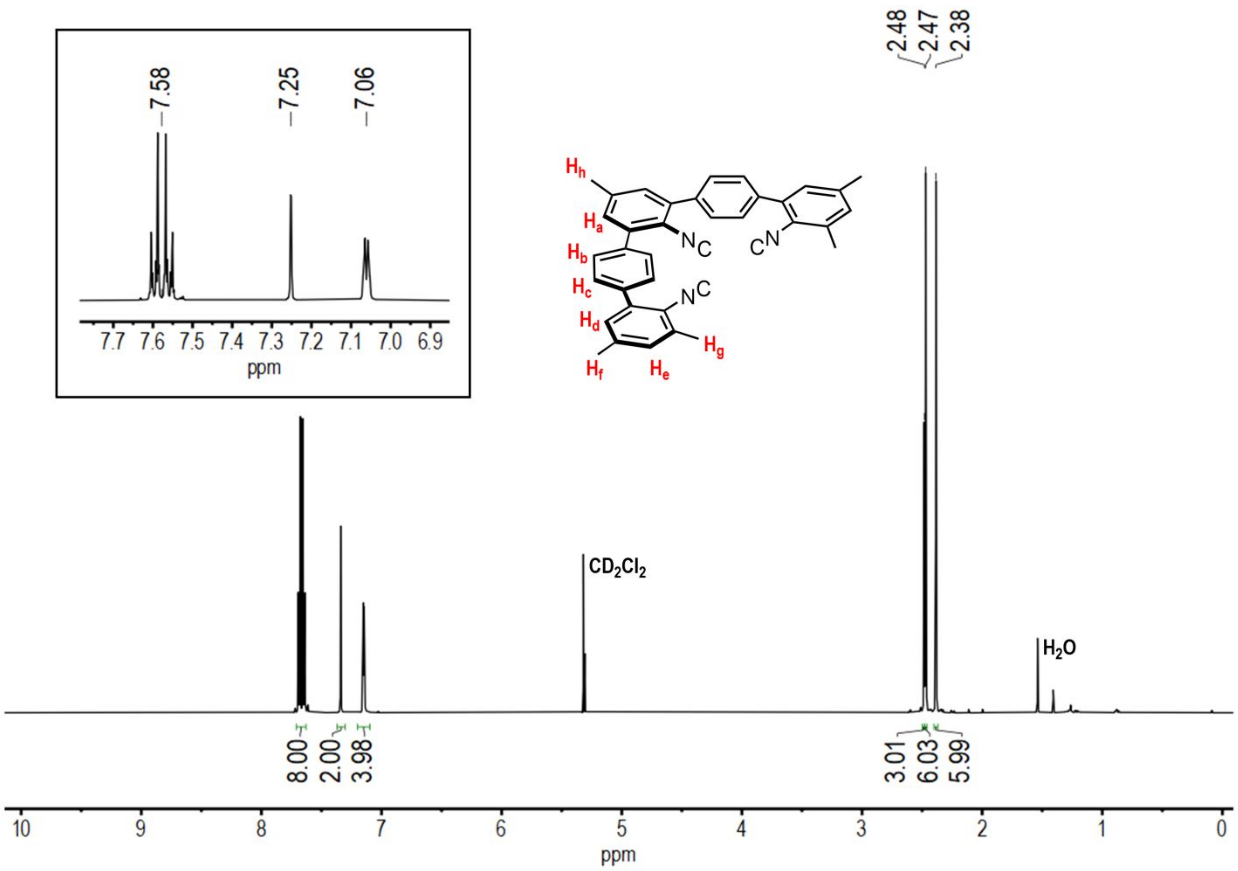

Figure S13: The solution ^{1}H NMR spectrum (CD_2Cl_2, 298 K, 400 MHz) of ***para*-Me**. The inset is an enlargement of the resonances between δ 7.8 and 6.8.

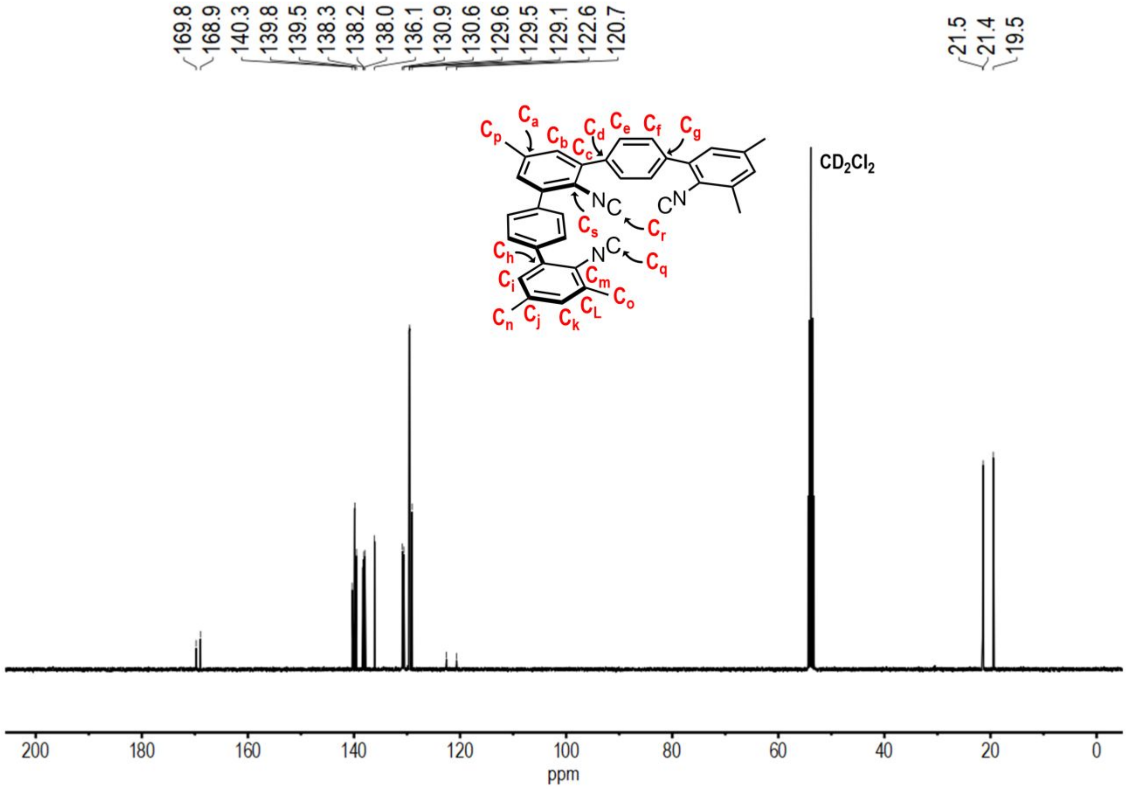

Figure S14: The solution ^{13}C{^{1}H} NMR spectrum (CD_2Cl_2, 298 K, 101 MHz) of ***para*-Me**.

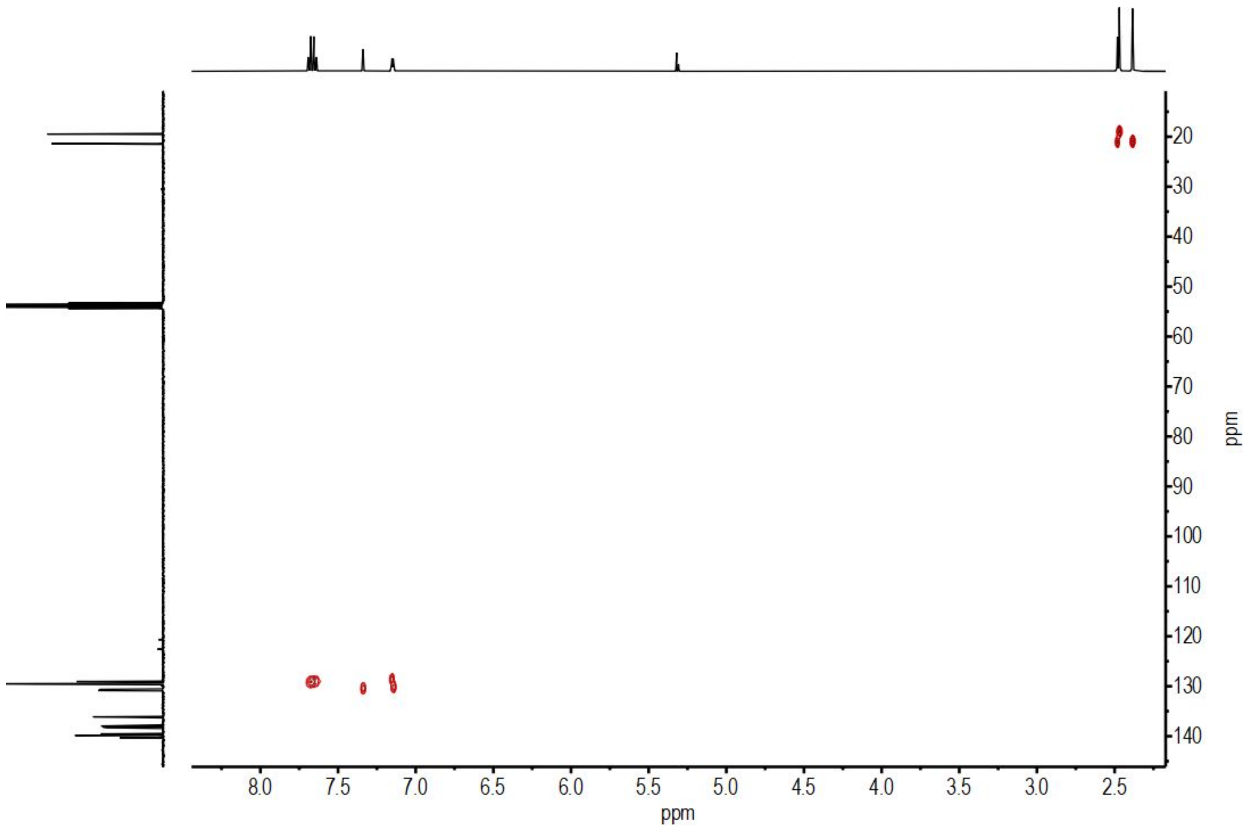

Figure S15: The solution ^{1}H-^{13}C HMQC spectrum (CD_2Cl_2, 298 K) of ***para*-Me**.

S.2.3. *para-*^{t}**Bu**

Procedure Overview:

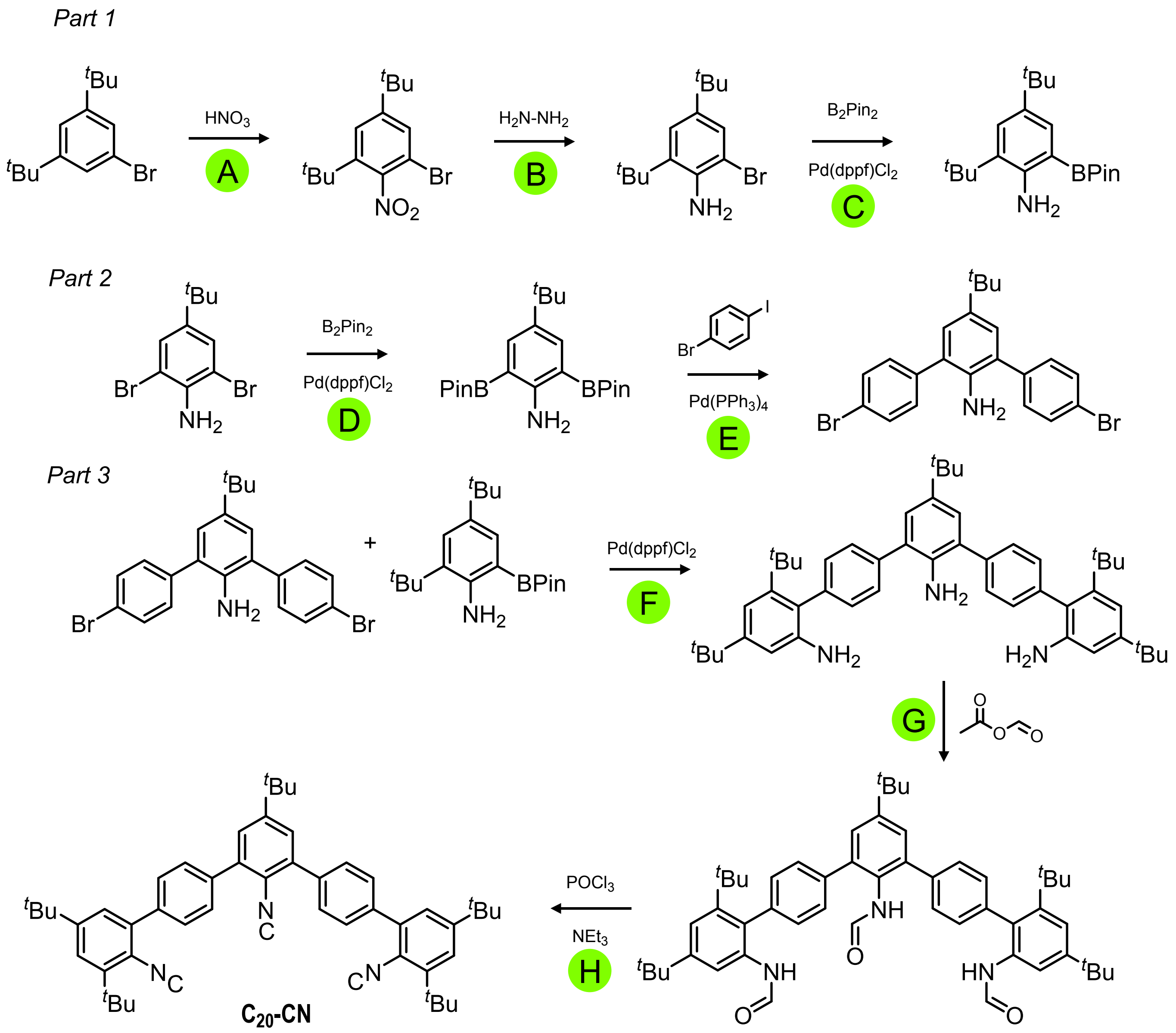

Note: Steps A and B have been reported by Herr *et. al*. (*Nat. Chem.*, **2021**, *13,* 956),[S7] however have been adapted or improved in this preparation. Characterization data and spectra are given for all new products beneath each procedure.

S.2.3.1. Step A

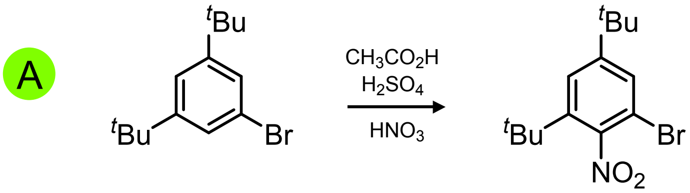

P1"

1-bromo-3,5-di-tert-butylbenzene (15 g, 55.7 mmol) was suspended in a mixture of glacial acetic acid (150 mL) and concentrated sulfuric acid (84 mL) at 50°C. 62-68% Nitric acid (14 mL) was added, and the solution was stirred at 80 °C for 90 minutes. The reaction mixture was poured into ice-cooled distilled water (~300 mL) and left to stir until a permanent precipitate formed. The solids were filtered, washed with water (3 × 20 mL) and dissolved in dichloromethane. This solution was dried over anhydrous Na_2SO_4 and solvent removed under reduced pressure. The resulting yellows solids were dissolved in minimal methanol (~15 mL) and left to stand at 5°C for 24 hours. After this time, the crystalline material was filtered, washed rapidly with cooled (~5°C) methanol (2 × 5 mL) and dried under reduced pressure to yield **P1"** as a white solid (14.6 g, 46 mmol, 83%).

^{1}H NMR ($CDCl_3$, 298 K, 400 MHz): δ 7.49 (d, 1H, $^4J_{HH}$ = 1.9 Hz), 7.48 (t, 1H, $^4J_{HH}$ = 1.9 Hz), 1.37 (s, 9H), 1.35 (s, 9H).

S.2.3.2. Step B

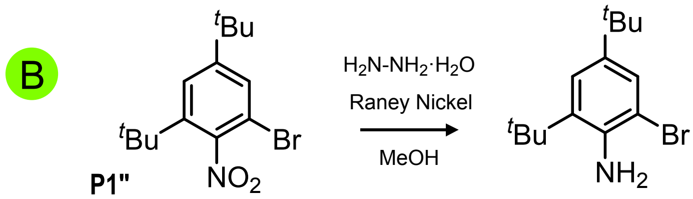

P2"

P1" (3.5 g, 11.2 mmol) and Raney Nickel (1.5 g) were suspended in MeOH (25 mL) and cooled to 0°C using an ice-bath. Hydrazine monohydrate (65% solution, 2 mL) was added over a period of 5 mins as effervesce began. The mixture was left for 2 hours to warm before addition of distilled water (~150 mL). The suspension was filtered through Celite and washed with water (3 × 20 mL). The organics were extracted from the Celite with dichloromethane (3 × 15 mL) and filtered through the filter frit. The solution was dried over anhydrous Na_2SO_4 and solvent removed under reduced pressure to yield **P2"** as a colourless oil (2.86 g, 91%).

^{1}H NMR ($CDCl_3$, 298 K, 400 MHz): δ 7.35 (d, 1H, $^4J_{HH}$ = 2.2 Hz), 7.23 (d, 1H, $^4J_{HH}$ = 2.2 Hz), 4.26 (br. s, 2H) 1.43 (s, 9H), 1.27 (s, 9H).

Note: **P2"** was used in Step C without any further purification but can be cleanly afforded through column chromatography (Silica, 90: 10 Pet. Ether: CH_2Cl_2 (R_f = 0.5)).

S.2.3.3. Step C

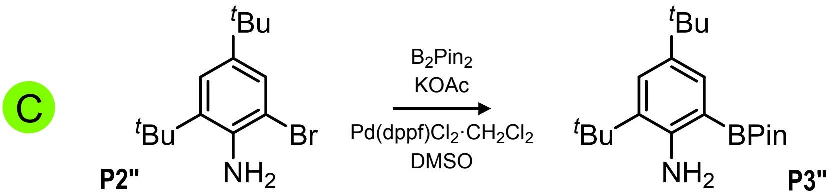

A 100 mL round bottom flask was charged with **P2"** (2.50 g, 8.80 mmol, 50 *eqv.* M_r: 284), bis(pinacolato)diboron (2.91 g, 11.4 mmol, 65 *eqv.* M_r: 254) and KOAc (2.59 g, 26.4 mmol, 150 *eqv.,* M_r: 98). Dry DMSO (50 mL) was added and solution deoxygenated with bubbling N_2 for 30 minutes, before addition of Pd(dppf)Cl_2·CH_2Cl_2 (144 mg, 0.18 mmol, 1 *eqv.,* M_r: 816) and a further 10 minutes degassing. The mixture was heated for 18 hours at 80°C.

After cooling to room temperature, the reaction mixture was poured into ice-cooled distilled water (~200 mL) and left to stir until a permanent precipitate formed. The brown solids were filtered, washed with water (3 × 20 mL) and dissolved in dichloromethane. This solution was dried over anhydrous Na_2SO_4, solvent removed to a minimum (~5 mL) under reduced pressure and passed through a short Silica plug. The now pale green solution was again removed to a minimum (~5 mL), methanol added (~20 mL) and left to stand at 5°C for 24 hours. After this time, the crystalline material was filtered, washed rapidly with cooled (~5°C) methanol (2 × 5 mL) and dried under reduced pressure to yield **P3"** as a green-white solid (1.49 g, 4.50 mmol, 51%, M_r: 331).

^{1}H NMR ($CDCl_3$, 298 K, 400 MHz): δ 7.57 (d, 1H, $^4J_{HH}$ = 2.5 Hz), 7.41 (d, 1H, $^4J_{HH}$ = 2.5 Hz), 5.03 (br. s, 2H), 1.45 (s, 9H), 1.35 (s, 12H), 1.31 (s, 9H).

^{13}C{^{1}H} NMR (**CD_2Cl_2, 298 K, 101 MHz**): δ 149.8, 138.7, 132.1, 131.2, 127.7, 83.4, 77.1, 34.5, 34.1, 31.6, 29.8, 24.9.

^{11}B{^{1}H} NMR (**$CDCl_3$, 298 K, 128 MHz**): δ 30.94.

ESI-MS (calc. for $[C_{20}H_{34}B_1N_1O_2\text{-}H]^+$): *m/z* 332.2761 (332.2755).

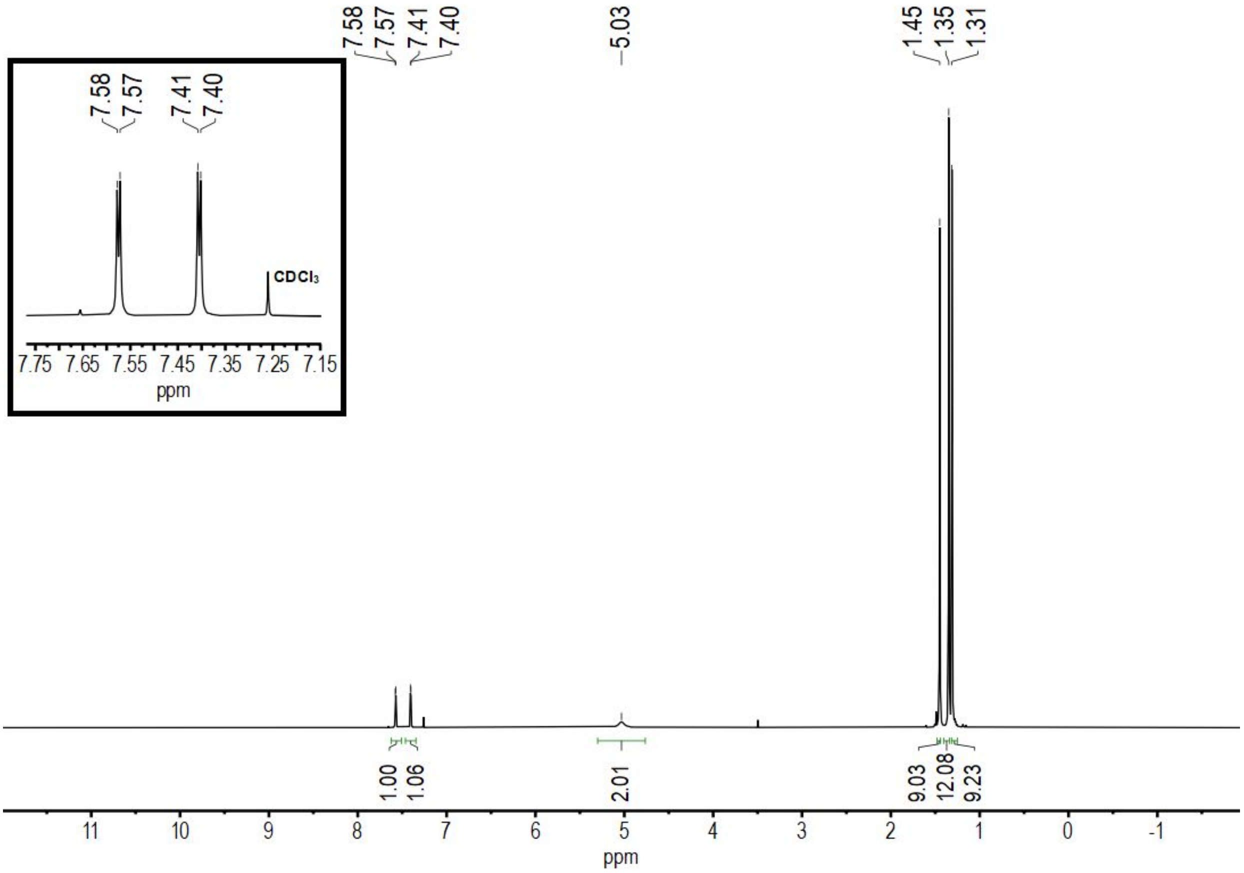

Figure S16: The solution ^{1}H NMR spectrum ($CDCl_3$, 298 K, 400 MHz) of **P3''** The inset is an enlargement of the resonances between δ 7.8 and 7.15 ppm.

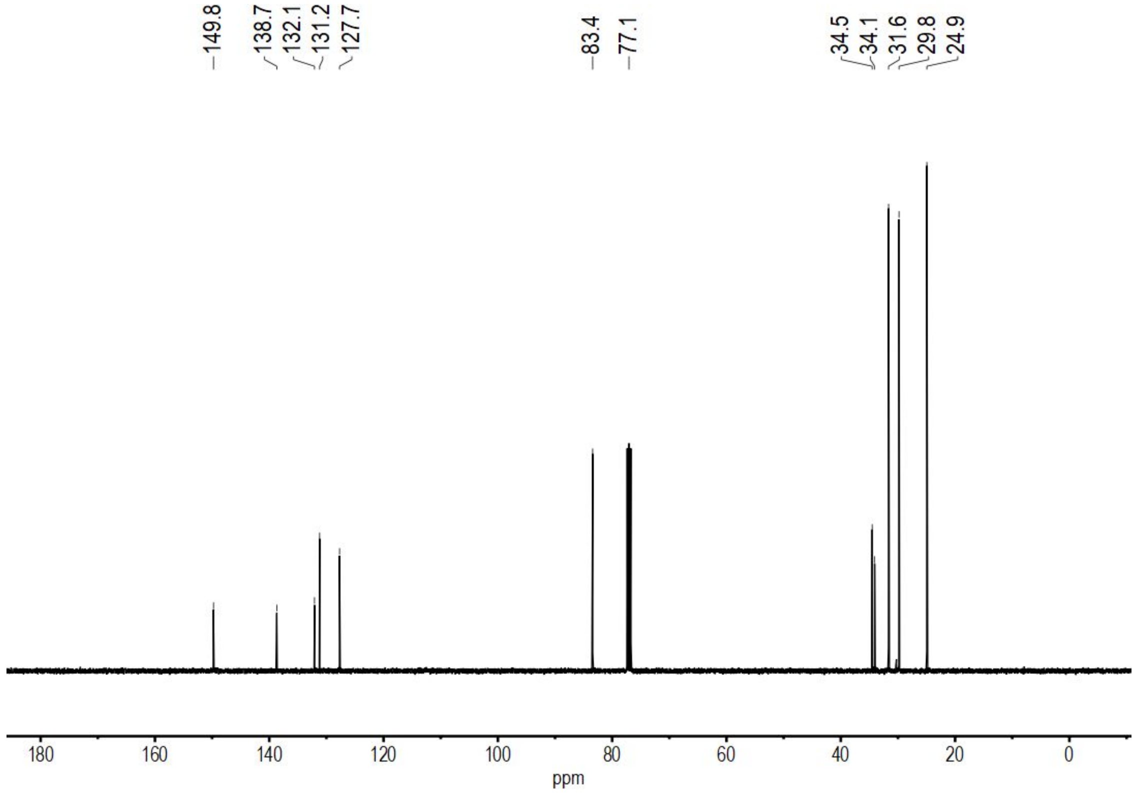

Figure S17: The solution $^{13}C\{^{1}H\}$ NMR spectrum ($CDCl_3$, 298 K, 101 MHz) of **P3''**.

S.2.3.4. Step D

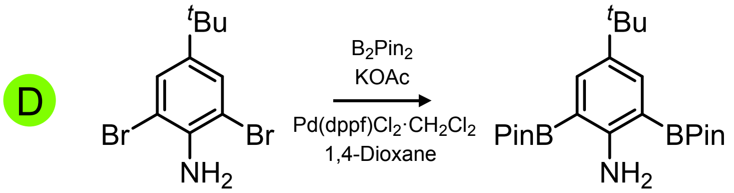

P4"

A 250 mL round bottom flask was charged with 2,6-dibromo-4-(*tert*-butyl)aniline (5.00 g, 16.3 mmol, 50 *eqv.*, M_r: 307), bis(pinacolato)diboron (12.4 g, 48.9 mmol, 150 *eqv.,* M_r: 254) and KOAc (9.57 g, 97.7 mmol, 300 *eqv.,* M_r: 98). The solids were placed under a N_2 atmosphere and suspended in dry 1,4-dioxane (80 mL). The solution was deoxygenated with bubbling N_2 for 30 minutes, before addition of $Pd(dppf)Cl_2 \cdot CH_2Cl_2$ (266 mg, 0.33 mmol, 1 *eqv.,* M_r: 816) and a further 10 minutes degassing. The mixture was heated for 18 hours at 80°C.

After cooling to room temperature, the reaction mixture was poured into ice-cooled distilled water (~200 mL) and left to stir until a permanent precipitate forms. The black solids were filtered, washed with water (3 × 20 mL) and dissolved in dichloromethane. This solution was dried over anhydrous Na_2SO_4 then passed through a short Silica plug. The now pale-yellow solution was removed to a minimum (~5 mL) under reduced pressure and product precipitated with methanol (~20 mL). The white solids were collected, washed with cooled (~5°C) methanol (2 × 5 mL) dried under reduced pressure to yield **P4”**. The methanol washings were combined, concentrated and left to stand at 5°C for 24 hours. After this time, the crystalline material was filtered to further yield **P4"** as a white solid (total: 2.75 g, 6.85 mmol, 42%, M_r: 401).

^{1}H NMR ($CDCl_3$, 298 K, 400 MHz): δ 7.72 (s, 2H), 5.91 (br. s, 2H), 1.32 (s, 24H), 1.29 (s, 9H).

^{13}C{^{1}H} NMR (**$CDCl_3$, 298 K, 101 MHz**): δ 158.4, 137.7, 137.4, 83.4, 33.9, 31.7, 25.0.

^{11}B{^{1}H} NMR (**$CDCl_3$, 298 K, 128 MHz**): δ 30.6.

ESI-MS (calc. for $[C_{22}H_{37}B_2N_1O_4\text{-}H]^+$): *m/z* 402.2992 (402.2981).

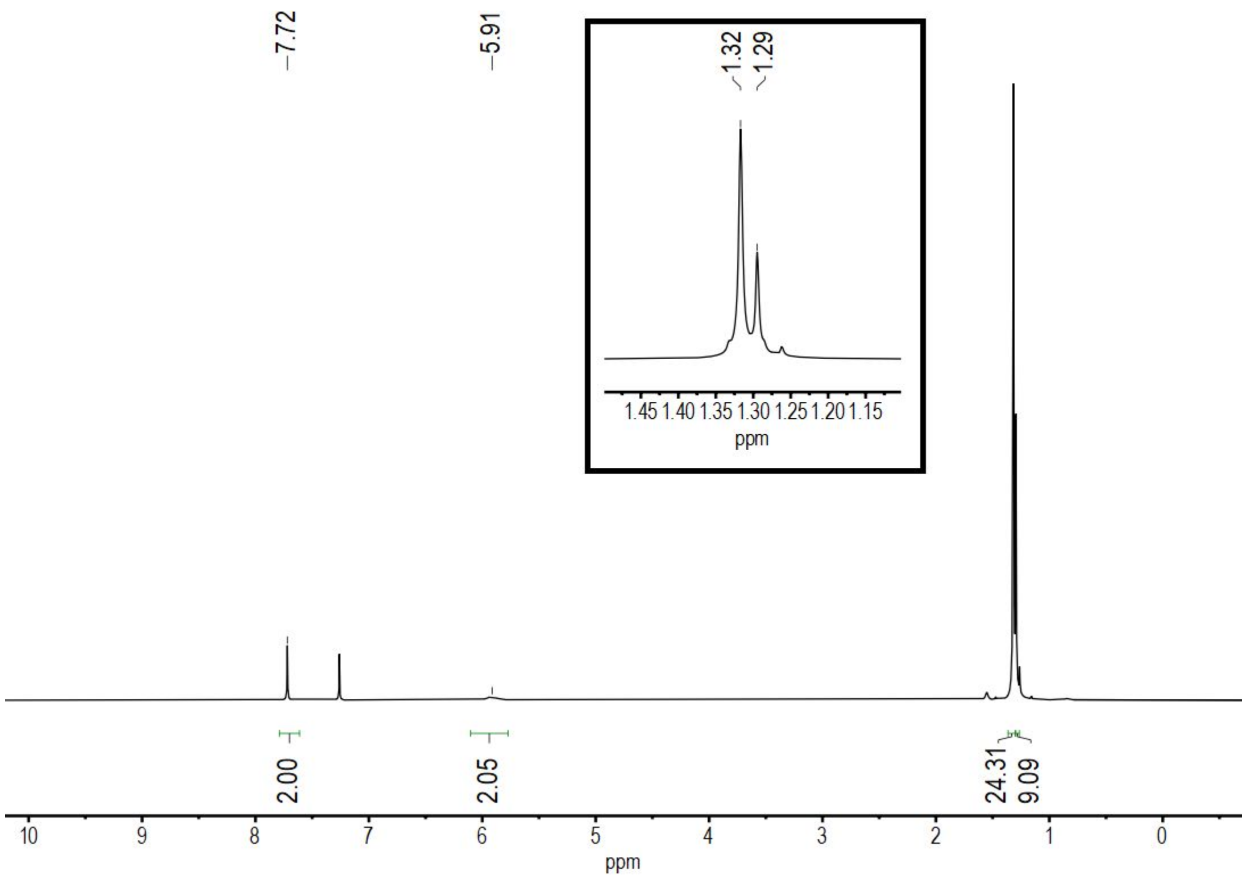

Figure S18: The solution ^{1}H NMR spectrum ($CDCl_3$, 298 K, 400 MHz) of **P4''**. The inset is an enlargement of the resonances between δ 1.5 and 1.1 ppm.

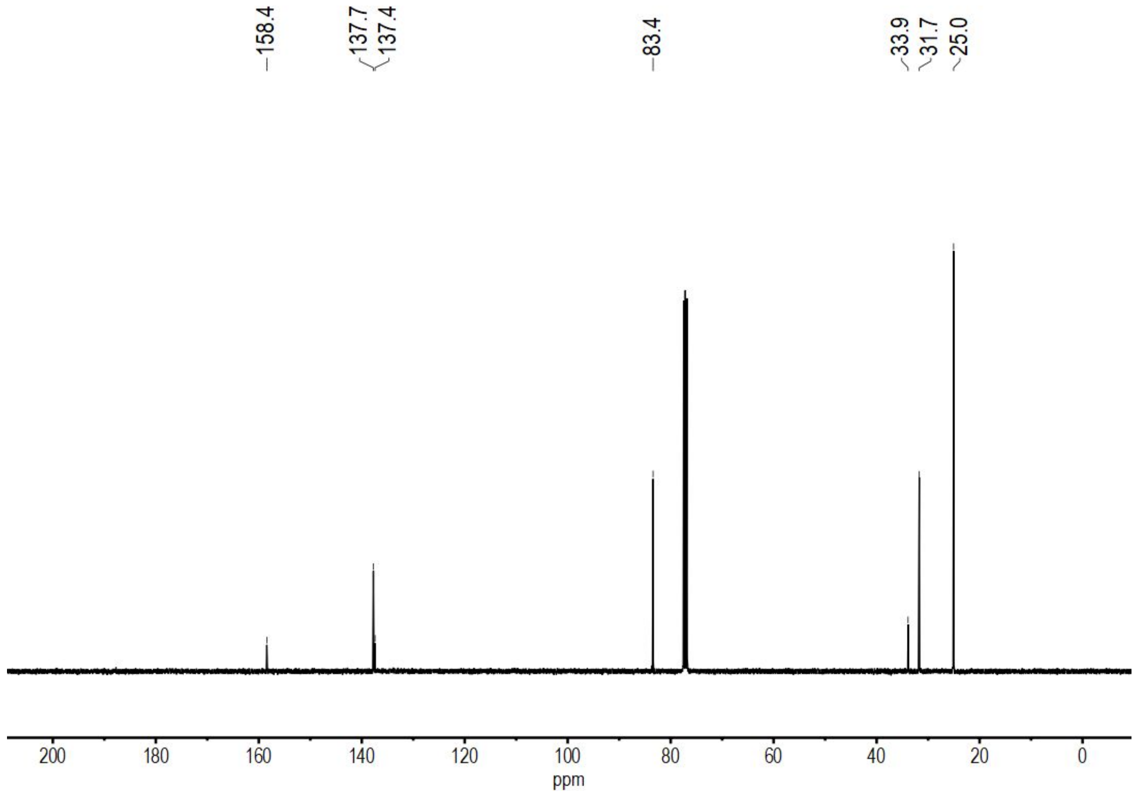

Figure S19: The solution $^{13}C\{^{1}H\}$ NMR spectrum ($CDCl_3$, 298 K, 101 MHz) of **P4''**.

S.2.3.5. Step E

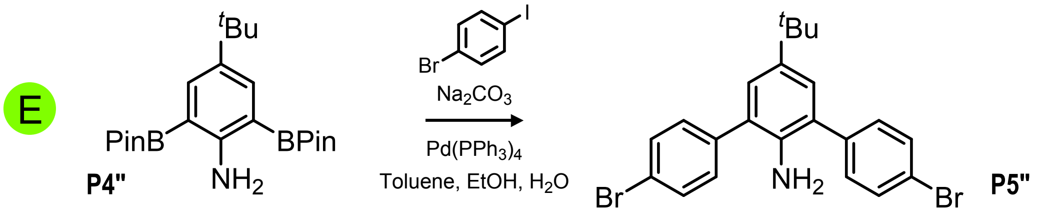

A 100 mL round bottom flask was charged with **P4"** (1.25 g, 3.11 mmol, 25 *eqv.*, M_r: 401), 1-bromo-4-iodobenzene (2.65 g, 9.35 mmol, 75 *eqv.*, M_r: 283) and Na_2CO_3 (1.98 g, 18.7 mmol, 150 *eqv.*, M_r: 106). The solids were suspended in toluene (40 mL), ethanol (10 mL) and H_2O (10 mL). The solution was deoxygenated with bubbling N_2 for 30 minutes, before addition of $Pd(PPh_3)_4$ (144 mg, 0.12 mmol, 1 *eqv.,* M_r: 1156) and a further 10 minutes degassing. The mixture was heated for 18 hours at 90°C.

After cooling to room temperature, the suspension was poured into a separation funnel and ethyl acetate (50 mL) and water (50 mL) added. The organic layers were extracted and water layer washed with further ethyl acetate (2 × 20 mL). The organic layers were combined, dried over anhydrous Na_2SO_4, solvent removed under vacuum. The solids were re-dissolved in minimum dichloromethane (~10 mL) and passed through a short Silica plug. The now pale-yellow solution was again removed. The solids were washed with methanol (3 × 15 mL), dried under vacuum and used in the next step without any further purification.

Pure material can be obtained from column chromatography (30: 70 CH_2Cl_2: pentane (R_f = 0.5)) after dry loading the crude extraction onto Silica. This yields **P5"** as a white solid (total: 1.12 g, 2.44 mmol, 78%, M_r: 459).

^{1}H NMR ($CDCl_3$, 298 K, 500 MHz): δ 7.59 (d, 4H, $^3J_{HH}$ = 8.8 Hz), 7.40 (d, 4H, $^3J_{HH}$ = 8.8 Hz), 7.12 (s, 2H), 3.66 (s, 2H), 1.32 (s, 9H).

^{13}C{^{1}H} NMR (**CD_2Cl_2, 298 K, 126 MHz**): δ 141.4, 139.0, 138.2, 132.1, 131.2, 127.1, 126.7, 121.5, 34.2, 31.7.

ESI-MS (calc. for $[C_{22}H_{21}Br_2N\text{-}H]^+$): *m/z* 460.0092 (460.0094).

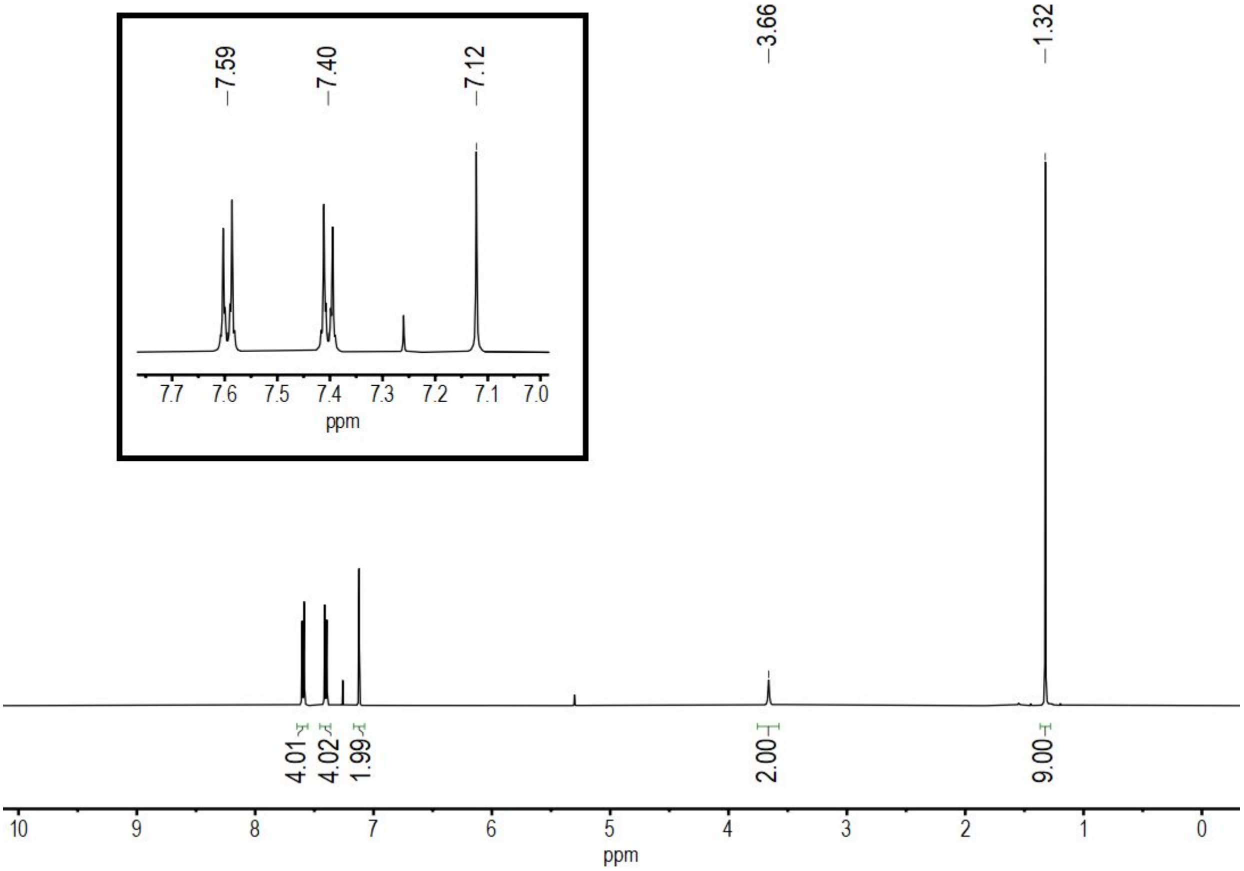

Figure S20: The solution ^{1}H NMR spectrum ($CDCl_3$, 298 K, 500 MHz) of **P5"**. The inset is an enlargement of the resonances between δ 7.8 and 7.0 ppm.

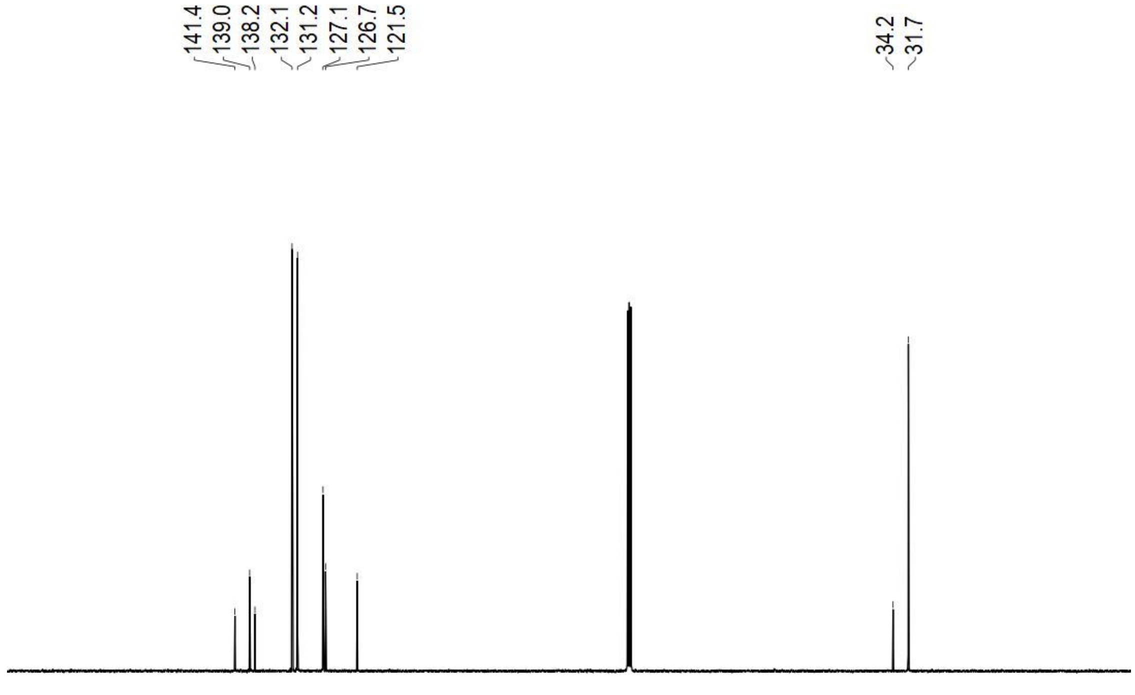

Figure S21: The solution $^{13}C\{^{1}H\}$ NMR spectrum ($CDCl_3$, 298 K, 126 MHz) of **P5"**.

S.2.3.6. Step F

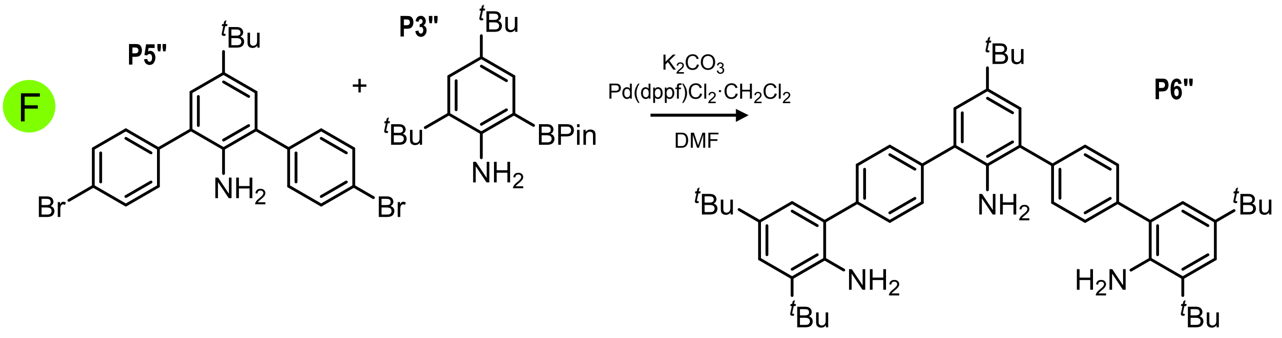

A 50 mL round bottom flask was charged with **P5"** (1.25 g, 2.72 mmol, 25 *eqv.*, M_r: 459), **P3"** (2.70 g, 8.17 mmol, 75 *eqv.*, M_r: 331), and K_2CO_3 (2.25 g, 16.3 mmol, 150 *eqv.* M_r: 138). The solids were suspended in dry DMF (30 mL) and solution deoxygenated with bubbling N_2 for 30 minutes, before the addition of $Pd(dppf)Cl_2 \cdot CH_2Cl_2$ (88 mg, 0.11 mmol, 1 *eqv.,* M_r: 816) and a further 10 minutes degassing. The mixture was heated for 2 hours at 110°C.

After cooling to room temperature, the suspension was poured into water (~150 mL) and grey precipitate collected. The solids were washed with further water (3 × 30 mL), before being extracted into dichloromethane (3 × 15 mL). The organic layers were combined, dried over anhydrous Na_2SO_4, solvent removed under vacuum. The solids were then re-dissolved in minimum dichloromethane and passed through a short Silica plug. The yellow solution was removed to a minimum (~5 mL) and methanol added (~40 mL). The resulting precipitate was filtered and used in the next step without any further purification.

Pure material can be obtained from column chromatography (15: 85 ethyl acetate: cyclohexane (R_f = 0.5)) after dry loading the crude extraction onto Silica. This yields **P6"** as an off-white solid (1.21 g, 1.71 mmol, 63%, M_r: 708).

^{1}H NMR ($CDCl_3$, 298 K, 400 MHz): δ 7.67 (d, 4H, $^3J_{HH}$ = 8.1 Hz), 7.60 (d, 4H, $^3J_{HH}$ = 8.1 Hz), 7.40 (d, 2H, $^4J_{HH}$ = 2.3 Hz), 7.27 (s, 2H), 7.13 (d, 2H, $^4J_{HH}$ = 2.3 Hz), 3.96 (br. s, 6H), 1.54 (s, 18H), 1.40 (s, 9H), 1.37 (s, 18H).

^{13}C{^{1}H} NMR (**$CDCl_3$, 298 K, 101 MHz**): δ 141.2, 140.4, 139.8, 139.4, 139.0, 138.6, 133.3, 130.3, 129.9, 128.8, 127.5, 127.1, 125.6, 123.4, 77.2, 35.0, 34.4, 31.8, 31.8, 30.1.

ESI-MS (calc. for $[C_{50}H_{65}N_3\text{-}H_2]^{2+}$): *m/z* 354.7668 (354.7662).

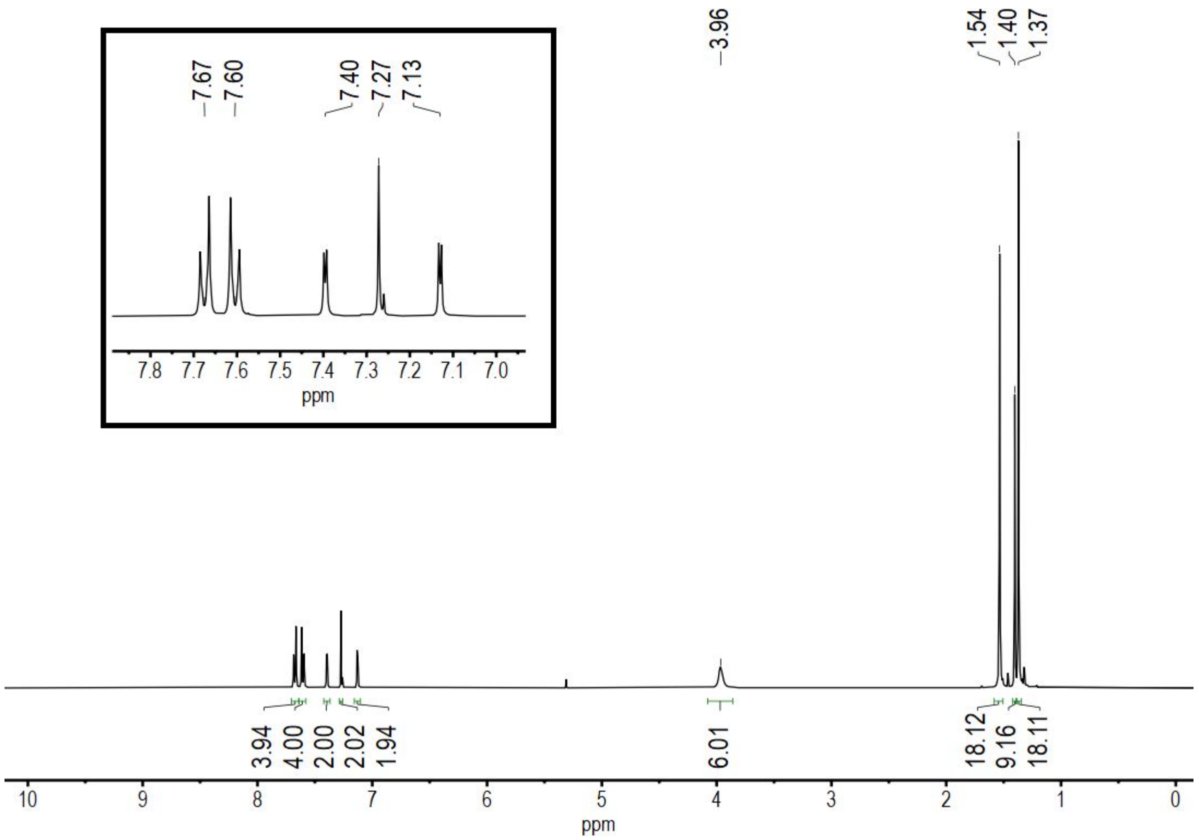

Figure S22: The solution ^{1}H NMR spectrum ($CDCl_3$, 298 K, 400 MHz) of **P6"**. The inset is an enlargement of the resonances between δ 7.9 and 6.9 ppm.

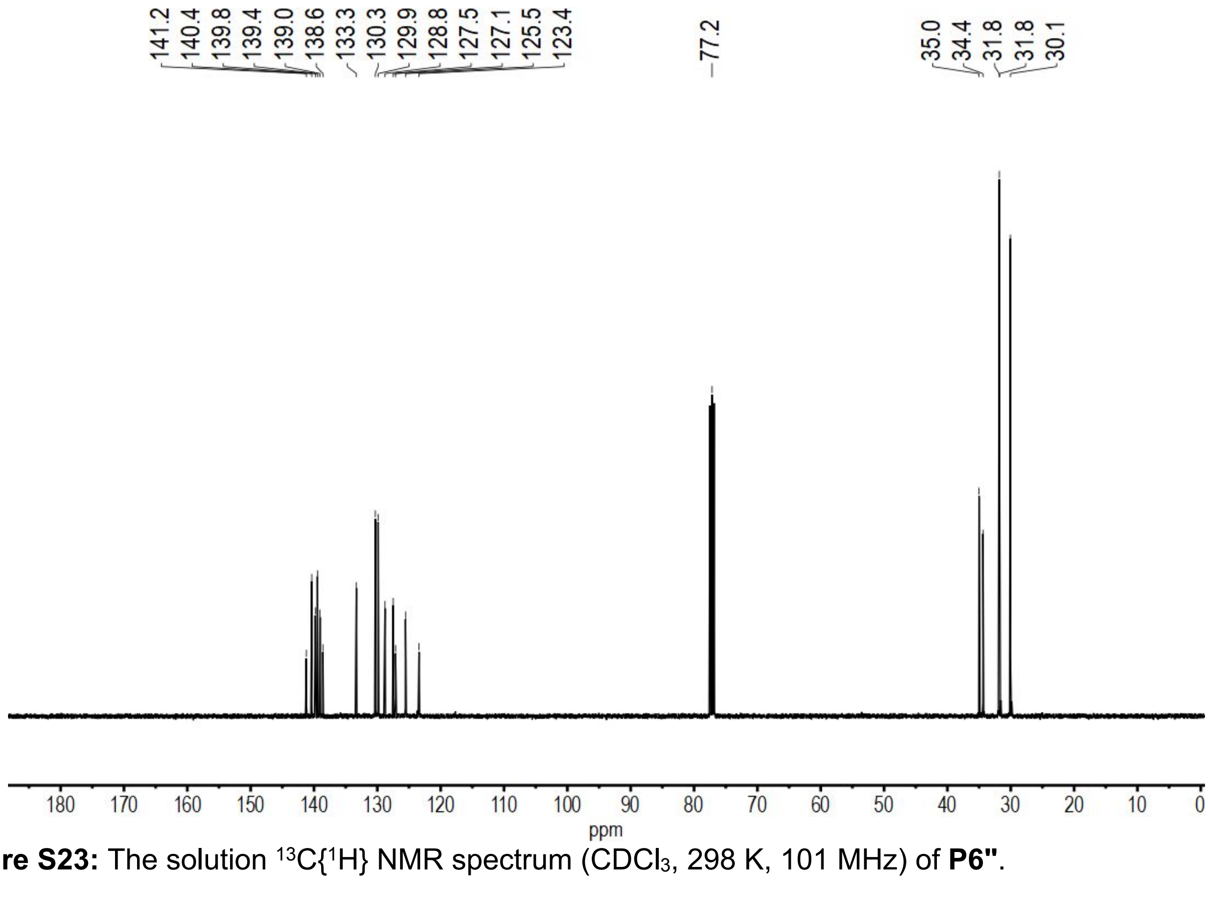

Figure S23: The solution $^{13}C\{^{1}H\}$ NMR spectrum ($CDCl_3$, 298 K, 101 MHz) of **P6"**.

S.2.3.7. Step G

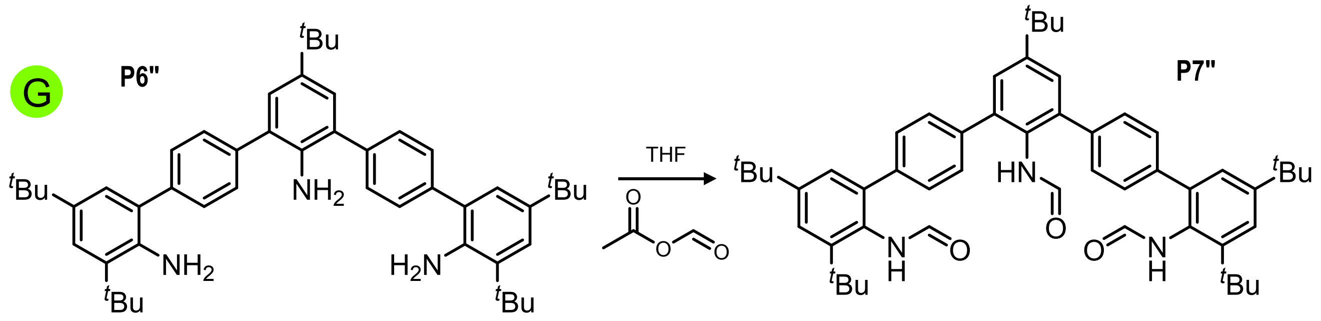

A 50 mL round bottom flask was charged with **P6"** (800 mg, 1.13 mmol, M_r: 708) and suspended in THF (30 mL). Freshly synthesised acetic formic anhydride (5.1 mL) was added and reaction left to stir for 16 hours at room temperature.

After this time, the reaction mixture was poured into ice-cooled distilled water (~150 mL) and left to stir until a permanent precipitate formed. The precipitate was filtered, washed with water (3 × 50 mL), dichloromethane (3 × 5 mL) and finally pentane (3 × 20 mL) before drying under reduced pressure to yield **P7"** as an off-white solid (740 mg, 0.93 mmol, 83%, M_r: 792).

Note: Acetic formic anhydride was synthesised by slow addition of formic acid (1.5 mL) to acetic anhydride (3 mL) at 0°C followed by deoxygenating with bubbling N_2 for 30 minutes. The mixture was heated to 50°C for 2 hours, cooled and opened to air to use as a neat reagent. A ratio of 1.5 mL acetic formic anhydride : 1 mmol $-NH_2$ was used.

^{1}H NMR (DMSO-*d*$_6$, 298 K, 500 MHz): δ 9.6 – 9.1 (br. m., 3H), 7.9 – 7.0 (br. m., 17H), 1.6 – 1.2 (m., 45H).

ESI-MS (calc. for $[C_{53}H_{65}N_3O_3\text{-}H]^+$): *m/z* 792.5109 (792.5099).

Note: Due to the rotameric effects and zwitterion forms of the formamide group, the full characterisation could not be deciphered through spectroscopy methodologies. This is consistent with previous reported systems by Bilger *et. al.* (*J. Am. Chem. Soc.*, **2021**, *143*, 1651)[S6] and Herr *et. al.* (*Nat. Chem.*, **2021**, *13,* 956).[S7]

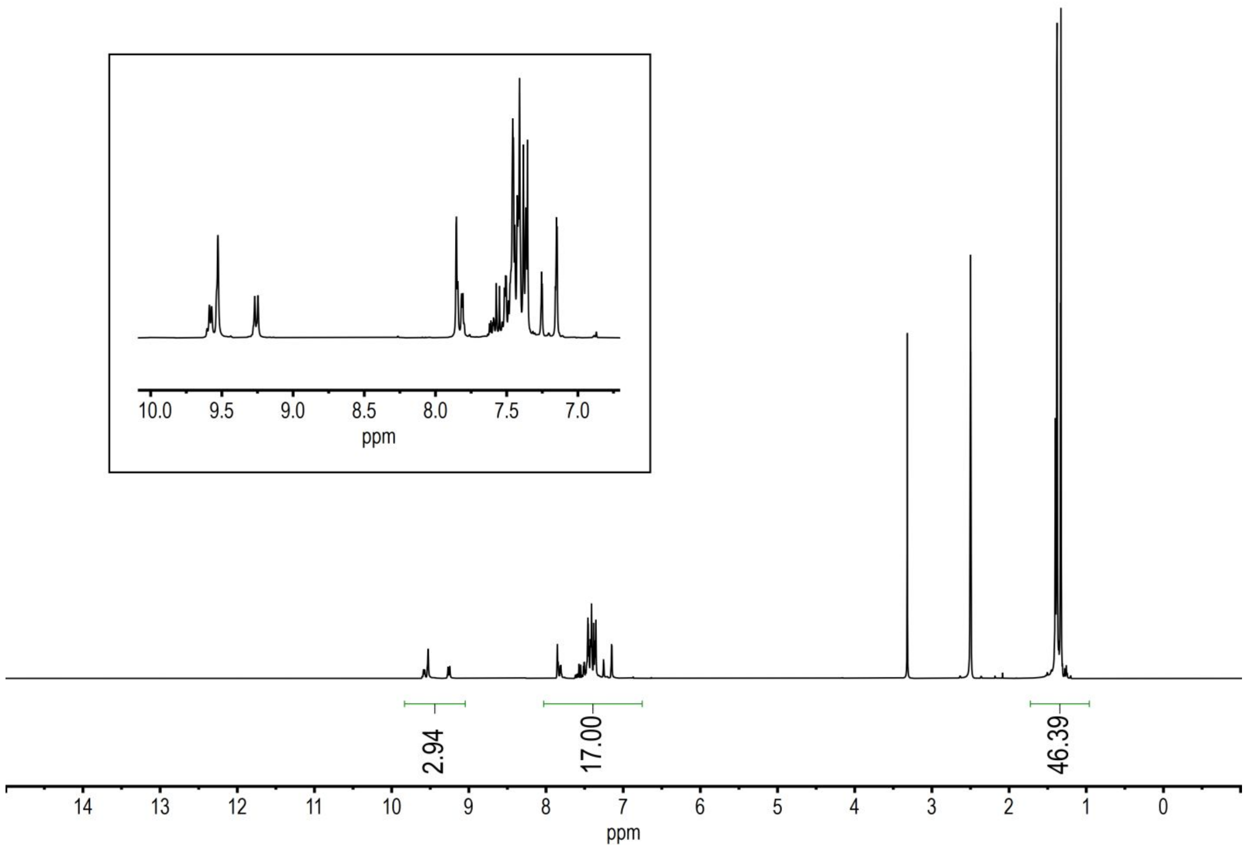

Figure S24: The solution ^{1}H NMR spectrum (DMSO-d_6, 298 K, 500 MHz) of **P7"**. The inset is an enlargement of the resonances between δ 10.0 and 7.0.

S.2.3.8. Step H

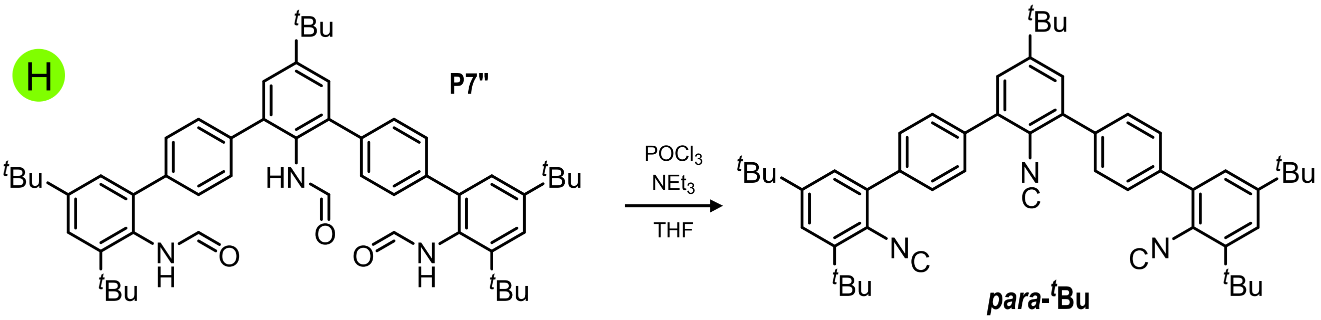

A 25 mL round bottom flask was charged with **P7"** (700 mg, 0.88 mmol, 1 *eqv.*, M_r: 792) and placed under a nitrogen atmosphere. Dry THF (20 mL) and triethylamine (2.98 mL, 21.2 mmol, 24 *eqv.*) were added and suspension cooled to 0°C. $POCl_3$ (1.24 mL, 13.3 mmol, 15 *eqv.*) was added dropwise over 5 minutes before being left to warm to room temperature and stir for 16 hours.

After this time, and working under air, the orange suspension was added dropwise over 10 minutes to an ice-cooled solution of saturated aqueous Na_2CO_3 solution (20 mL) and water (20 mL). The reaction was left to quench for 30 minutes at this temperature. The off-white precipitate was then filtered, washed with water (3 × 50 mL) and extracted into dichloromethane. The solution was dried over anhydrous Na_2SO_4, filtered and dry loaded onto Silica. Purification by column chromatography (50: 50 CH_2Cl_2: pentane (R_f = 0.6)) yielded ***para-*^{t}Bu** as a white solid (520 mg, 0.70 mmol, 80%, M_r: 738).

^{1}H NMR (CD_2Cl_2, 298 K, 400 MHz): δ 7.72 (d, 4H, $^3J_{HH}$ = 8.2 Hz, **H_b**), 7.63 (d, 4H, $^3J_{HH}$ = 8.2 Hz, **H_c**), 7.55 (s, 2H, **H_a**), 7.52 (d, 4H, $^4J_{HH}$ = 2.1 Hz, **H_e**), 7.36 (d, 4H, $^4J_{HH}$ = 2.1 Hz, **H_d**), 1.58 (s, 18H, **H_g**), 1.43 (s, 9H, **H_h**), 1.37 (s, 18H, **H_f**).

^{13}C{^{1}H} NMR (CD_2Cl_2, 298 K, 101 MHz): δ 172.7 (**C_t**), 169.6 (**C_u**), 153.4, 152.5, 146.1, 141.0, 139.4, 139.4, 138.2, 129.9 (**C_e**), 129.6 (**C_f**), 127.5 (**C_b**), 126.1 (**C_i**), 123.7 (**C_k**), 121.3, 120.6, 35.9 (**C_p** or **C_r**), 35.5 (**C_n**), 35.5 (**C_p** or **C_r**), 31.3 (**C_s**), 31.3 (**C_o**), 29.5 (**C_q**).

NOESY ^{1}H NMR (CD_2Cl_2, 298 K, 500 MHz): Cross-peak between δ 7.72 with 7.55, 7.63 with 7.36, 7.52 with 1.58 and 1.37, 7.36 with 1.37.

ESI-MS (calc. for $[C_{53}H_{59}N_3\text{-}Na]^+$): *m/z* 760.4594 (760.4601).

Elemental Analysis (calc. for $C_{53}H_{59}N_3$): C 86.32 (86.25), H 7.86 (8.06), N 5.54 (5.69).

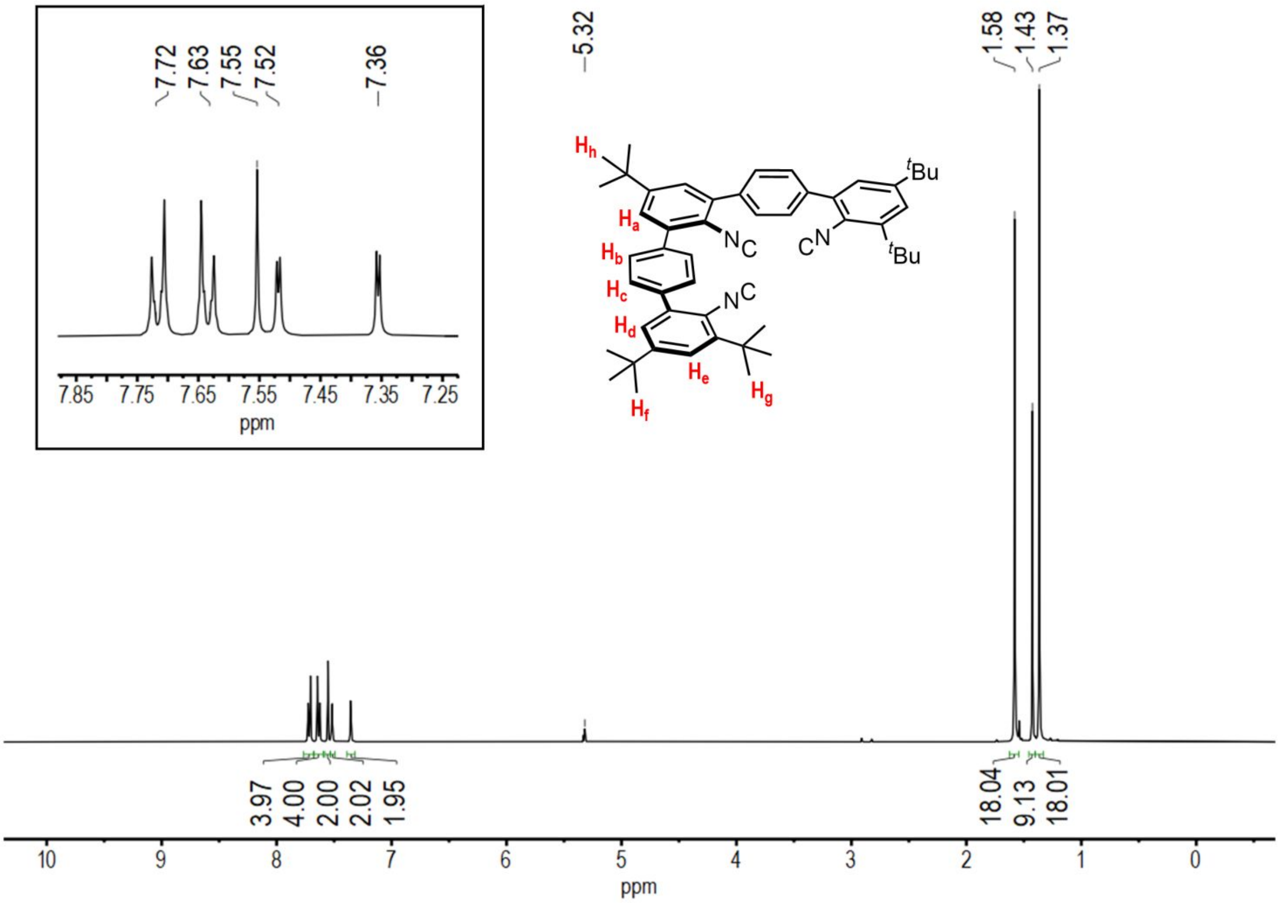

Figure S25: The solution ^{1}H NMR spectrum (CD_2Cl_2, 298 K, 400 MHz) of ***para-*** ***para-^{t}Bu***. The inset is an enlargement of the resonances between δ 7.8 and 7.2 ppm.

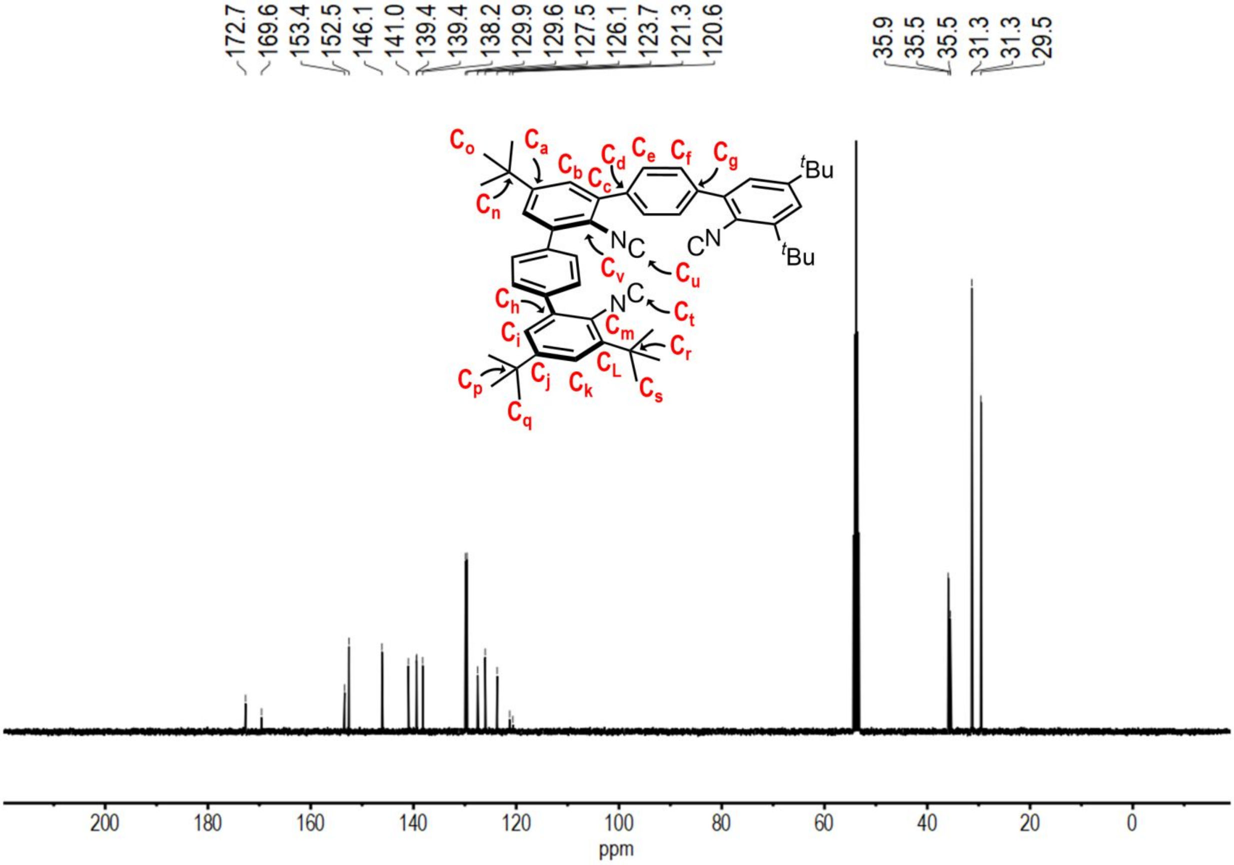

Figure S26: The solution $^{13}C\{^{1}H\}$ NMR spectrum (CD_2Cl_2, 298 K, 101 MHz) of ***para-^{t}Bu***.

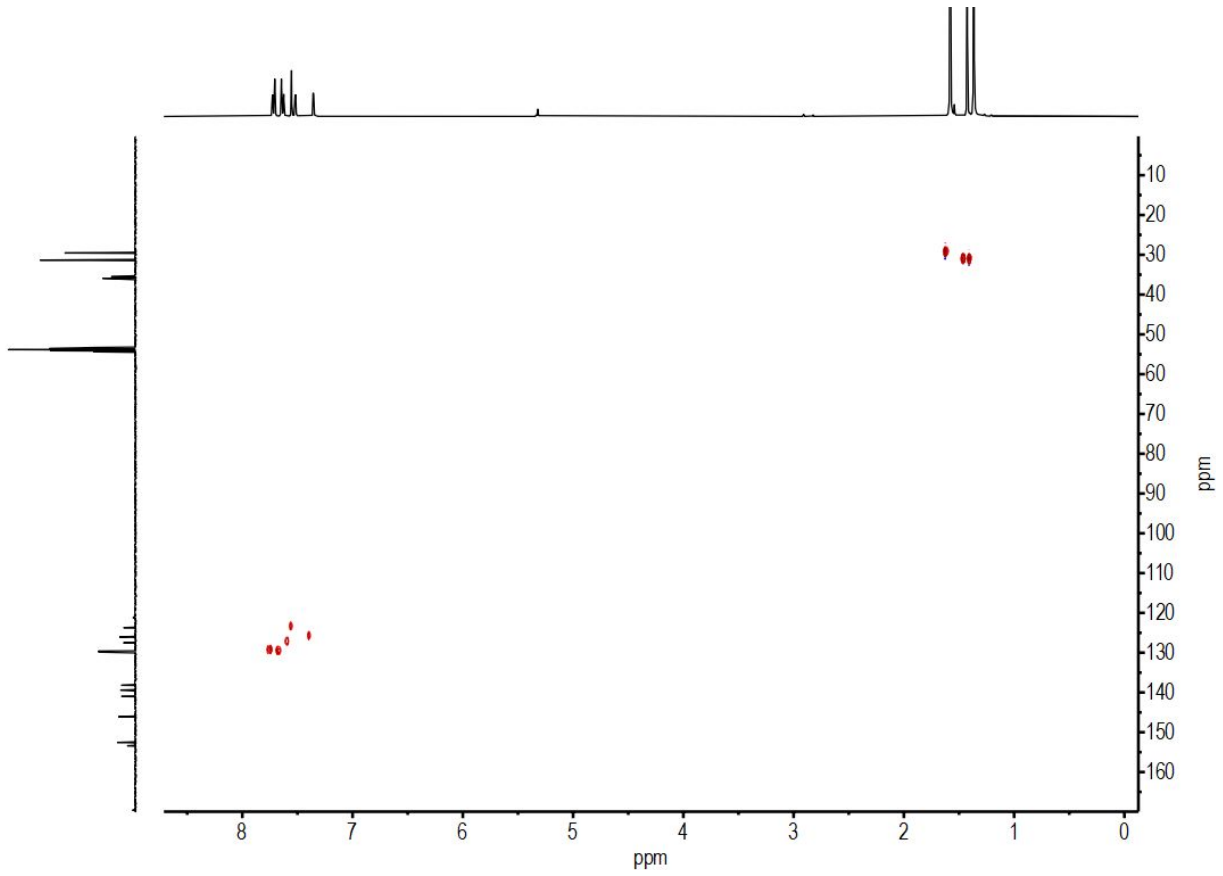

Figure S27: The solution ^{1}H-^{13}C HMQC spectrum (CD_2Cl_2, 298 K) of ***para-^{t}Bu***.

S.2.4. [1,1'-biphenyl]-3,3'-diisocyanide, 2-CN

Procedure Overview:

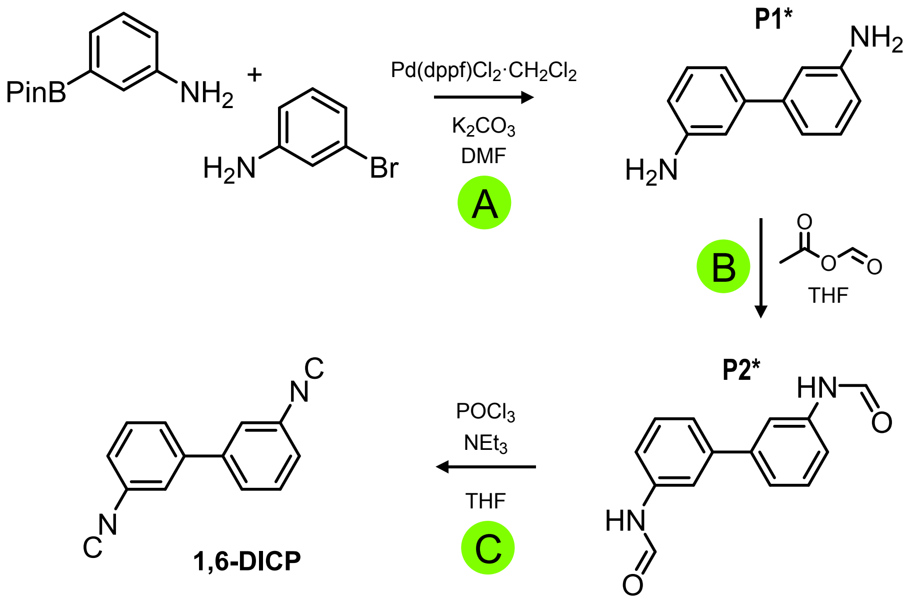

Note: Product **P1*** has been reported by Reuter *et. al.* (*Chem. Eur. J.,* **2011**, *17*, 2987)[S9] and was not adapted from their procedure. The overall procedure has been inspired by Luo *et. al.* (*CCS Chem.*, **2022**, *4*, 2897).[S10] Characterization data and spectra are given for all new products beneath each procedure.

S.4.1.1. Step B

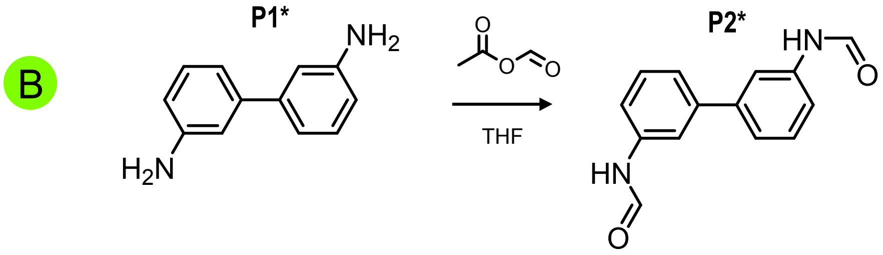

A 25 mL round bottom flask was charged with **P1*** (750 mg, 4.08 mmol, M_r: 184) and the latter was dissolved in THF (10 mL). Freshly synthesised acetic formic anhydride (1.35 mL) was added dropwise over 5 minutes and reaction left to stir for 3 hours.

After this time, the solution was poured into ice-cooled distilled water (~150 mL) and left to stir until a permanent precipitate formed. The precipitate was filtered, washed with water (3 × 50 mL) then pentane (3 × 10 mL). The product was extracted into THF (3 × 15 mL), dried over anhydrous Na_2SO_4 and solvent removed to yield the formyl amide product **P2*** as an off-white solid (690 mg, 2.89 mmol, 71%, M_r: 240).

Note: Acetic formic anhydride was synthesised by slow addition of formic acid (1.5 mL) to acetic anhydride (3 mL) at 0°C followed by deoxygenating with bubbling N_2 for 30 minutes. The mixture was heated to 50°C for 2 hours, cooled and opened to air to use as a neat reagent. A ratio of 1.5 mL acetic formic anhydride : 1 mmol $–NH_2$ was used.

^{1}H NMR (DMSO-*d*$_6$, 298 K, 400 MHz): δ ~10.3 (m., ~2H), 8.90 – 7.45 (br. m., ~10H).

ESI-MS (calc. for $[C_{14}H_{12}N_2O_2\text{-}H]^+$): *m/z* 241.0987 (241.0977).

Note: Due to the rotameric effects and zwitterion forms of the formamide group, the full characterisation could not be deciphered through spectroscopy methodologies. This is consistent with previous reported systems by Bilger *et. al.* (*J. Am. Chem. Soc.*, **2021**, *143*, 1651)[S6] and Herr *et. al.* (*Nat. Chem.*, **2021**, *13,* 956).[S7]

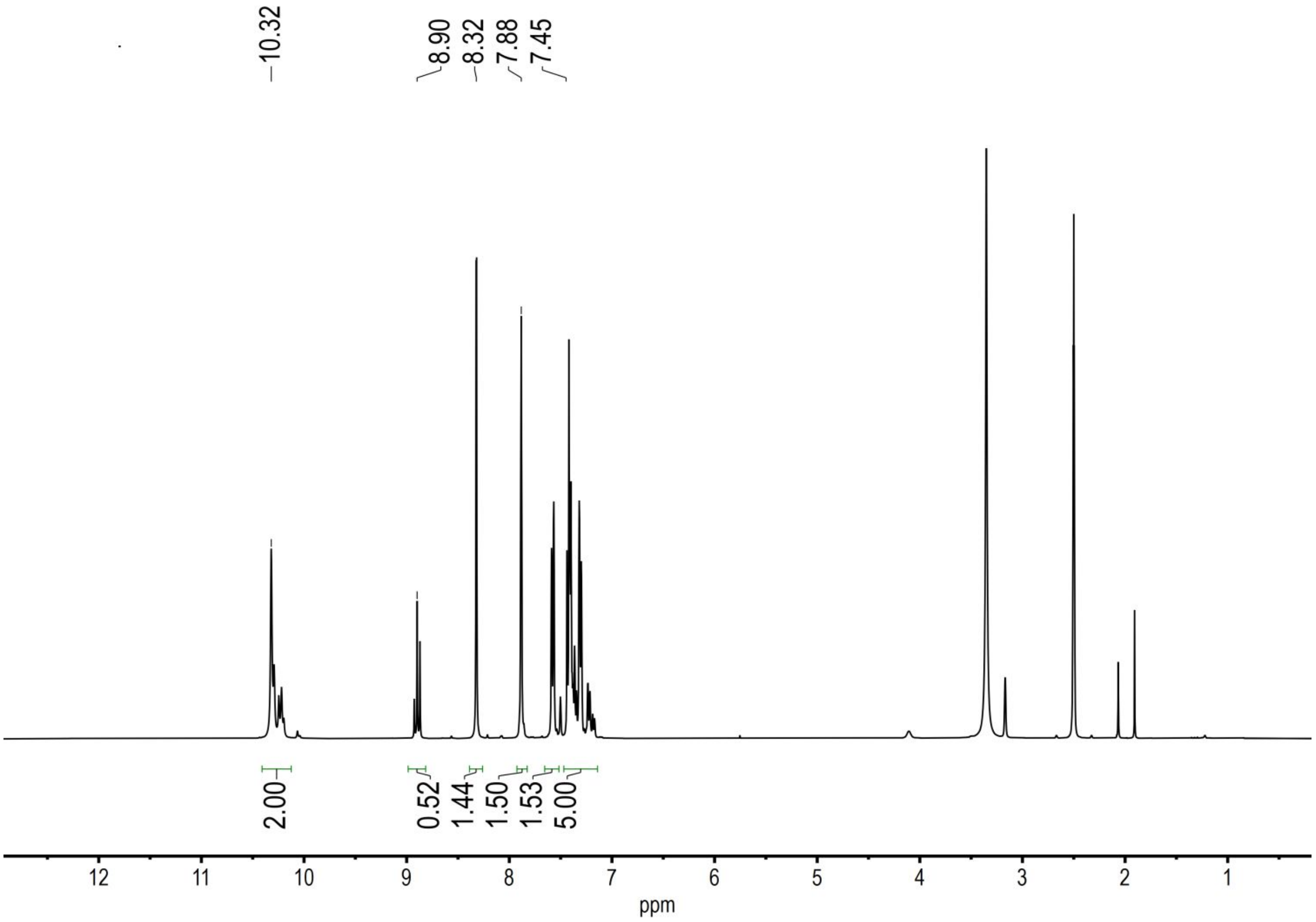

Figure S28: The solution ^{1}H NMR spectrum (DMSO-d_6, 298 K, 400 MHz) of **P2***.

S.4.1.2. Step C

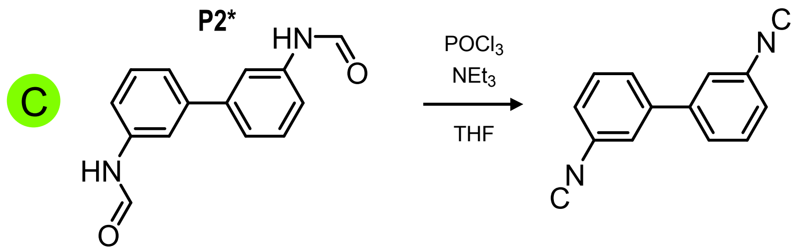

A 25 mL round bottom flask was charged with **P2*** (500 mg, 2.08 mmol, 1 *eqv.*, M_r: 240) and placed under a nitrogen atmosphere. Dry THF (20 mL) and triethylamine (4.64 mL, 33.3 mmol, 16 *eqv.*) were added and suspension cooled to 0°C. $POCl_3$ (1.94 mL, 20.8 mmol, 10 *eqv.*) was added dropwise over 5 minutes before being left to warm to room temperature and stir for 16 hours.

After this time, and working under air, the orange suspension was added dropwise over 10 minutes to an ice-cooled solution of saturated aqueous Na_2CO_3 solution (20 mL) and water (20 mL). The reaction was left to quench for 30 minutes at this temperature. The off-white precipitate was then filtered, washed with water (3 × 50 mL) and methanol (3 × 20 mL) to yield [1,1'-biphenyl]-3,3'-diisocyanide, **2-CN**, as a white solid (250 mg, 1.23 mmol, 59%, M_r: 204).

^{1}H NMR (CD_2Cl_2, 298 K, 500 MHz): δ 7.61 (m., 4H, **H_c** & **H_b**), 7.52 (t., 2H, $^{3}J_{HH}$ = 8.3 Hz, **H_c**), 7.43 (m., 2H, **H_d**).

^{13}C{^{1}H} NMR (**CD_2Cl_2, 298 K, 126 MHz**): δ 165.7 (**C_g**), 141.0 (**C_b**), 130.6 (**C_d**), 128.4 (**C_a**), 127.7 (**C_d**), 126.4 (**C_e**), 125.4 (**C_c**).

ESI-MS (calc. for $[C_{14}H_8N_2\text{-}H]^+$): *m/z* 205.0764 (205.0760).

Elemental Analysis (calc. for $C_{14}H_8N_2{\cdot}1.5H_2O$): C 72.59 (72.71), H 4.99 (4.76), N 11.80 (12.11).

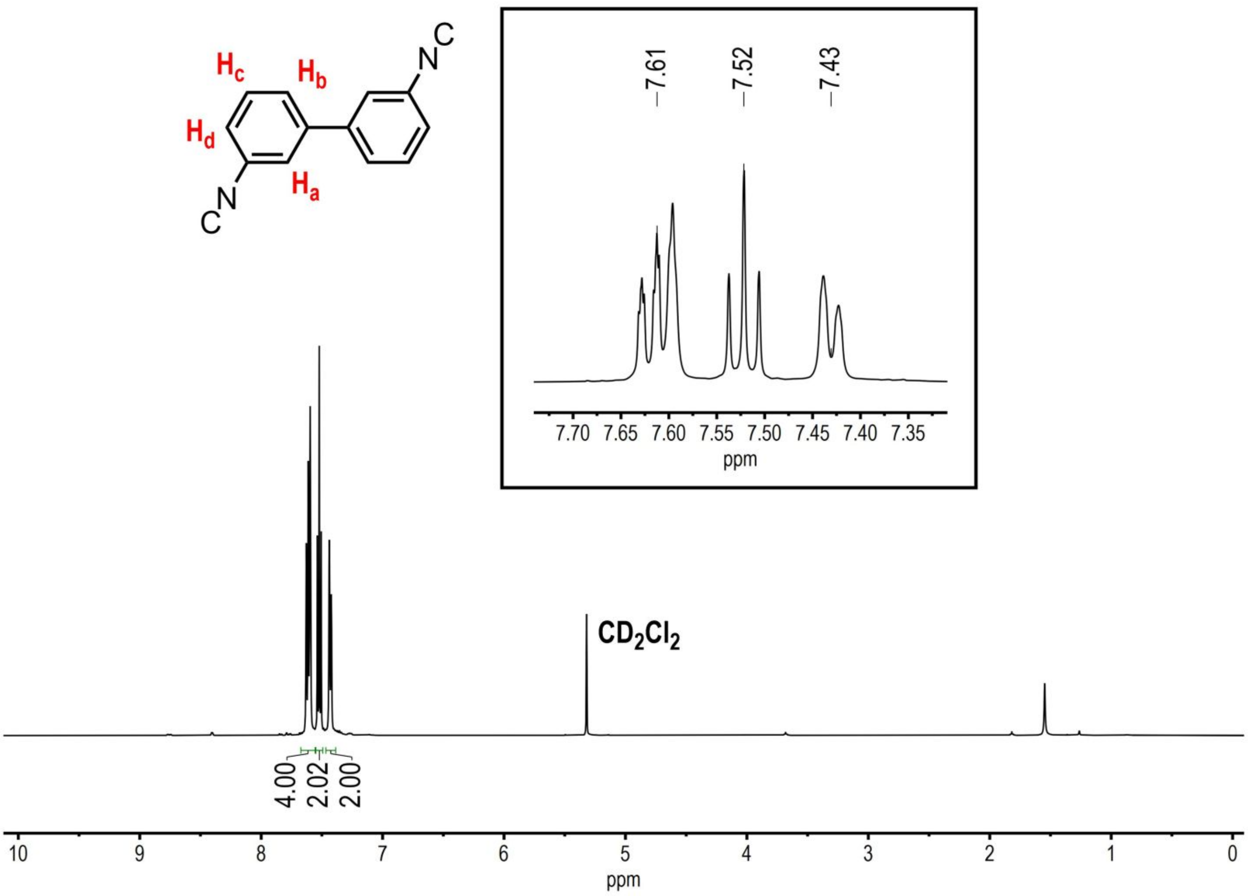

Figure S29: The solution ^{1}H NMR spectrum (CD_2Cl_2, 298 K, 500 MHz) of **2-CN**. The inset is an enlargement of the resonances between δ 7.7 and 7.3 ppm.

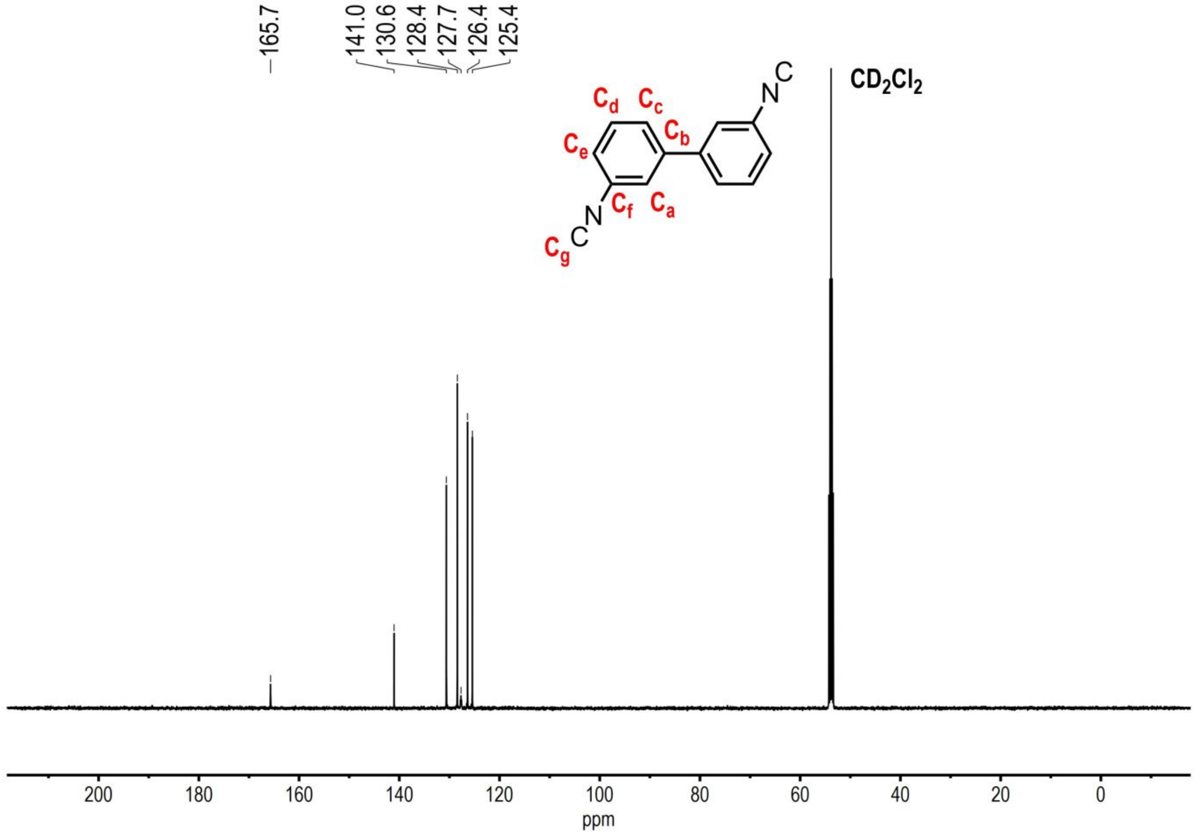

Figure S30: The solution $^{13}C\{^1H\}$ NMR spectrum (CD_2Cl_2, 298 K, 126 MHz) of **2-CN**.

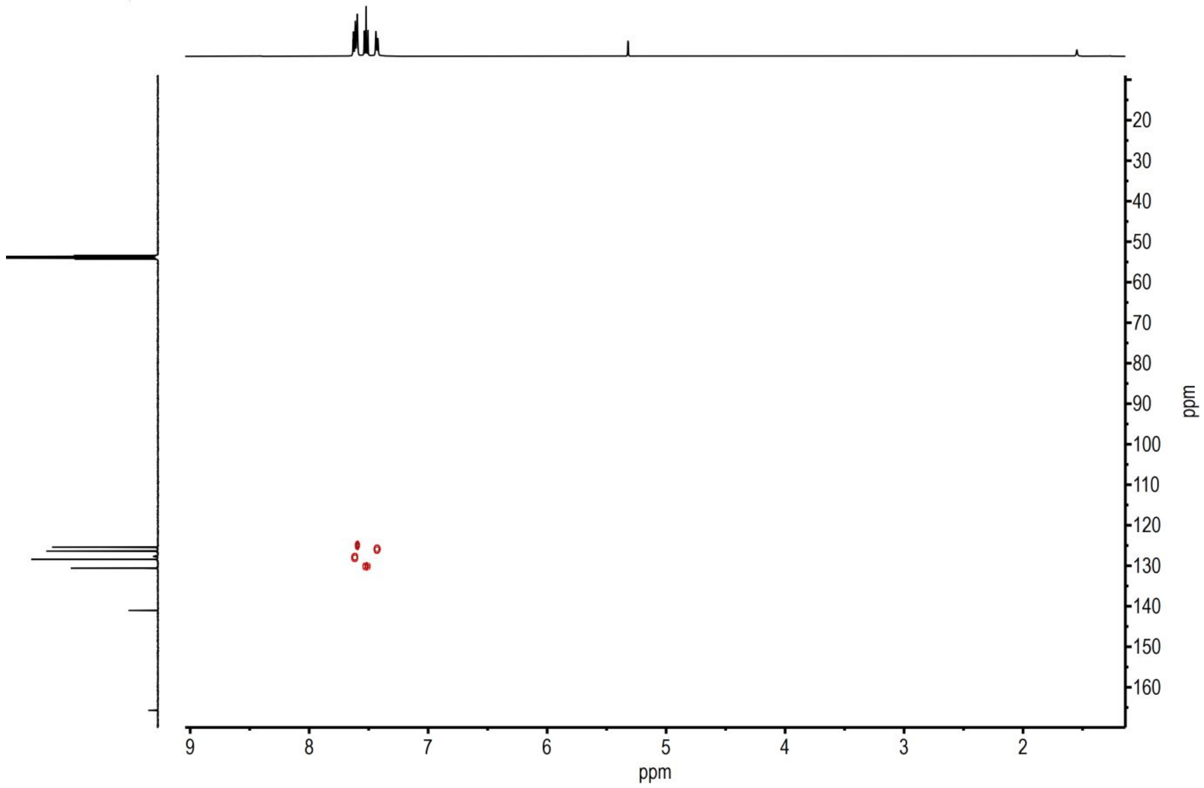

Figure S31: The solution ^{1}H-^{13}C HMQC spectrum (CD_2Cl_2, 298 K) of **2-CN**.

S.3. Organometallic Synthesis

S.4.1. Rh(*meta*-Me)Cl

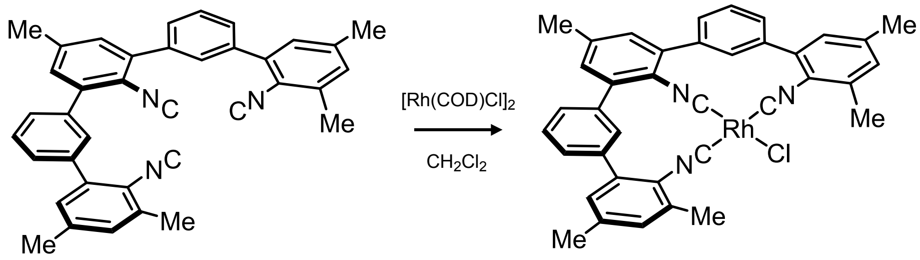

Working using Schlenk line techniques, a 10 mL round bottom flask was charged with ***meta*-Me** (100 mg, 189 µmol, 2 *eqv*., M_r: 528) and $[Rh(COD)Cl]_2$ (47 mg, 94.7 µmol, 1 *eqv*., M_r: 493). The flask was placed under a N_2 atmosphere and dried and deoxygenated dichloromethane (5 mL) was added. The orange solution was left to stir for 1 hour, which turns pale-yellow in colour.

After this time, and working under air, the solution was filtered. Pentane (~20 mL) was added and solution left to stir until a permanent precipitate formed. The pale-yellow solids were filtered, washed with -18°C acetonitrile (2 × 2 mL) then pentane (2 × 5 mL) and dried to yield **Rh(*meta*-Me)Cl** (87 mg, 131 µmol, M_r: 666, 69%) as an air-stable yellow solid.

^{1}H NMR (CD_2Cl_2, 298 K, 500 MHz): δ 7.71 (t, 2H, $^4J_{HH}$ = 1.5 Hz **H_e**), 7.63 (app. t., 2H, $^3J_{HH}$ = 7.8 Hz, $^3J_{HH}$ = 7.6 Hz, **H_c**), 7.46 (d. of t., 2H, $^3J_{HH}$ = 7.8 Hz, $^4J_{HH}$ = 1.5 Hz **H_d**), 7.41 (d. of t., 2H, $^3J_{HH}$ = 7.6 Hz, $^4J_{HH}$ = 1.5 Hz **H_b**), 7.21 (s., 2H, **H_a**), 7.13 (d, 2H, $^4J_{HH}$ = 1.6 Hz, **H_g**), 7.03 (d, 2H, $^4J_{HH}$ = 1.6 Hz, **H_f**), 2.46 (s, 6H, **H_i**), 2.41 (s, 3H, **H_h**), 2.36 (s, 6H, **H_j**).

^{13}C{^{1}H} NMR (CD_2Cl_2, 298 K, 126 MHz): δ 151.2 (d, ~2C, $^1J_{RhC}$ = 58 Hz, **C_q**)*, 148.3 (d, ~1C, $^1J_{RhC}$ = 66 Hz, **C_r**)*, 140.2 (**C_o**), 139.6, 138.8, 138.8 (**C_a**), 138.2, 136.2, 131.2 (**C_b**), 130.9 (**C_n**), 130.1 (**C_g**), 129.8 (**C_L**), 129.8 (**C_f**), 129.8 (**C_h**), 129.8, 129.5 (**C_j**), 122.6 (**C_m**), 122.6 (**C_c**), 21.6 (**C_u**), 21.5 (**C_s**), 19.1 (**C_t**).

* = coupling constants did not change when ^{13}C{^{1}H} NMR experiment conducted at 101 MHz.

Selected NOESY ^{1}H NMR (CD_2Cl_2, 298 K, 500 MHz) peaks: Cross-peak between δ 7.46 with 7.03, 7.13 with 2.46 and 2.36 and 7.03 with 2.46 ppm.

ESI-MS (calc. for $[Rh(C_{38}H_{29}N_3)(CH_3CN)]^+$): *m/z* 671.1670 (671.1677).

Elemental Analysis (calc. for $Rh(C_{38}H_{29}N_3)Cl{\cdot}2.5\ H_2O$): C 64.25 (64.19), H 4.65 (4.82), N 5.54 (5.91).

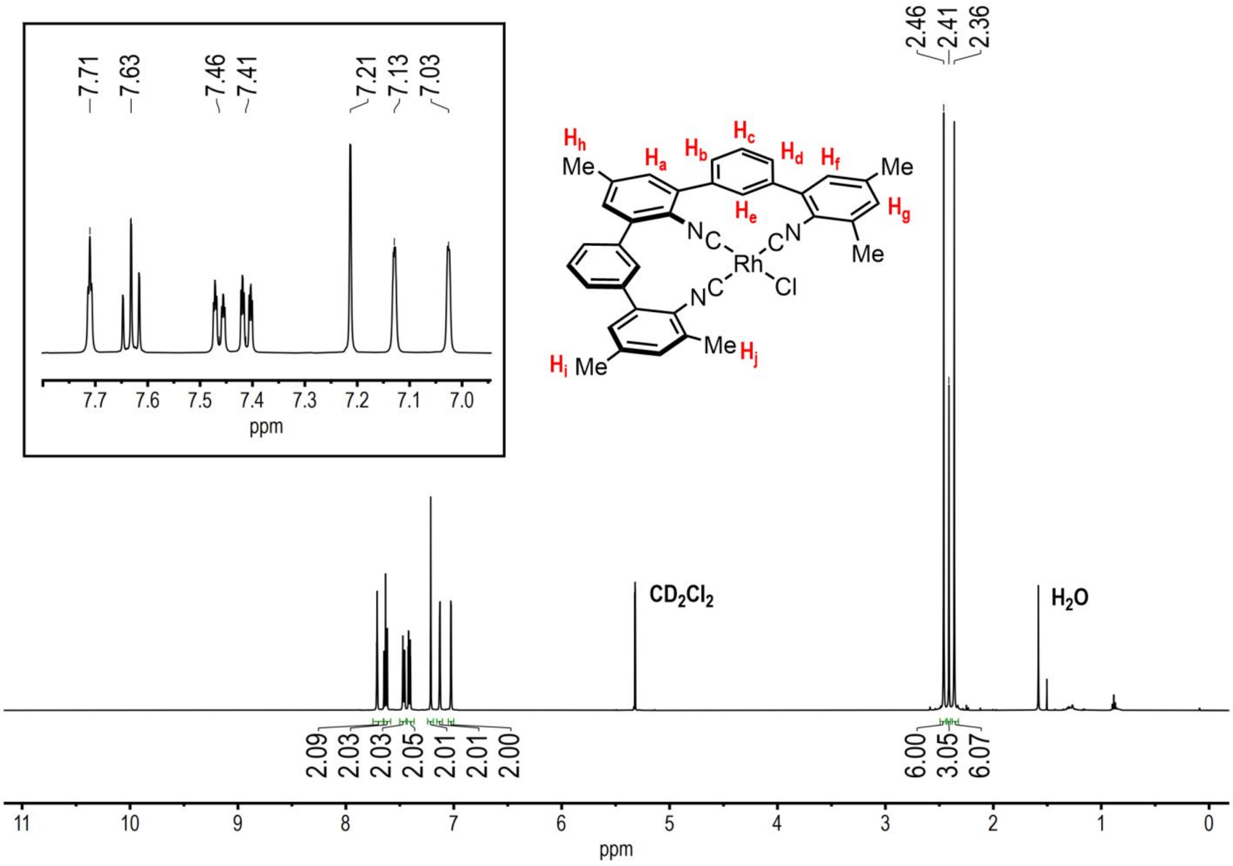

Figure S32: The solution ^{1}H NMR spectrum (CD_2Cl_2, 298 K, 500 MHz) of **Rh(*meta*-Me)Cl**. The inset is an enlargement of the resonances between δ 7.8 and 7.0 ppm.

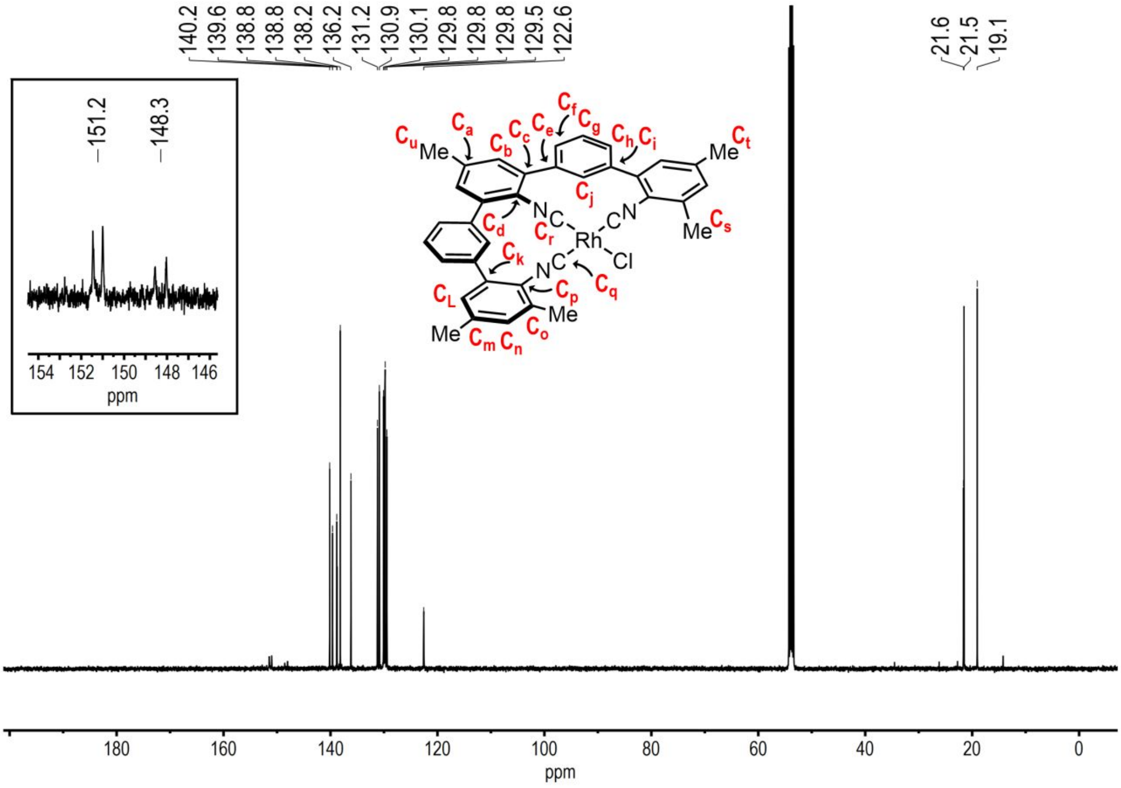

Figure S33: The solution ^{13}C{^{1}H} NMR spectrum (CD_2Cl_2, 298 K, 126 MHz) of **Rh(*meta*-Me)Cl**. The inset is an enlargement of the resonances between δ 154 and 146 ppm.

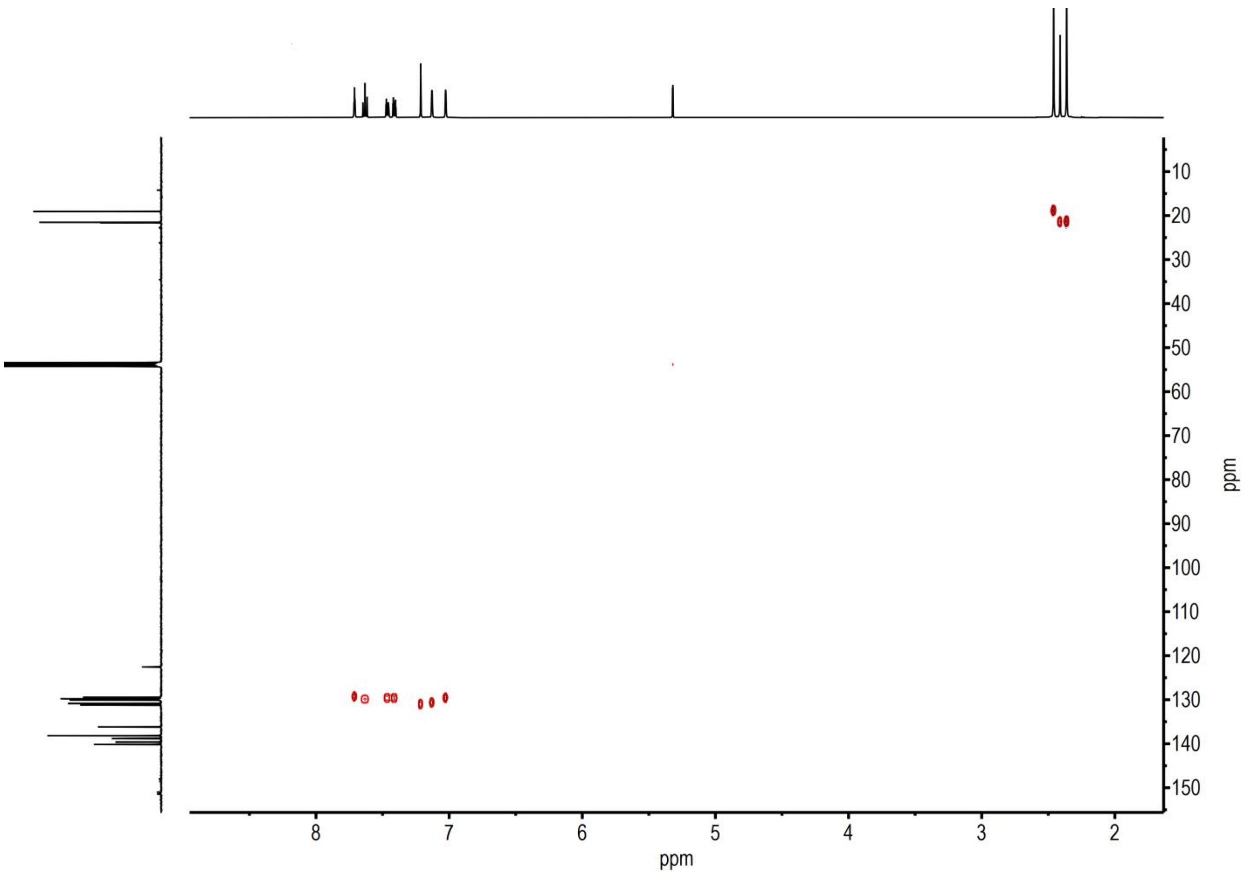

Figure S34: The solution ^{1}H-^{13}C HMQC spectrum (CD_2Cl_2, 298 K) of **Rh(*meta*-Me)Cl**.

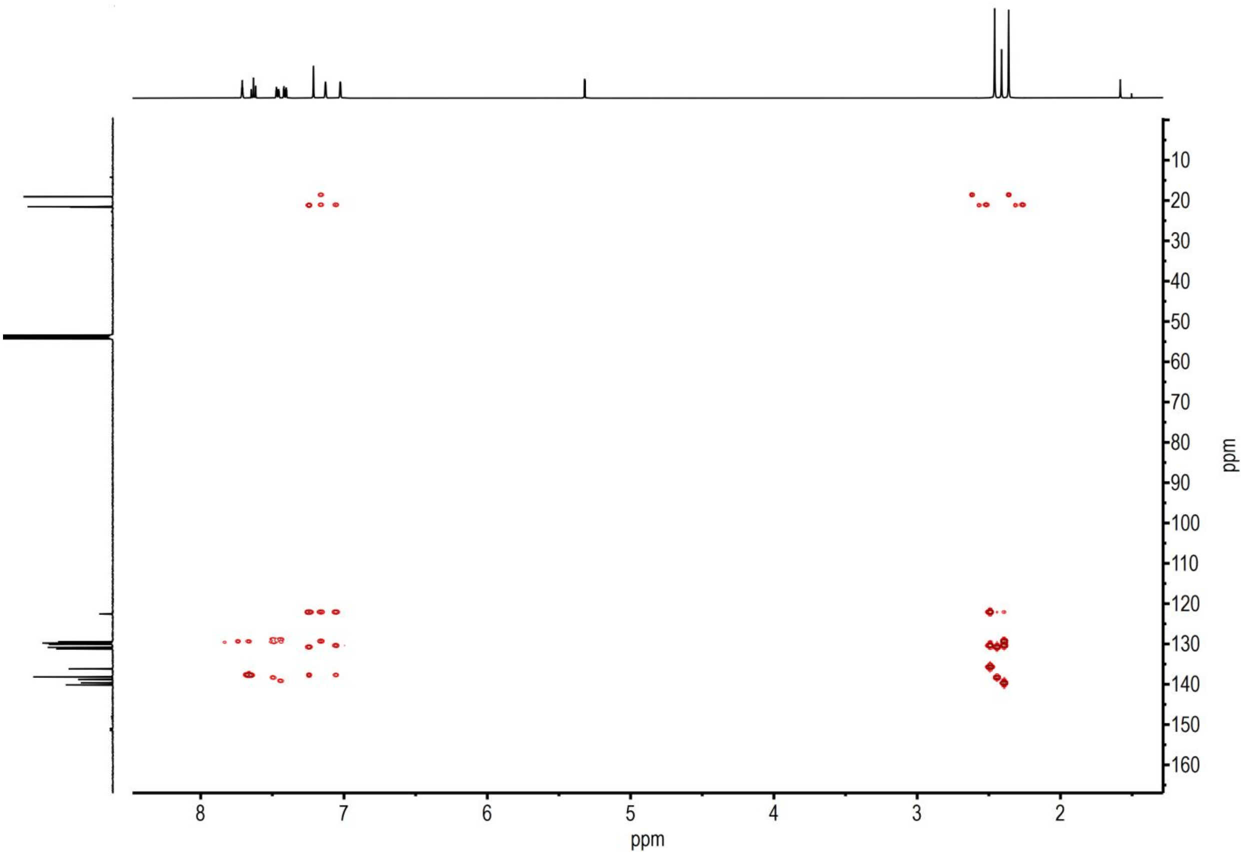

Figure S35: The solution ^{1}H-^{13}C HMBC spectrum (CD_2Cl_2, 298 K) of **Rh(*meta*-Me)Cl**.

S.4.2. [Rh(*meta*-Me)(1-CN)][PF6]

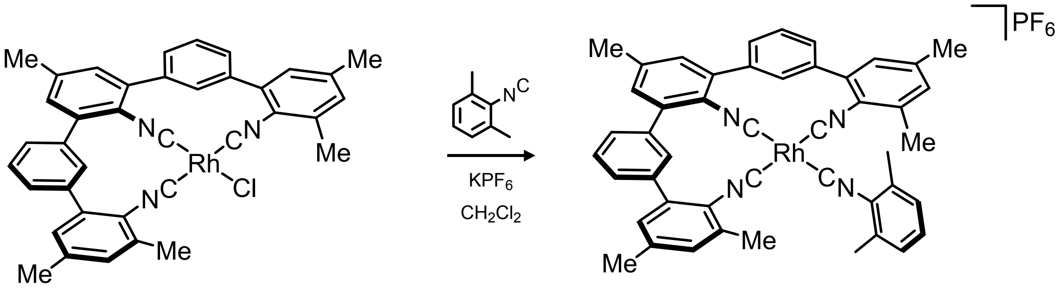

Working using Schlenk line techniques, a 10 mL round bottom flask was charged with **Rh(*meta*-Me)Cl** (100 mg, 150 μmol, 1 *eqv*., M_r: 666), 2,6-dimethylphenyl isocyanide, **1-CN**, (22 mg, 165 μmol, 1.1 *eqv*., M_r: 131) and KPF_6 (55 mg, 300 μmol, 2 *eqv*., M_r: 184). The flask was placed under a N_2 atmosphere and dried and deoxygenated dichloromethane (5 mL) was added. The orange solution was left to stir for 18 hours, which turns pale-yellow in colour.

After this time, and working under air, the solution was filtered into diethyl ether (~20 mL) and left to stir until a permanent precipitate formed. The dark-purple solids were filtered, washed with diethyl ether (2 × 5 mL) then pentane (2 × 5 mL) and dried to yield **[Rh(*meta*-Me)(1-CN)][PF6]** (110 mg, 121 μmol, M_r: 907, 81%) as an air-stable yellow solid.

^{1}H NMR (CD_2Cl_2, 298 K, 500 MHz): δ 7.78 (s, 2H, H_e), 7.73 (app. t., 2H, $^3J_{HH}$ = 7.1 Hz, $^3J_{HH}$ = 7.8 Hz, H_c), 7.56 (d., 2H, $^3J_{HH}$ = 7.1 Hz, H_d), 7.49 (d., 2H, $^3J_{HH}$ = 7.8 Hz H_b), 7.38 (s., 2H, H_a), 7.32 (t., 1H, $^3J_{HH}$ = 7.8 Hz, H_i), 7.21 (br. s., 2H, H_g), 7.19 (d., 2H, $^3J_{HH}$ = 7.8 Hz, H_h), 7.13 (br. s., 2H, H_f), 2.52 (s, 3H, H_m), 2.47 (s, 6H, H_l), 2.44 (s, 6H, H_j), 2.42 (s, 6H, H_k).

^{13}C{^{1}H} NMR (CD_2Cl_2, 298 K, 126 MHz): δ 149.9 (d, ~1C, $^1J_{RhC}$ = 57 Hz, C_1 or C_3), 146.9 (d, ~2C, $^1J_{RhC}$ = 62 Hz, C_2), 146.1 (d, ~1C, $^1J_{RhC}$ = 55 Hz, C_1 or C_3), 142.3, 141.8, 140.0, 140.0, 138.0, 137.3, 136.2, 135.9, 131.4 (C_b), 131.2 (C_n), 130.6 (C_t), 130.6 (C_g), 130.5 (C_f), 130.5 (C_L), 130.3 (C_h), 129.5 (C_j), 128.7 (C_s), 126.5, 121.8, 119.6, 21.8 (C_y), 21.6 (C_x), 19.1 (C_v), 19.0 (C_w).

^{19}F{^{1}H} NMR (CD_2Cl_2, 298 K, 470 MHz): δ -71.0 (d., J_{PF} = 710 Hz).

^{31}P{^{1}H} NMR (CD_2Cl_2, 298 K, 202 MHz): δ -144.0 (septet, J_{PF} = 710 zHz).

ESI-MS (calc. for $[Rh(C_{38}H_{29}N_3)(C_9H_9N_1)]^+$): *m/z* 761.2152 (761.2146).

Elemental Analysis (calc. for $[Rh(C_{38}H_{29}N_3)(C_9H_9N_1)][PF_6] \cdot 0.5H_2O$): C 61.75 (61.65), H 4.20 (4.29), N 6.18 (6.12).

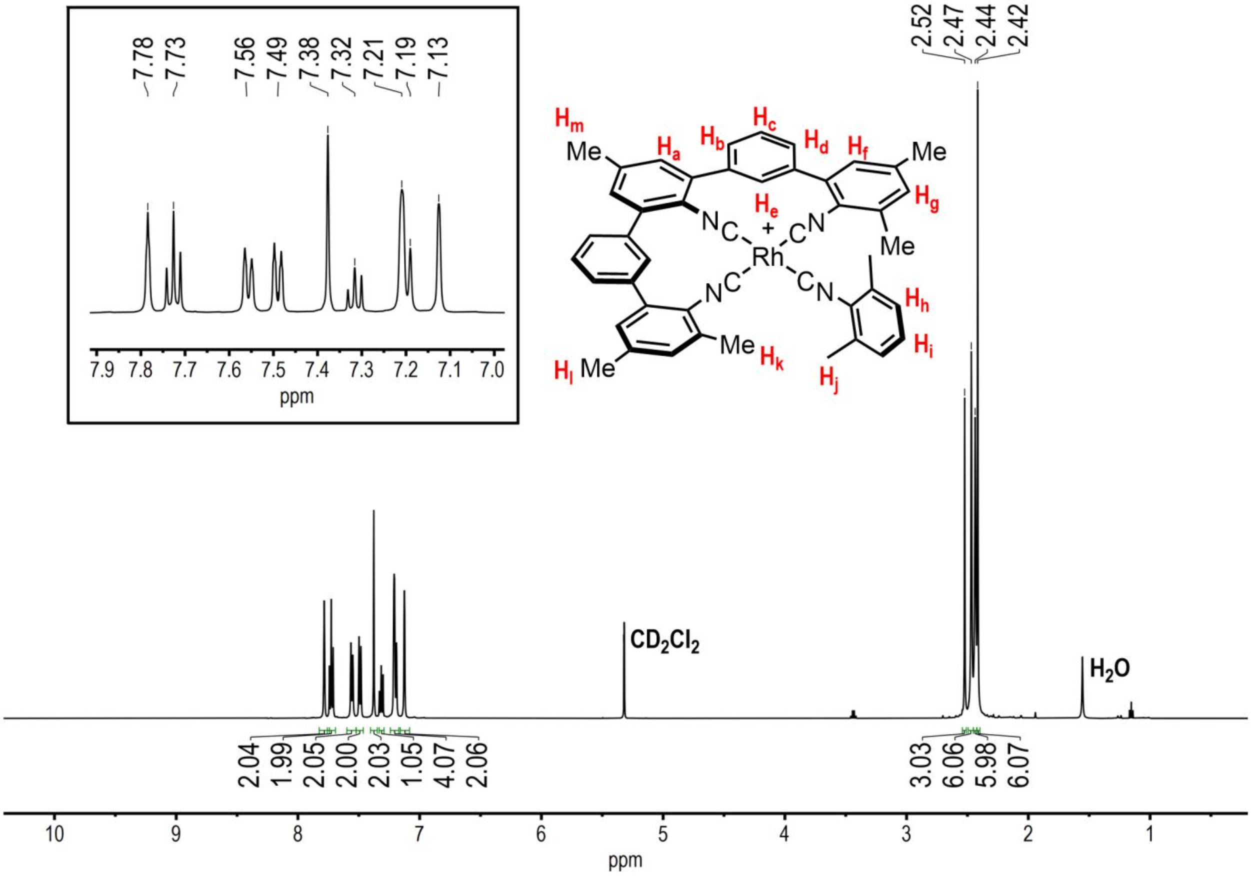

Figure S36: The solution ^{1}H NMR spectrum (CD_2Cl_2, 298 K, 500 MHz) of **[Rh(*meta*-Me)(1-CN)][PF$_6$]**. The inset is an enlargement of the resonances between δ 7.9 and 7.0 ppm.

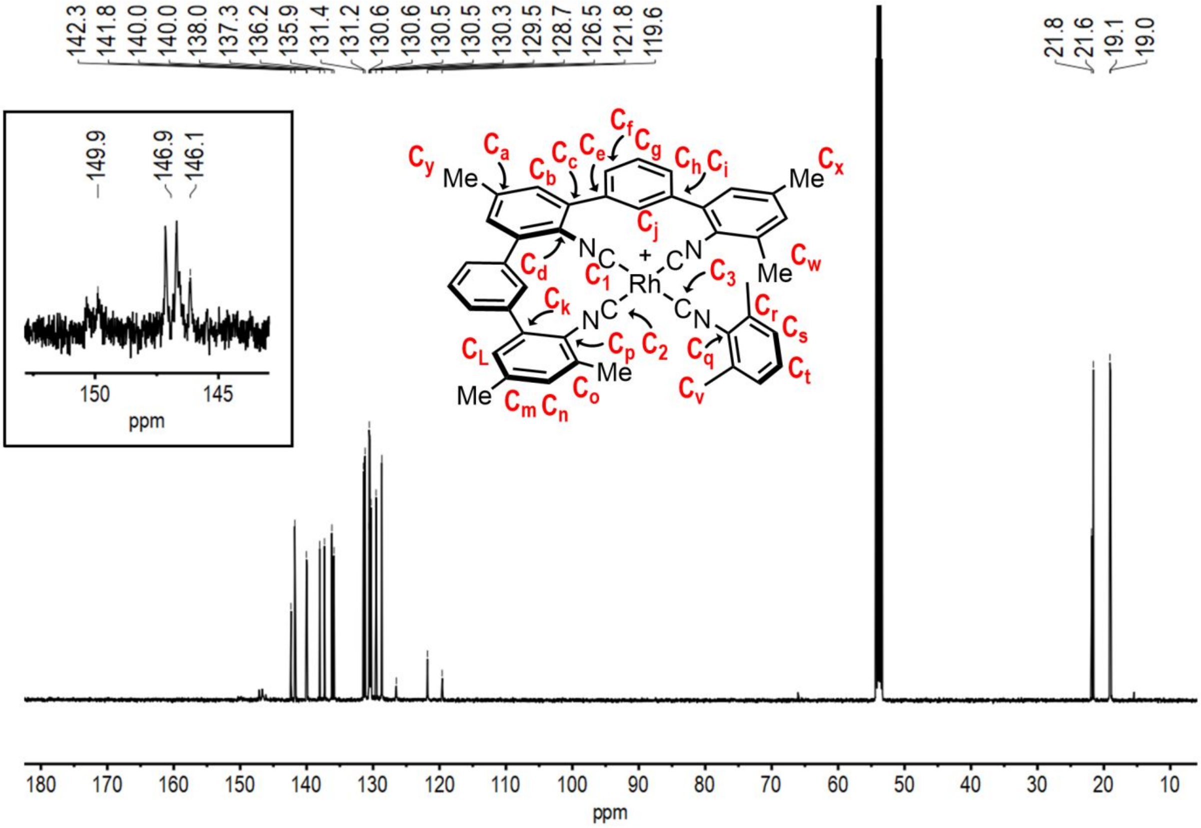

Figure S37: The solution $^{13}C\{^{1}H\}$ NMR spectrum (CD_2Cl_2, 298 K, 126 MHz) of **[Rh(*meta*-Me)(1-CN)][PF$_6$]**. The inset is an enlargement of the resonances between δ 152 and 143 ppm.

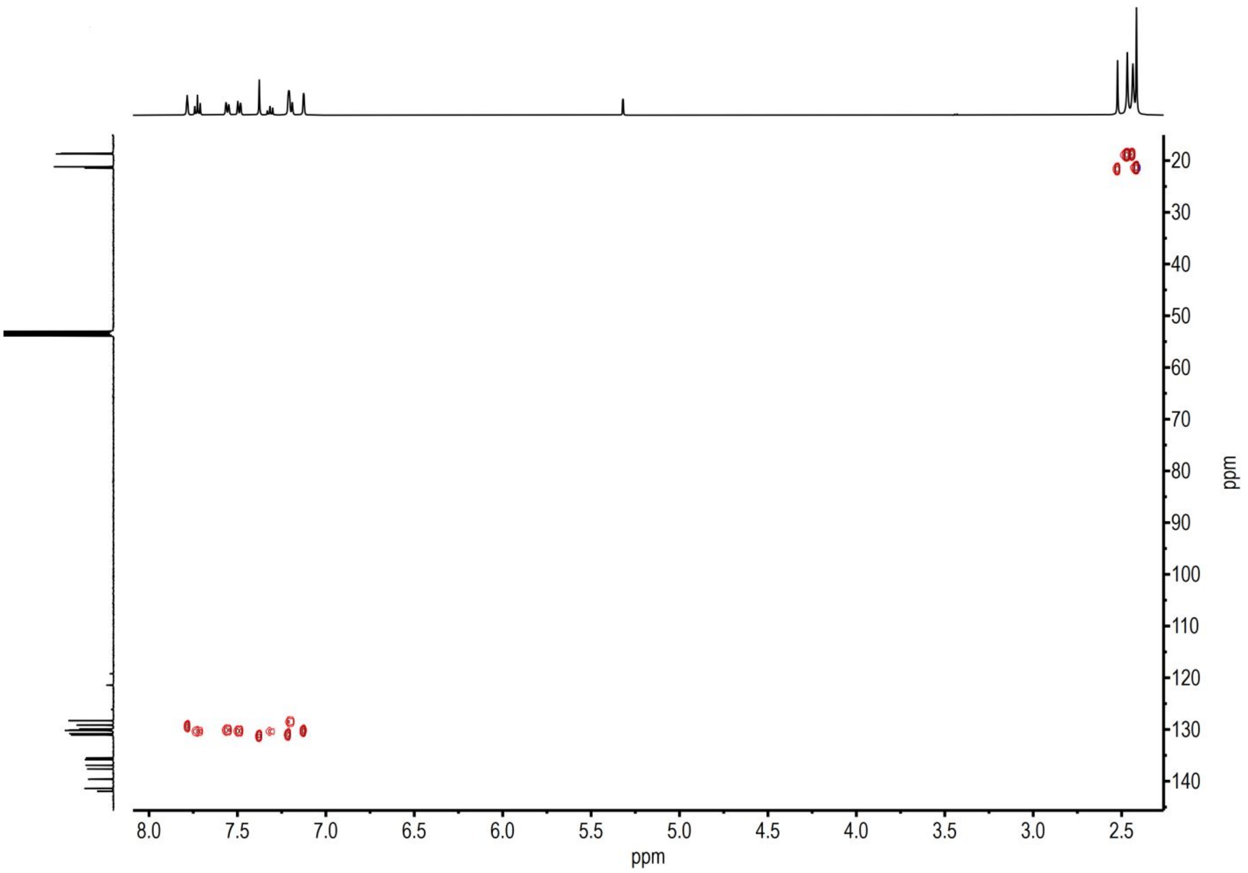

Figure S38: The solution ^{1}H-^{13}C HMQC spectrum (CD_2Cl_2, 298 K) of **[Rh(*meta*-Me)(1-CN)][PF$_6$]**.

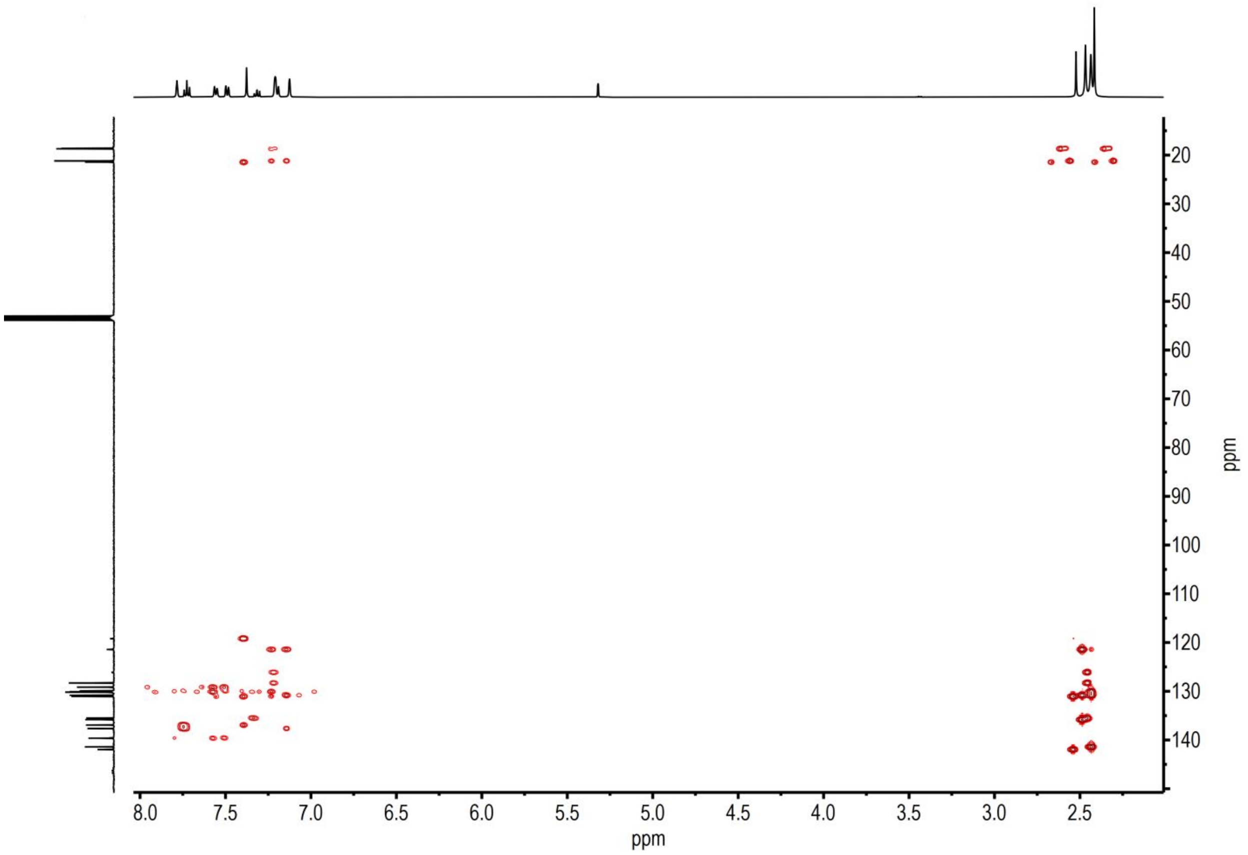

Figure S39: The solution ^{1}H-^{13}C HMBC spectrum (CD_2Cl_2, 298 K) of **[Rh(*meta*-Me)(1-CN)][PF$_6$]**.

S.4.3. [(Rh(***meta***-Me))$_2$(2-CN)][PF$_6$]$_2$

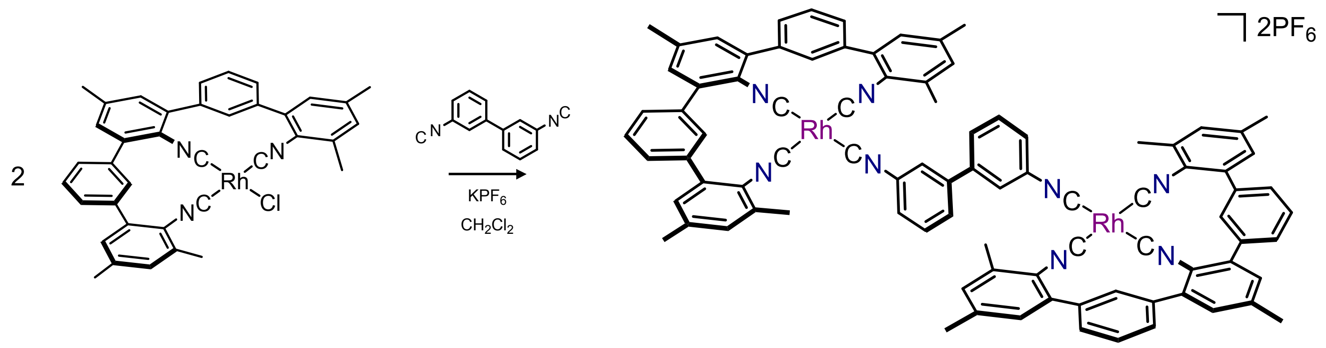

Working using Schlenk line techniques, a 10 mL round bottom flask was charged with **Rh(*meta*-Me)Cl** (100 mg, 150 µmol, 2.05 *eqv*., M_r: 666), **2-CN** (15 mg, 73.2 µmol, 1 *eqv*., M_r: 204) and KPF_6 (54 mg, 293 µmol, 4 *eqv*., M_r: 184). The flask was placed under a N_2 atmosphere and dried and deoxygenated dichloromethane (5 mL) was added. The orange solution was left to stir for 18 hours, which turns dark red in colour.

After this time, and working under air, the solution was filtered into diethyl ether (~20 mL) and left to stir until a permanent precipitate has formed. The dark-red solids were filtered, washed with diethyl ether (2 × 5 mL) then pentane (2 × 5 mL) and dried to yield **[(Rh(*meta*-Me))$_2$(2-CN)][PF$_6$]$_2$** (82 mg, 45.6 µmol, M_r: 1799, 62%) as an air-stable purple solid.

^{1}H NMR (CD_2Cl_2, 298 K, 250 µM, 500 MHz): δ 7.79 (s., 4H), 7.72 (m., 6H), 7.65 (m., 4H), 7.55 (d., 4H), 7.48 (m., 6H), 7.38 (s., 4H), 7.19 (s., 4H), 7.12 (s., 4H), 2.52 (s., 6H), 2.47 (s., 12H), 2.40 (s., 12H).

^{19}F{^{1}H} NMR (CD_2Cl_2, 298 K, 250 µM, 470 MHz): δ -71.0 (d., J_{PF} = 710 Hz).

^{31}P{^{1}H} NMR (CD_2Cl_2, 298 K, 250 µM, 202 MHz): δ -144.0 (septet, J_{PF} = 710 Hz).

ESI-MS (calc. for $[(Rh(C_{38}H_{29}N_3))_2(C_{14}H_8N_2)]^{2+}$): *m/z* 732.1765 (732.1755).

Elemental Analysis (calc. for $[(Rh(C_{38}H_{29}N_3))_2(C_{14}H_8N_2)][PF_6]_2\cdot H_2O\cdot 0.9KPF_6$): C 55.87 (55.75), H 3.87 (3.54), N 5.82 (5.78).

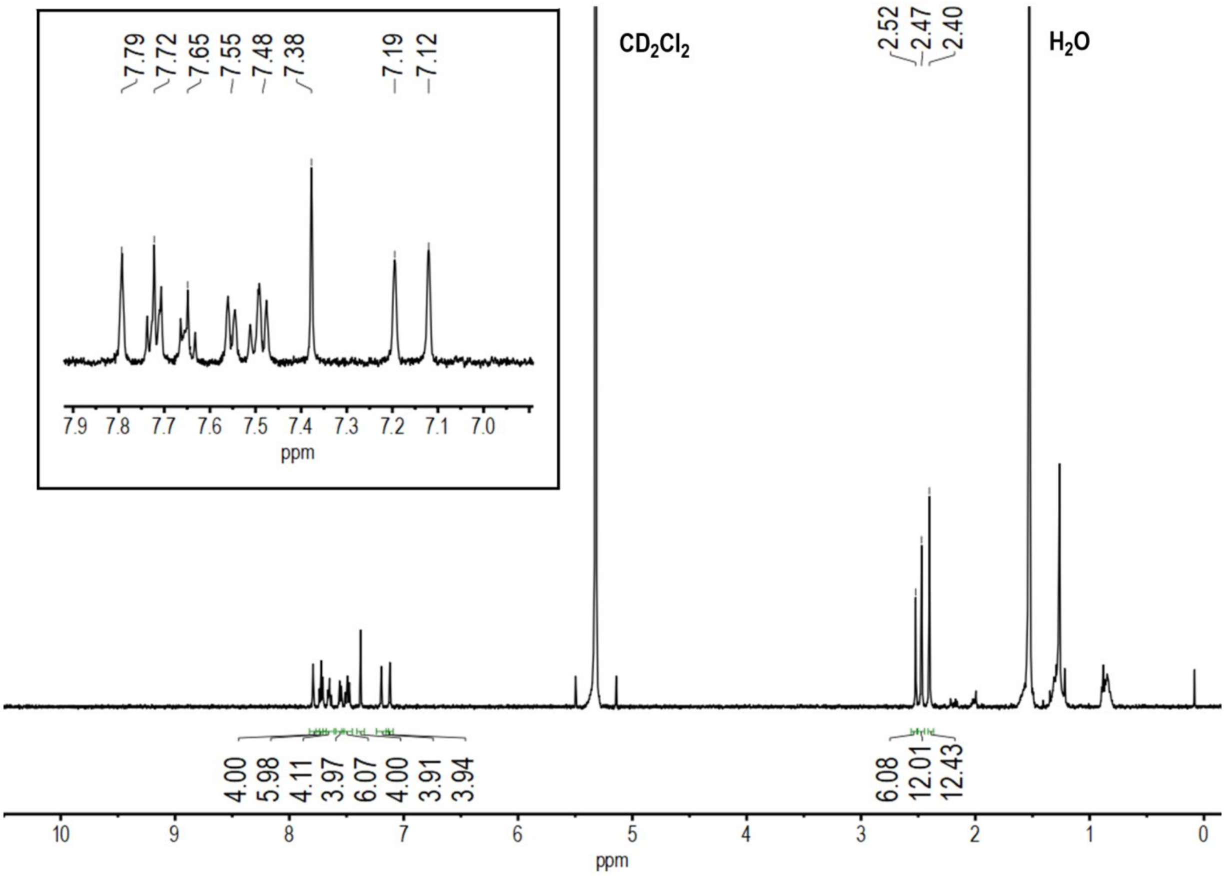

Figure S40: The solution ^{1}H NMR spectrum (CD_2Cl_2, 298 K, 250 μM, 500 MHz) of **[(Rh(*meta*-Me))$_2$(2-**

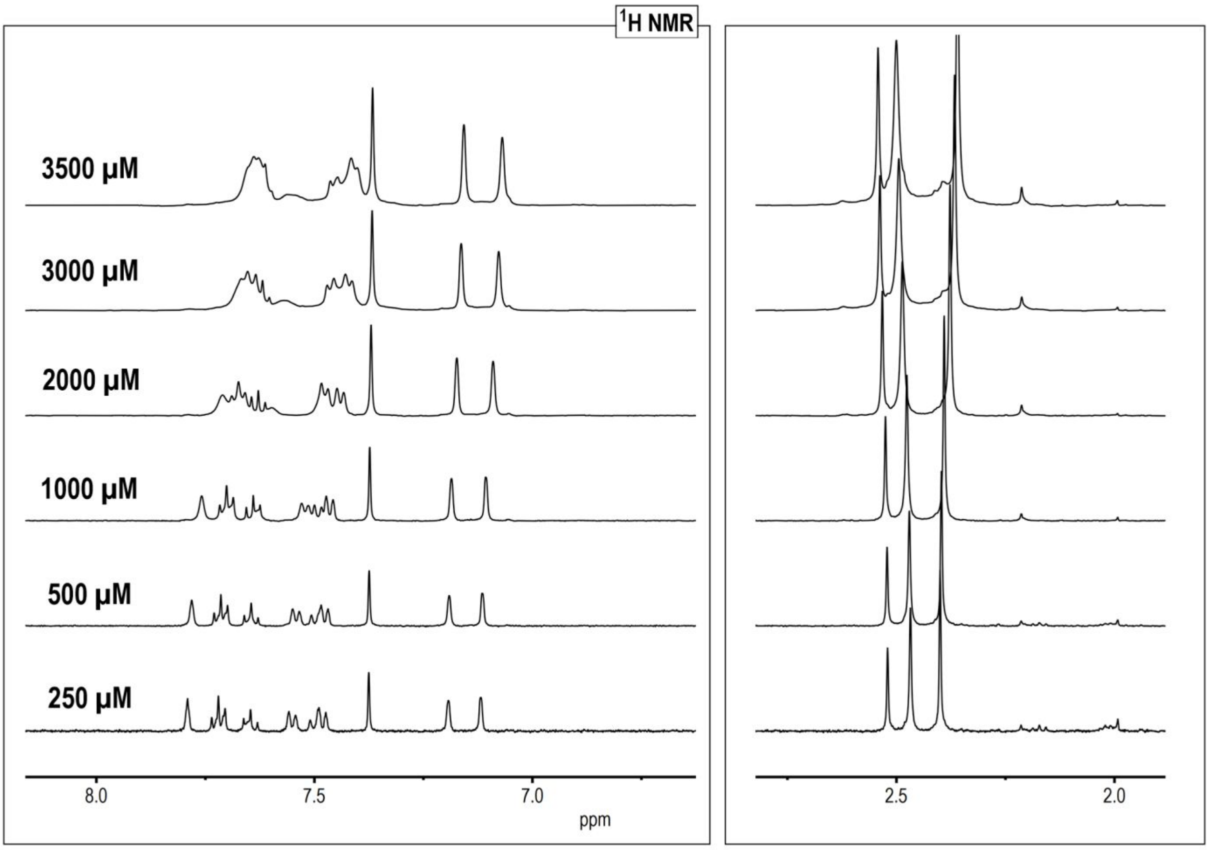

CN)][PF6]$_2$. The inset is an enlargement of the resonances between δ 7.9 and 6.9 ppm.

Figure S41: Concentration dependence upon the solution ^{1}H NMR spectrum (CD_2Cl_2, 298 K, 500 MHz) of **[(Rh(*meta*-Me))$_2$(2-CN)][PF$_6$]$_2$**.

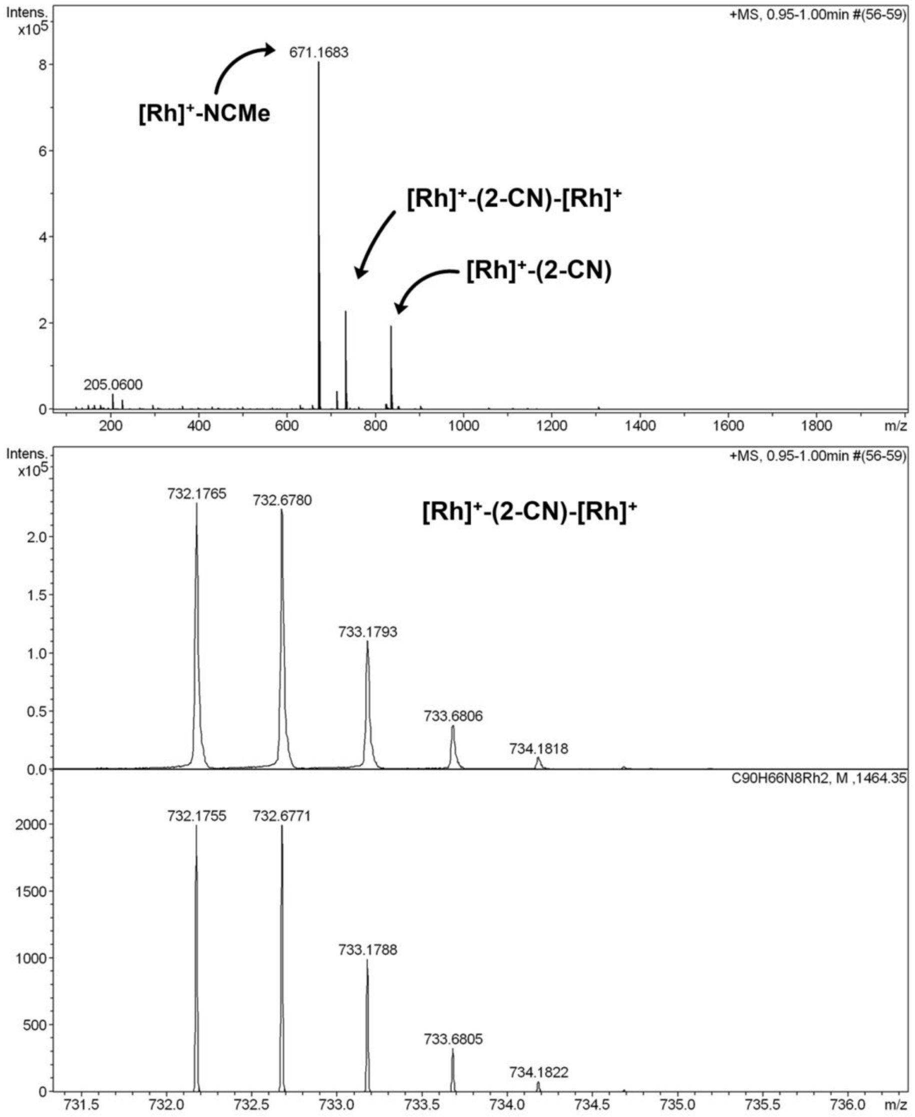

Figure S42: Experimental (top & middle) and calculated (bottom) high resolution ESI-MS of **[(Rh(*meta*-Me))$_2$(2-CN)][PF$_6$]$_2$**.

S.4.4. Rh(*para*-Me)Cl

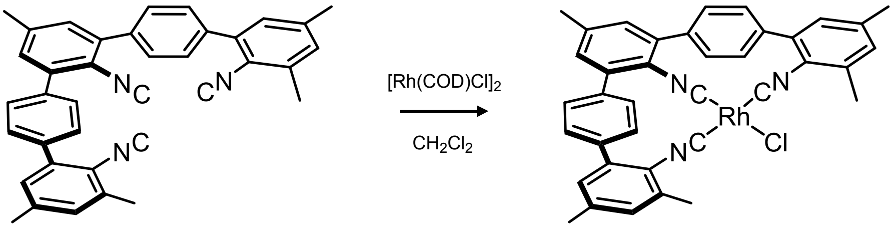

Working using Schlenk line techniques, a 10 mL round bottom flask was charged with ***para*-Me** (93 mg, 175 µmol, 2 *eqv*., M_r: 528) and $[Rh(COD)Cl]_2$ (43 mg, 87.7 µmol, 1 *eqv*., M_r: 493). The flask was placed under a N_2 atmosphere and dried and deoxygenated dichloromethane (5 mL) was added. The yellow solution was left to stir for 15 minutes, which turns intense purple in colour.

After this time, and working under air, the solution was filtered and triturated with pentane (~20 mL). The purple solids were filtered and washed with diethyl ether (3 × 5 mL) then pentane (3 × 5 mL) and dried to yield **Rh(*para*-Me)Cl** (96 mg, 144 µmol, M_r: 666, 82%) as an air-stable purple solid.

^{1}H NMR (CD_2Cl_2, 298 K, 500 MHz): δ 7.7 – 6.8 (br. m., 15H), 2.5 – 2.2 (m., 15H).

ESI-MS (calc. for $[Rh(C_{41}H_{35}N_3)(CH_3CN)]^+$): *m/z* 713.2140 (713.2146).

Elemental Analysis (calc. for $Rh(C_{38}H_{29}N_3)Cl{\cdot}2H_2O$): C 65.18 (65.01), H 4.69 (4.74), N 5.94 (5.99).

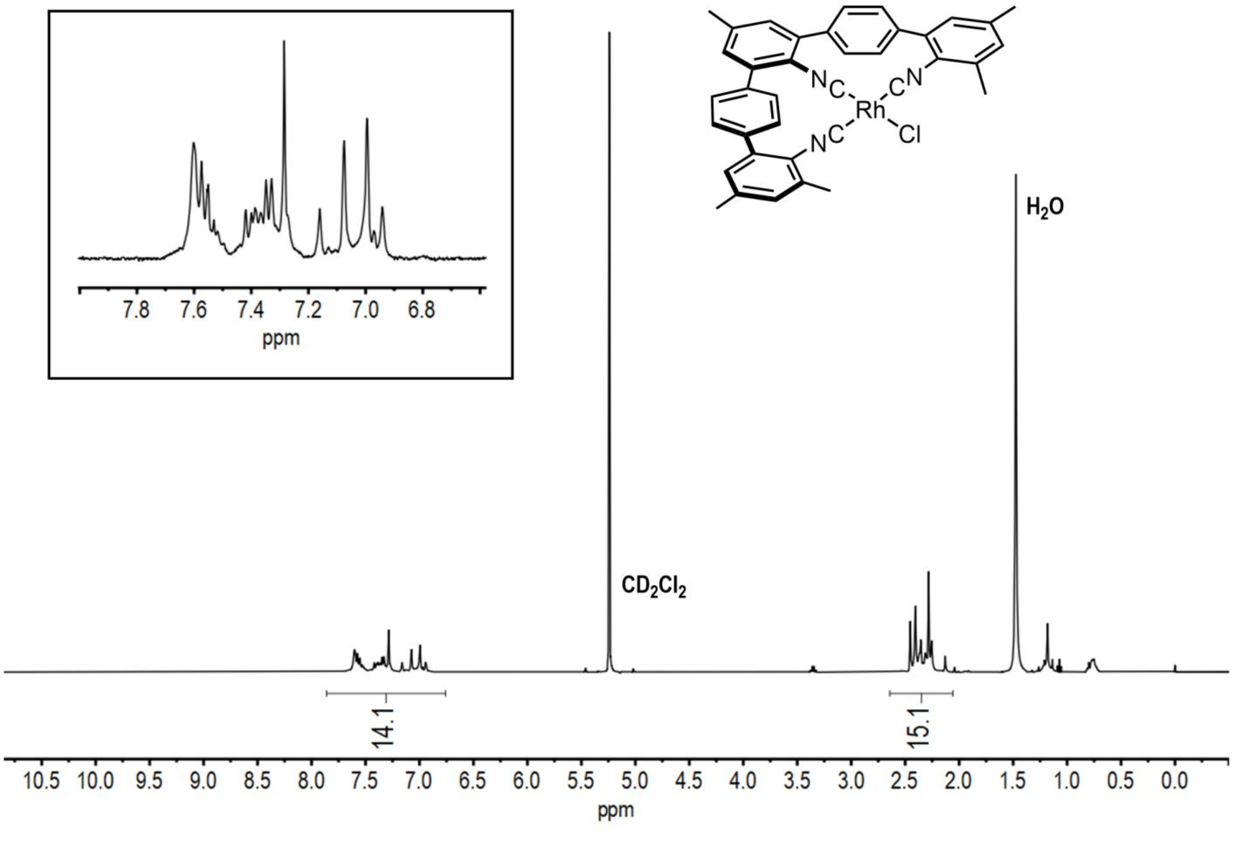

Figure S43: The solution ^{1}H NMR spectrum (CD_2Cl_2, 298 K, 500 MHz) of **Rh(*para*-Me)Cl**. The inset is an enlargement of the resonances between δ 7.8 and 6.8 ppm.

S.3.5. Rh(*para*-^{t}Bu)Cl

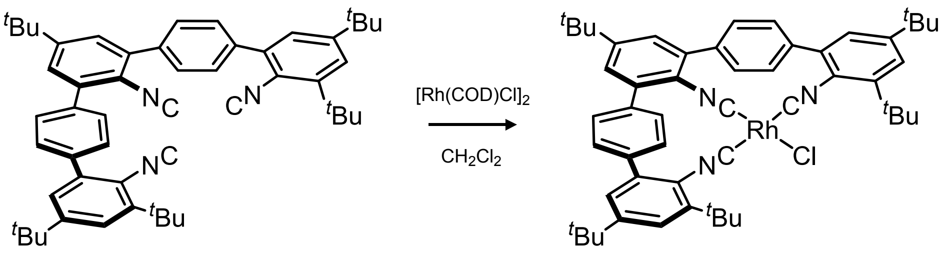

Working using Schlenk line techniques, a 10 mL round bottom flask was charged with ***para*-^{t}Bu** (100 mg, 136 μmol, 2 *eqv*., M_r: 738) and [Rh(COD)Cl]$_2$ (34 mg, 67.8 μmol, 1 *eqv*., M_r: 493). The flask was placed under a N_2 atmosphere and dried and deoxygenated dichloromethane (5 mL) was added. The yellow solution was left to stir for 15 minutes, which turns dark yellow in colour.

After this time, and working under air, the solution was filtered and filtrate cooled to 0°C. Pentane (~20 mL) added and solution left to stir until a permanent precipitate formed. The yellow solids were filtered, washed with 0°C pentane (2 × 2 mL) and dried to yield **Rh(*para*-^{t}Bu)Cl** (72 mg, 136 μmol, M_r: 876, 61%) as an air-stable yellow solid.

Note: Single crystals suitable for single crystal X-ray diffraction of **Rh(*para*-^{t}Bu)Cl** were grown from a saturated v/v 95: 5 dichloromethane: acetonitrile solution of left at room temperature.

^{1}H NMR (CD_2Cl_2, 298 K, 500 MHz): δ 7.63 (s, 2H, H_a), 7.48 (d, 2H, $^4J_{HH}$ = 2.2 Hz, H_e), 7.44 (d, 2H, $^4J_{HH}$ = 2.2 Hz, H_d), 7.40 (m, 8H, H_b and H_c), 1.50 (s, 18H, H_g), 1.47 (s, 9H, H_h), 1.37 (s, 18H, H_f).

NOESY ^{1}H NMR (CD_2Cl_2, 298 K, 500 MHz): Cross-peak between δ 7.63 with 1.47, 7.48 with 1.50 and 1.37, 7.44 with 1.37 ppm.

^{13}C{^{1}H} NMR (**CD_2Cl_2, 298 K, 126 MHz**): δ 155.4 (d, 1C, $^1J_{RhC}$ = 56 Hz, C_u)*, 152.4, 151.1, 149.8 (d, 2C, $^1J_{RhC}$ = 70 Hz, C_t)*, 147.4, 142.6, 139.6, 139.3, 138.9, 129.7 (C_e or C_f), 129.3 (C_e or C_f), 129.0, 124.0, 123.9 (C_b), 123.7 (C_k), 123.6 (C_i), 36.1, 35.6, 31.5, 31.3 (C_o), 31.3 (C_q), 30.0 (C_s).

* = coupling constants did not change when ^{13}C{^{1}H} NMR experiment conducted at 101 MHz.

ESI-MS (calc. for [Rh($C_{53}H_{59}N_3$)(CH_3CN)]$^+$): *m/z* 881.4035 (881.4024).

Elemental Analysis (calc. for Rh($C_{53}H_{59}N_3$)Cl_1): C 72.26 (72.63), H 6.70 (6.79), N 4.69 (4.79).

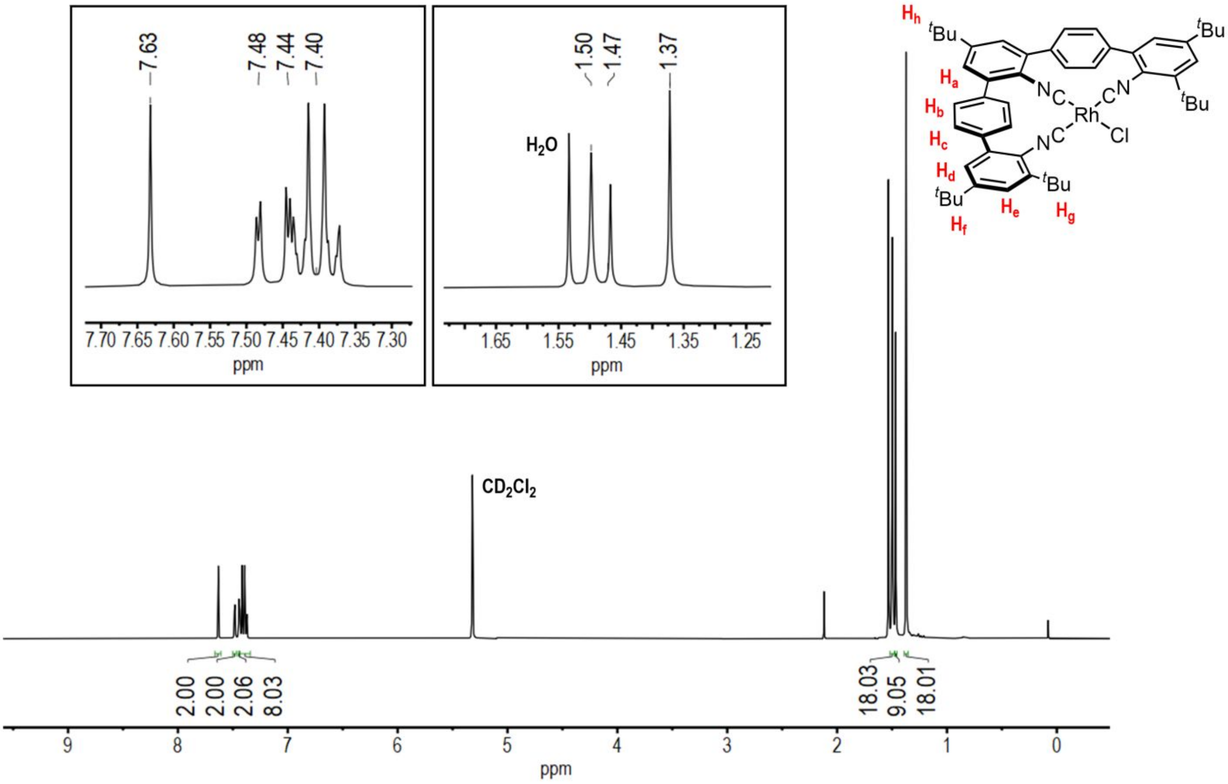

Figure S44: The solution ^{1}H NMR spectrum (CD_2Cl_2, 298 K, 500 MHz) of **Rh(*para*-^{t}Bu)Cl**. The inset is an enlargement of the resonances between δ 7.7 and 7.3 ppm and between 1.7 and 1.2 ppm.

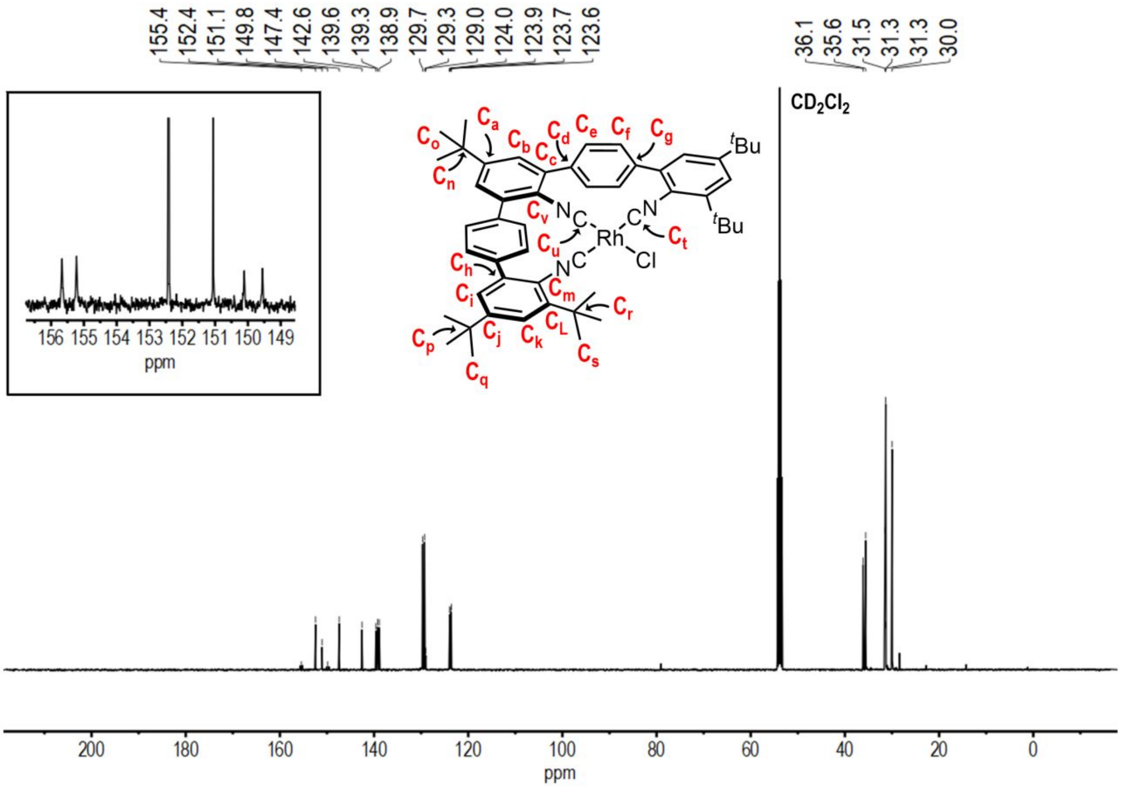

Figure S45: The solution $^{13}C\{^{1}H\}$ NMR spectrum (CD_2Cl_2, 298 K, 125 MHz) of **Rh(*para*-^{t}Bu)Cl**. The inset is an enlargement of the resonances between δ 156 and 149 ppm.

Figure S46: The solution ^{1}H-^{13}C HMQC spectrum (CD_2Cl_2, 298 K) of **Rh(*para*-^{t}Bu)Cl**.

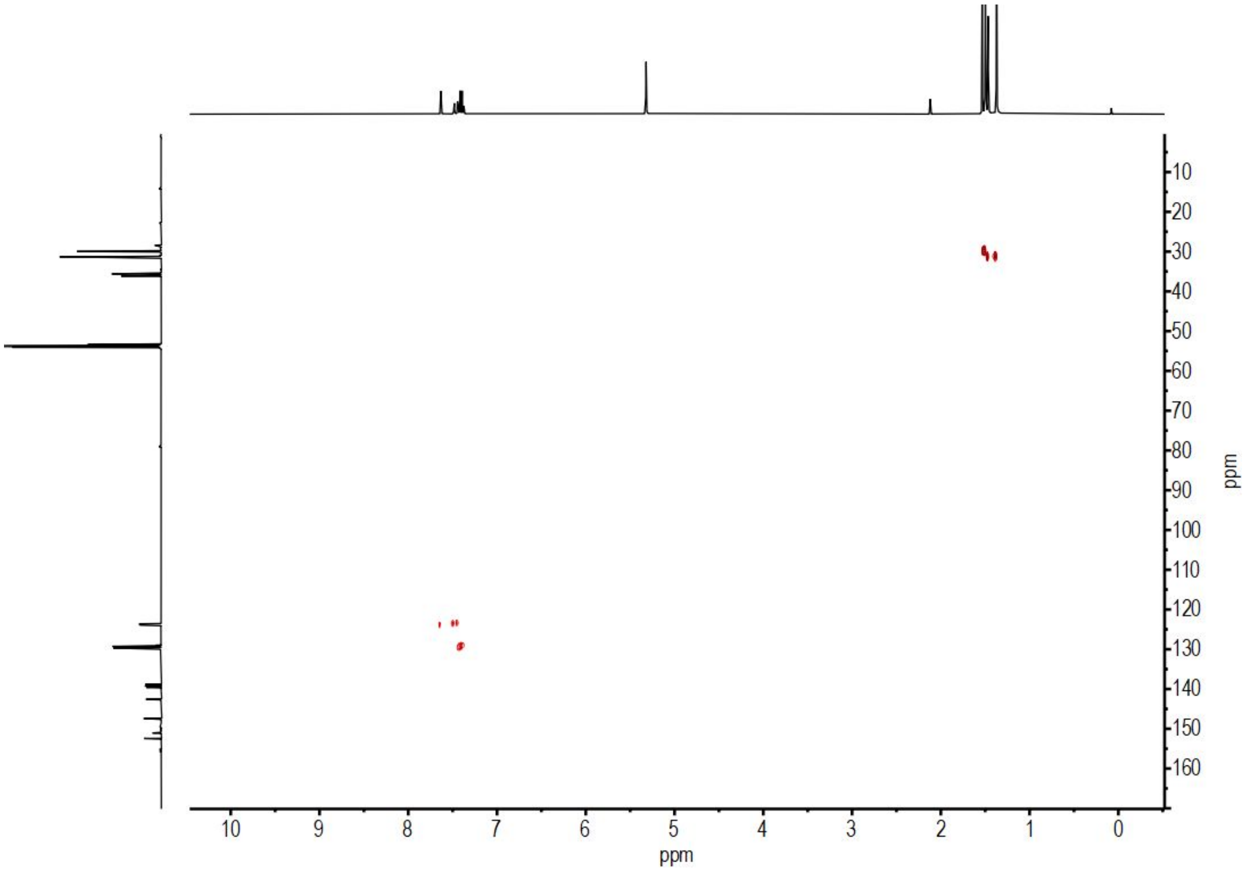

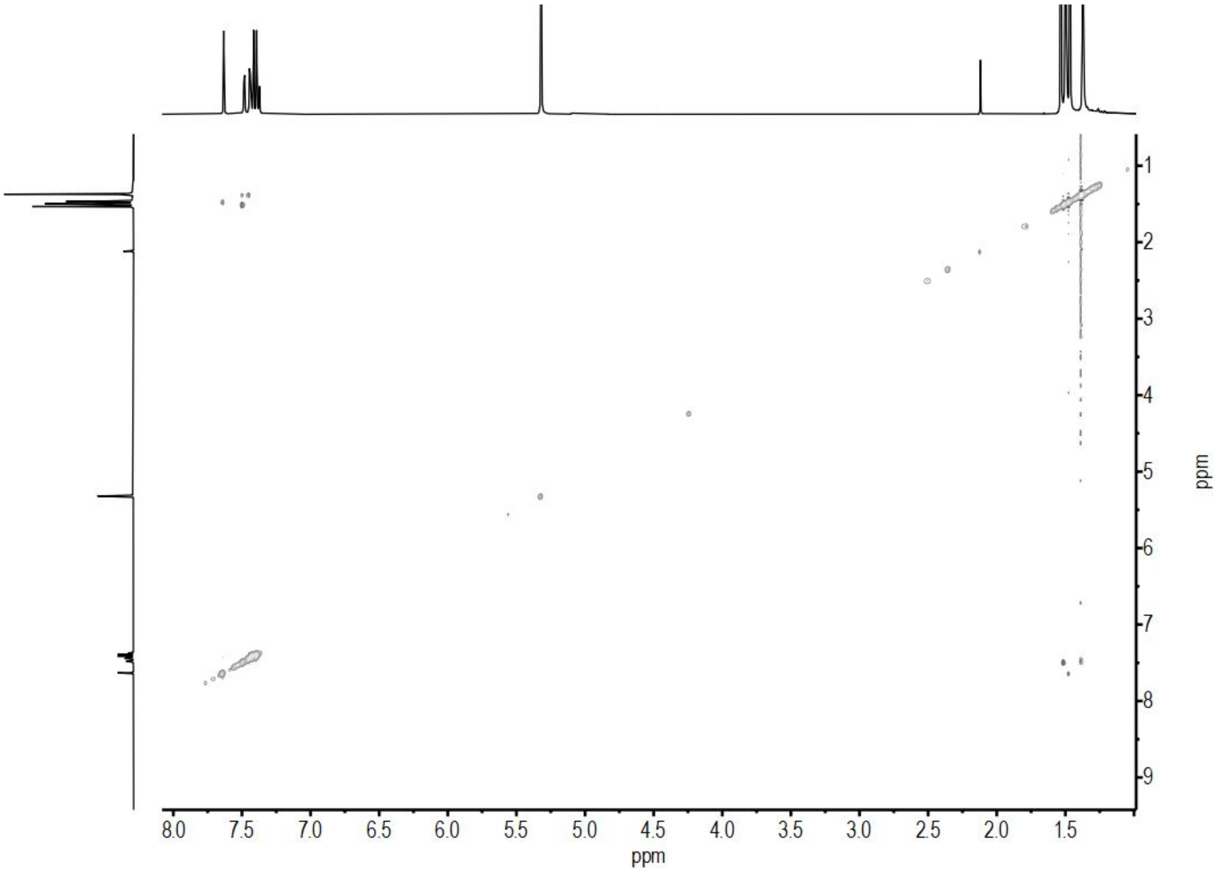

Figure S47: The solution ^{1}H-^{1}H NOESY spectrum (CD_2Cl_2, 298 K, 500 MHz) of **Rh(*para*-^{t}Bu)Cl**.

S.5. Optical Spectroscopy

S.5.1. Rh(*meta*-Me)Cl

S.5.1.1. UV-Vis Spectra

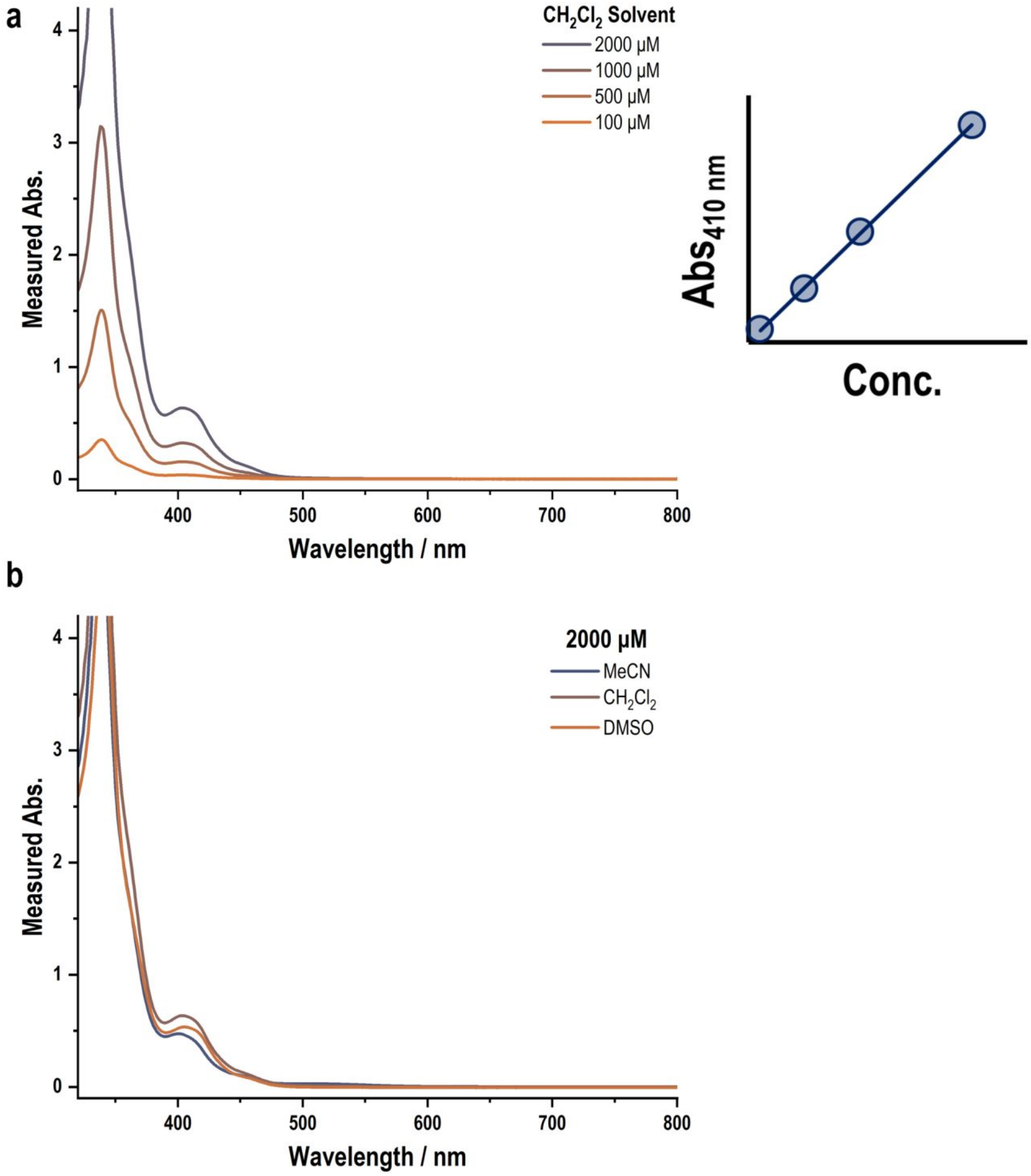

Figure S48: UV-Vis absorption spectra (293 K) for **Rh(*meta*-Me)Cl** in a) CH_2Cl_2 solvent (2000 → 100 μM, CH_2Cl_2) (with inset illustrating the relationship with Beer-Lambert law at 410 nm) and b) in MeCN, CH_2Cl_2 and DMSO solvent at 2000 μM.

S.5.2. [Rh(*meta*-Me)(1-CN)][PF_6]

S.5.2.1. UV-Vis Spectra

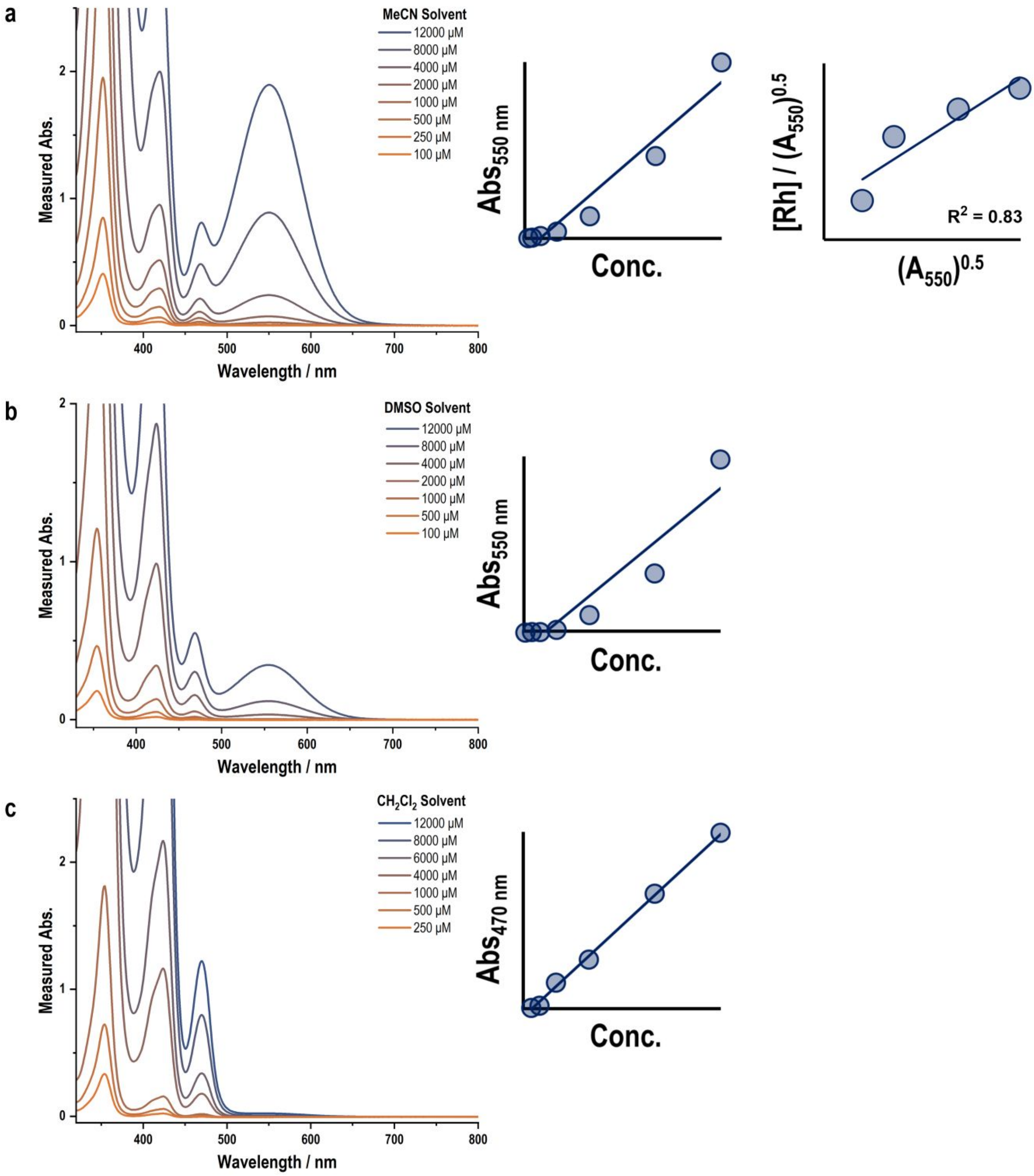

Figure S49: UV-Vis absorption spectra (293 K) with insets showing accompanying Beer-Lambert plot for **[Rh(*meta*-Me)(1-CN)][PF_6]** in a) MeCN (deviation from the Beer-Lambert law at 550 nm; with linear fitting of dimeric absorptions using a monomer−dimer equilibrium model) b) DMSO (deviation from the Beer-Lambert law at 550 nm) and c) CH_2Cl_2 solvent (Beer-Lambert law relationship at 470 nm).

S.5.2.2. Emisson Spectra

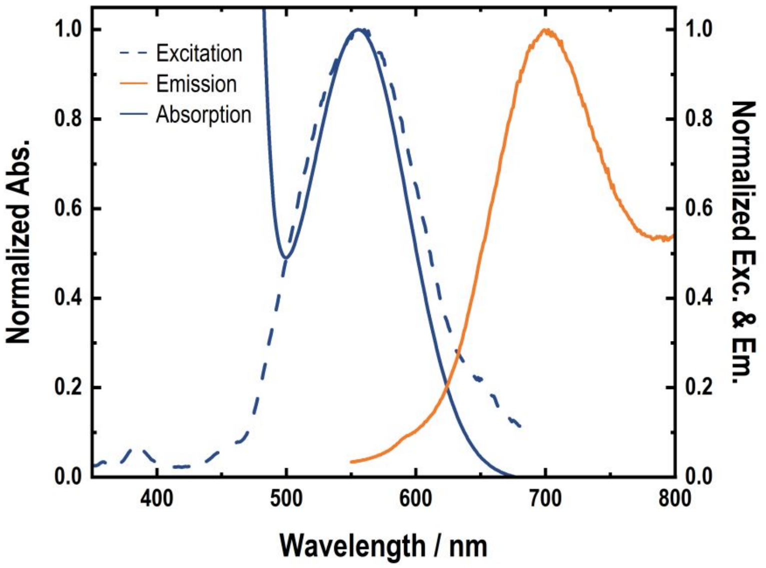

Figure S50: UV-Vis absorption (blue, solid), excitation (blue, dashed) and fluorescence emission (orange, solid) traces (2 mM, 293 K, deaerated DMSO) of **[Rh(*meta*-Me)(1-CN)][PF$_6$]** following excitation at 532 nm.

S.5.2.3. Transient Absorption Measurements

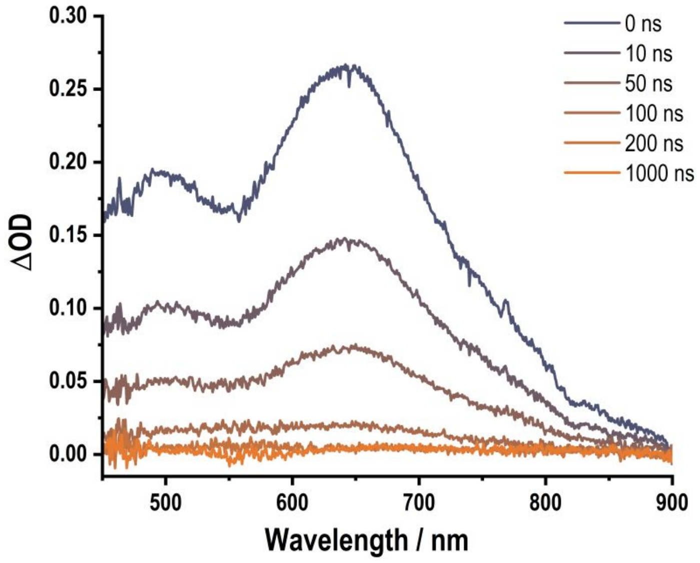

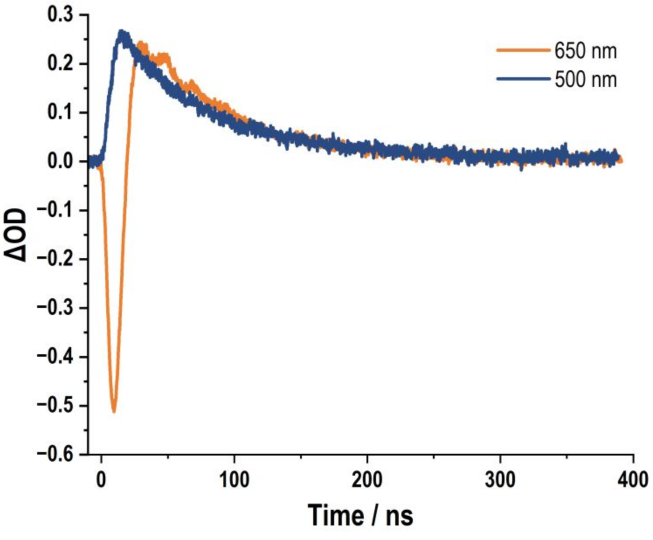

Figure S51: Left: UV−vis transient absorption spectrum (4 mM, degassed DMSO, 293 K) of **[Rh(*meta*-Me)(1-CN)][PF$_6$]**, integrated over 50 ns after indicated delay times. Right: UV−vis transient absorption decays of the ESA signals of **[Rh(*meta*-Me)(1-CN)][PF$_6$]** at two different wavelengths. All traces recorded after excitation with a 532 nm pulsed laser (7 mJ / pulse, 10 Hz, pulse width ~10 ns).

S.5.2.4. Time-Resolved Emission

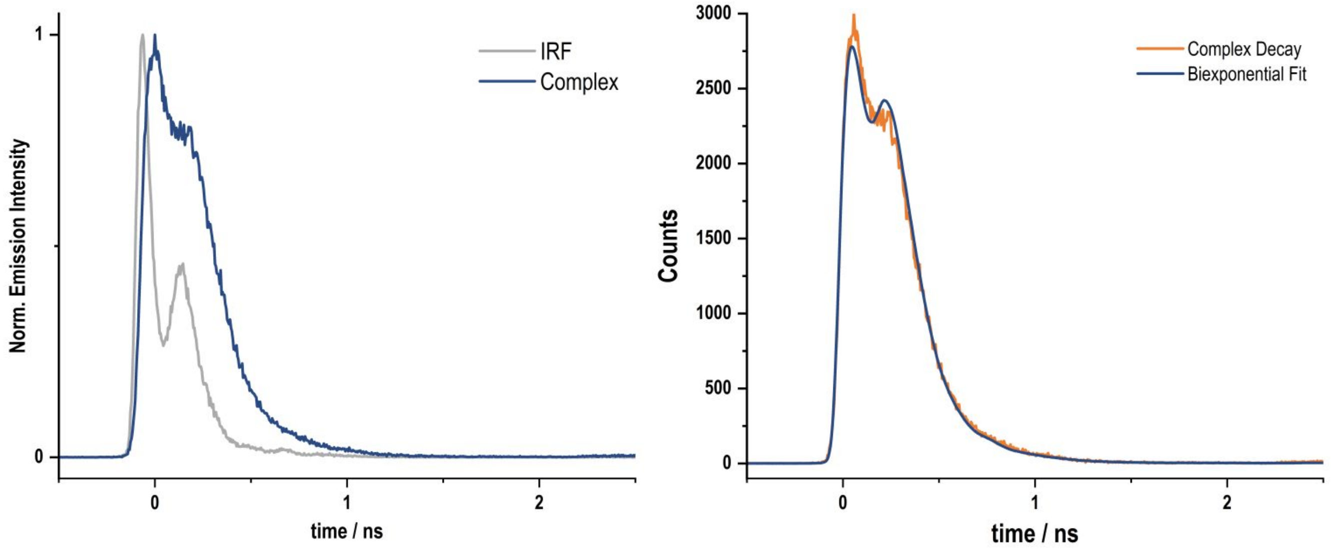

Figure S52: TCSPC data of **[Rh(*meta*-Me)(1-CN][PF$_6$]** (2000 µM, degassed DMSO, 293 K) recorded after excitation at 635 nm. Left: Normalized decay trace (blue) with instrument's response function (IRF, pulse width ca. 65 ps) in pure solvent is plotted as reference (gray trace). Right: Decay trace (orange) with biexponential fit (blue trace). This biexponential fit yields a dominant (99.9%) time constant of τ = 112 ps and a minor (0.1%) time constant of τ = 1084 ps. The former is assigned to dimers; the latter is tentatively assigned to higher order oligomers.

S.5.3. [(Rh(meta-Me))$_2$(2-CN)][PF$_6$]$_2$

S.5.3.1. UV-Vis Spectra

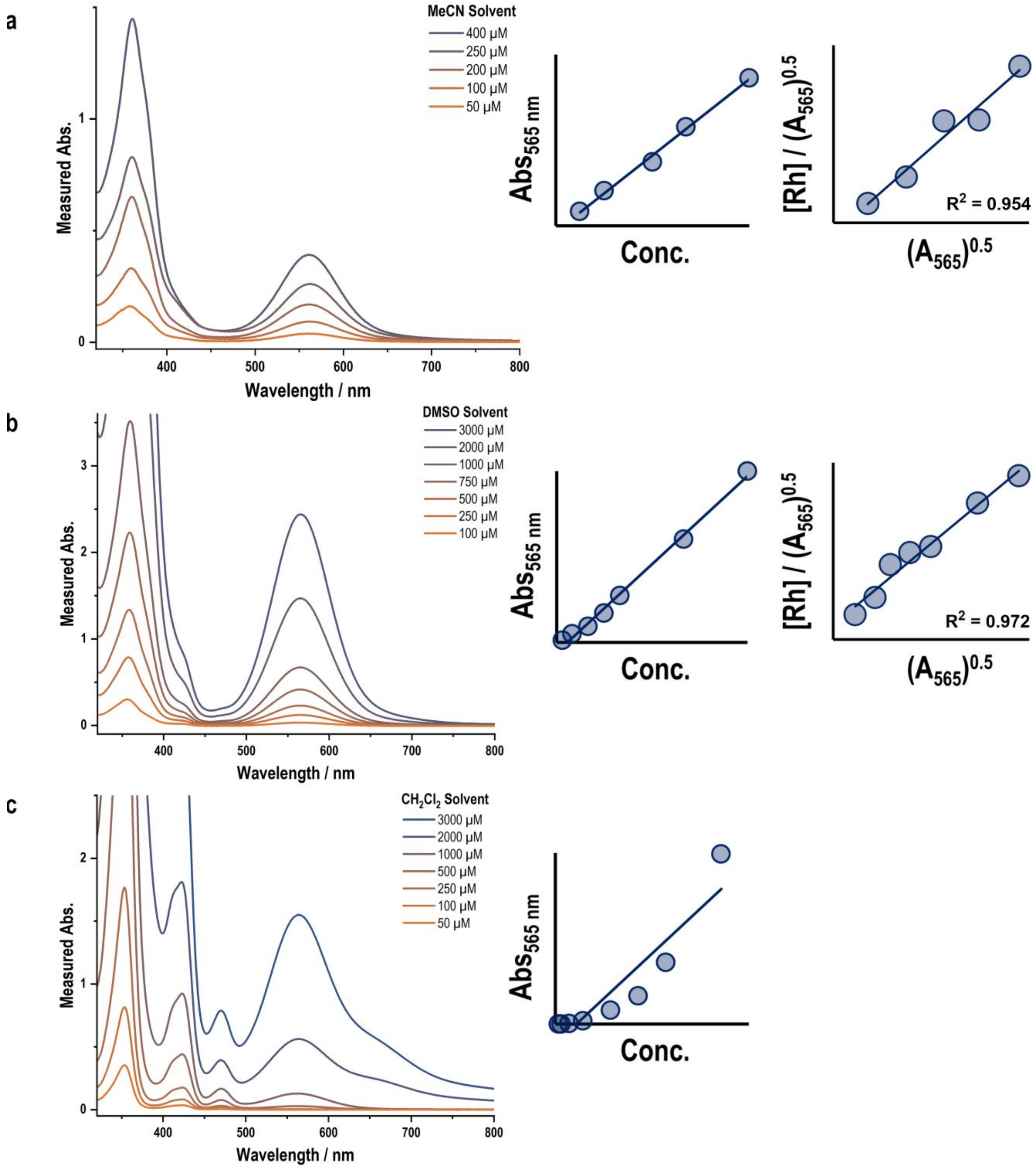

Figure S53: UV-Vis steady state absorption spectra (293 K) with insets showing Beer-Lambert plot for **[(Rh(*meta*-Me))$_2$(2-CN)][PF$_6$]$_2$** in a) MeCN (Beer-Lambert law relationship at 565 nm; linear fitting of dimeric absorptions using a monomer−dimer equilibrium model), b) DMSO (Beer-Lambert law relationship at 565 nm; linear fitting of dimeric absorptions using a monomer−dimer equilibrium model) and c) CH_2Cl_2 solvent (deviation from the Beer-Lambert law at 565 nm).

S.5.3.2. Emisson Spectra

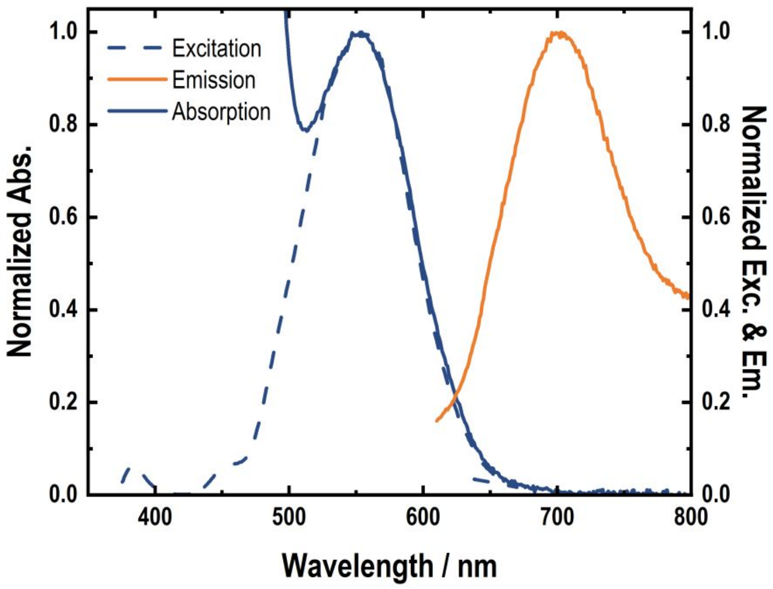

Figure S54: UV-Vis absorption (blue, solid), excitation (blue, dashed) and fluorescence emission (orange, solid) traces (300 µM, 293 K, DMSO) of **[(Rh(*meta*-Me))$_2$(2-CN)][PF$_6$]$_2$** following excitation at 532 nm.

S.5.3.3. Transient Absorption Measurements

Figure S55: Left: UV−vis transient absorption spectra (250 µM, degassed DMSO, 293 K) of **[(Rh(*meta*-**

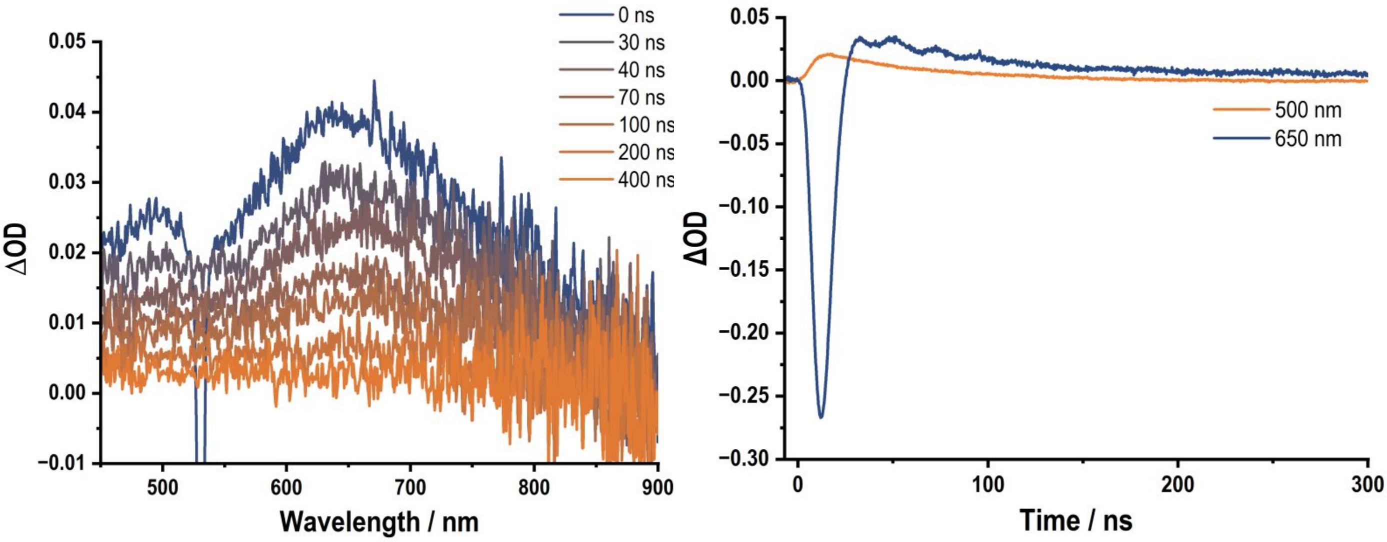

Me))$_2$(2-CN)][PF$_6$]$_2$, integrated over 50 ns after indicated delay times. Right: UV−vis transient absorption time traces of the ESA signal of **[(Rh(*meta*-Me))$_2$(2-CN)][PF$_6$]$_2$** at two different wavelengths. All traces recorded after excitation with a 532 nm pulsed laser (7 mJ / pulse, 10 Hz, pulse width ~10 ns).

S.5.3.4. Time-Resolved Emission

Figure S56: TCSPC spectra of **[(Rh(*meta*-Me))$_2$(2-CN)][PF$_6$]$_2$** (500 µM, degassed DMSO, 293 K)

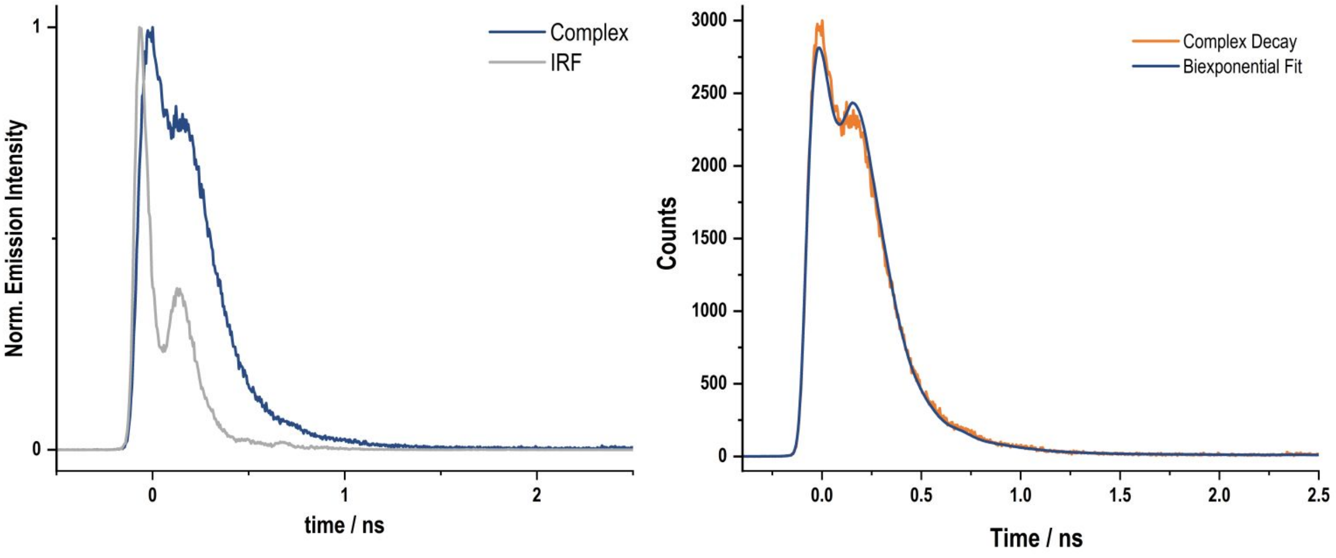

recorded after excitation at 635 nm. Left: Normalized decay trace (blue) with instrument’s response function (IRF, pulse width ca. 65 ps) in pure solvent is plotted as reference (gray trace). Right: Decay trace (orange) with biexponential fit (blue trace). This biexponential fit yields a dominant (99.6%) time constant of τ = 109 ps and a minor (0.4%) time constant of τ = 1446 ps. The former is assigned to dimers; the latter is tentatively assigned to higher order oligomers.

S.5.4. Rh(*para*-Me)Cl

S.5.4.1. UV-Vis Spectra

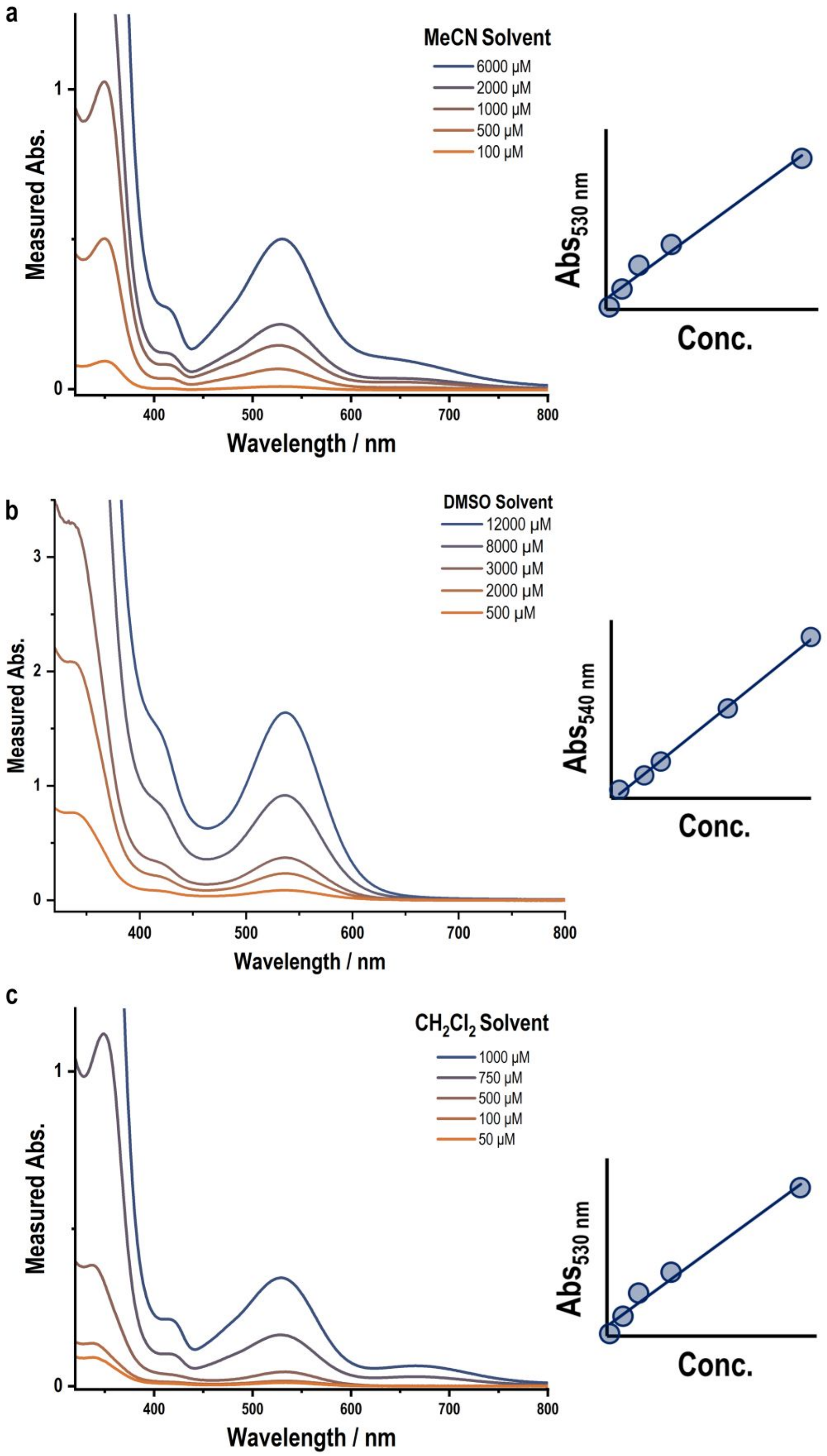

Figure S57: UV-Vis absorption spectra (293 K) with insets showing Beer-Lambert plot for **Rh(*para*-Me)Cl** in a) MeCN b) DMSO (Beer-Lambert law relationship at 540 nm) and c) CH_2Cl_2 (deviation from the Beer-Lambert law at 540 nm) solvent.

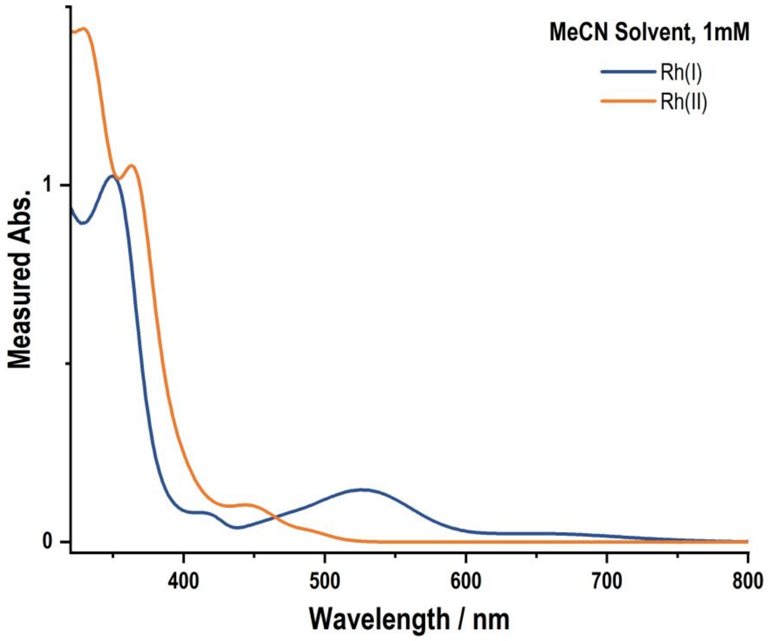

Figure S58: UV-Vis absorption spectra (293 K) of **Rh(*para*-Me)Cl** before (*Rh(I)* – blue trace) and after (*Rh(II)* – orange trace) HCl addition.

S.5.4.2. Emisson Spectra

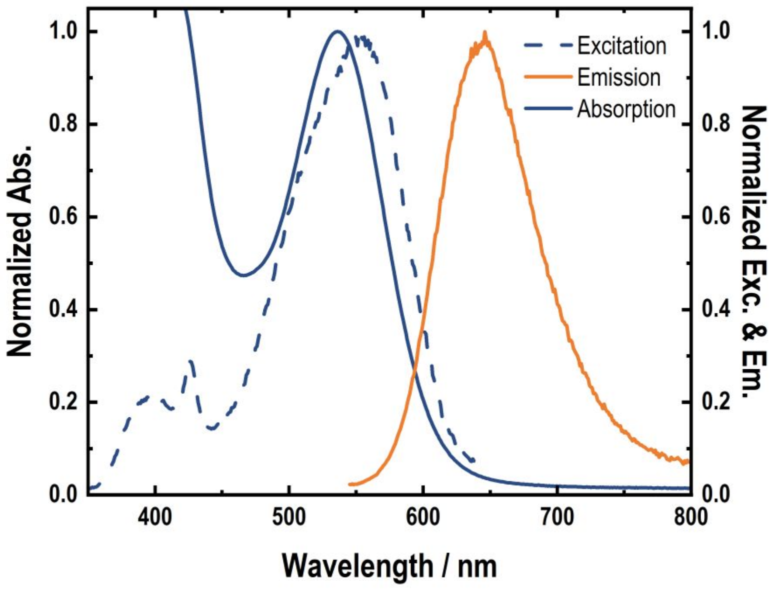

Figure S59: UV-Vis absorption (blue, solid), excitation (blue, dashed) and emission (orange, solid) traces (300 μM, 293 K, deaerated DMSO) of **Rh(*para*-Me)Cl** following excitation at 532 nm.

S.5.4.3. Transient Absorption Measurements

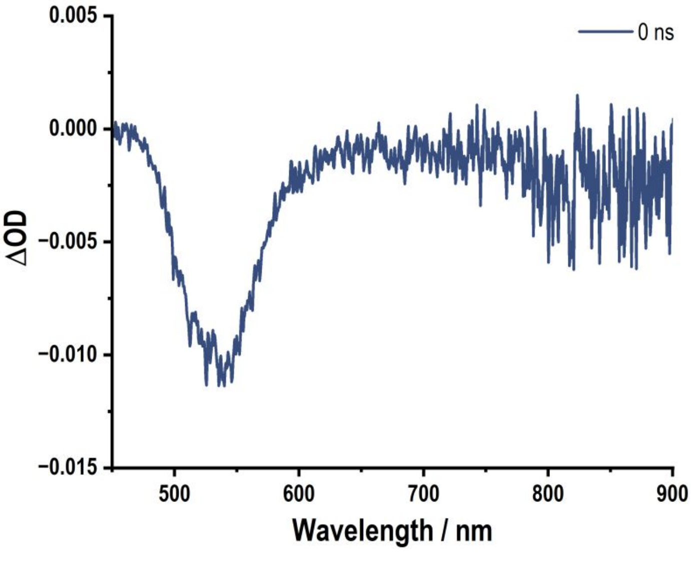

Figure S60: UV−vis transient absorption spectrum (300 μM, degassed DMSO, 293 K) of **Rh(*para*-Me)Cl**, integrated over 50 ns. Traces recorded after excitation with a 532 nm pulsed laser (7 mJ / pulse, 10 Hz, pulse width ~10 ns). The absence of an excited-state absorption signal indicates no measurable triplet-state formation. The observed bleach of ground-state absorption could reflect a photochemical reaction.

S.5.4.4. Time-Resolved Emission

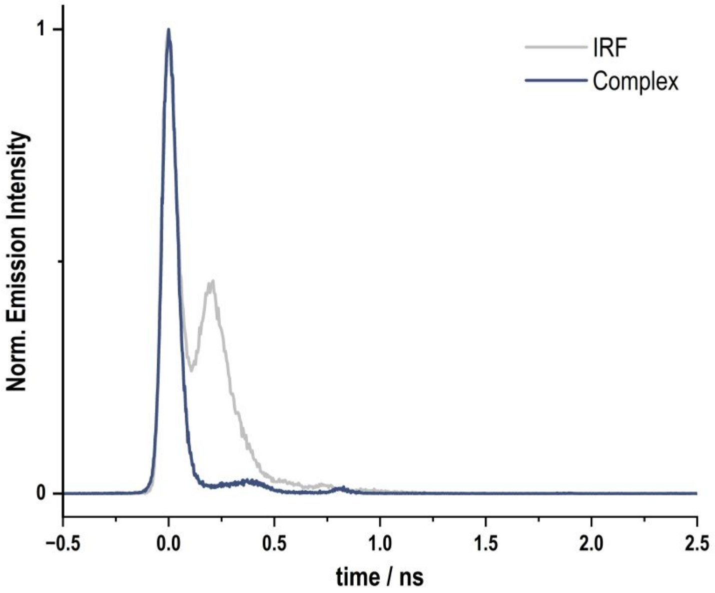

Figure S61: Normalized TCSPC spectra of **Rh(*para*-Me)Cl** (500 µM, degassed DMSO, 293 K) recorded after excitation at 635 nm. The instrument's response function (IRF, pulse width ca. 65 ps) in pure solvent is plotted as reference (gray trace). The fluorescence decay in this case is instrumentally limited.

S.5.5. Rh(*para*-*t*Bu)Cl

S.5.5.1. UV-Vis Spectra

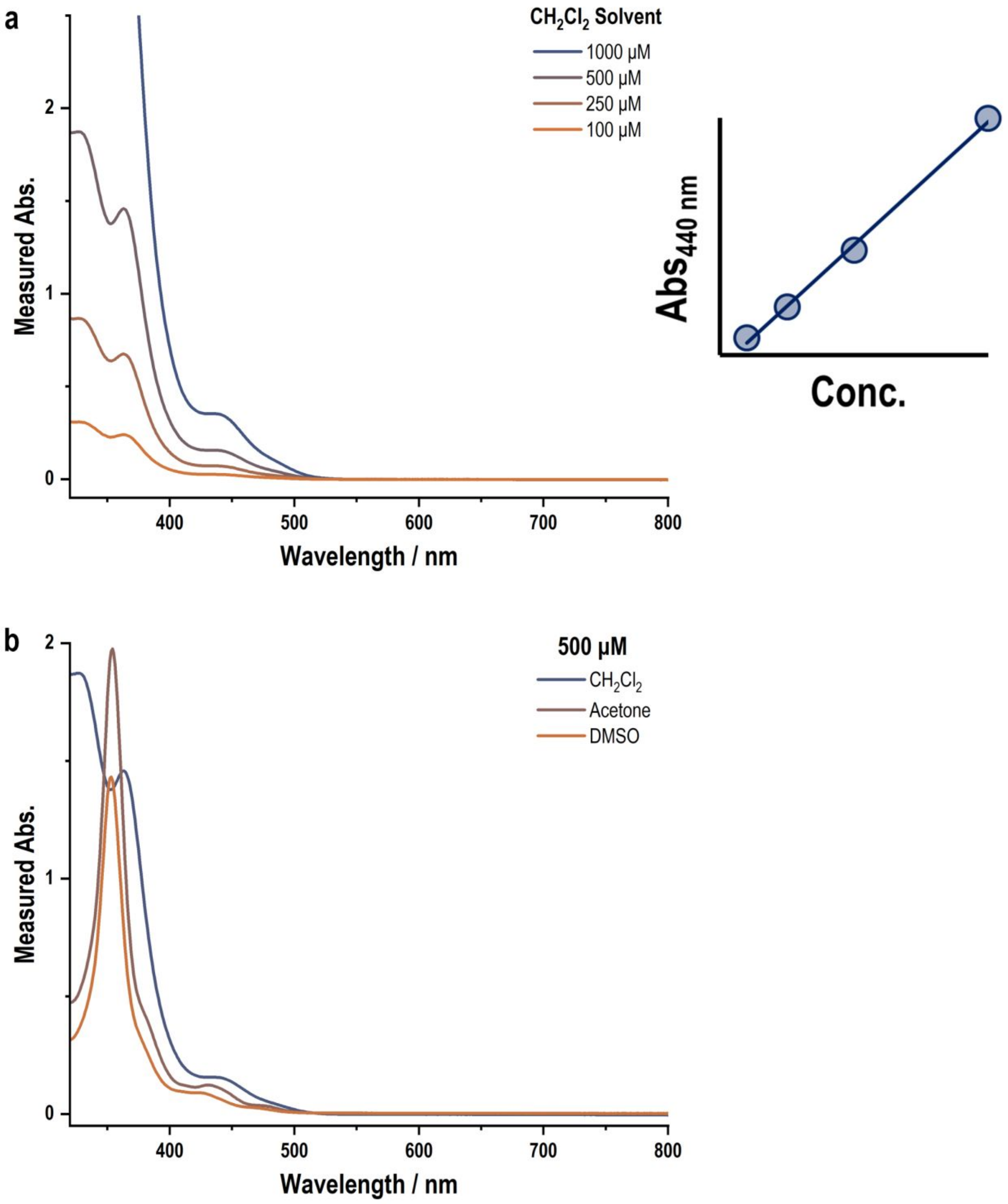

Figure S62: UV-Vis absorption spectra (293 K) for **Rh(*para*-*t*Bu)Cl** in a) CH_2Cl_2 solvent (1000 → 100 μM, CH_2Cl_2) with accompanying Beer-Lambert plot showing linear relationship at 440 nm and b) in MeCN, CH_2Cl_2 and DMSO solvent at 500 μM.

S.6. DOSY Measurement Data

Table S1: Selected DOSY calculated diffusion coefficients in DMSO-d_6 solvent.

Complex	δH	D^{Measured} ($\times 10^{-10}$ m^2s^{-1})	$D^{complex}$ / $D^{\text{Rh}(meta\text{-Me})\text{Cl}}$	**Assignment**
**Rh(*meta*-Me)Cl**	2.36	1.519	1.00	monomer
**Rh(*para*-^{t}Bu)Cl**	1.36	1.491	1.06	monomer
**Rh(*para*-Me)Cl**	2.13	1.112	2.55	dimer

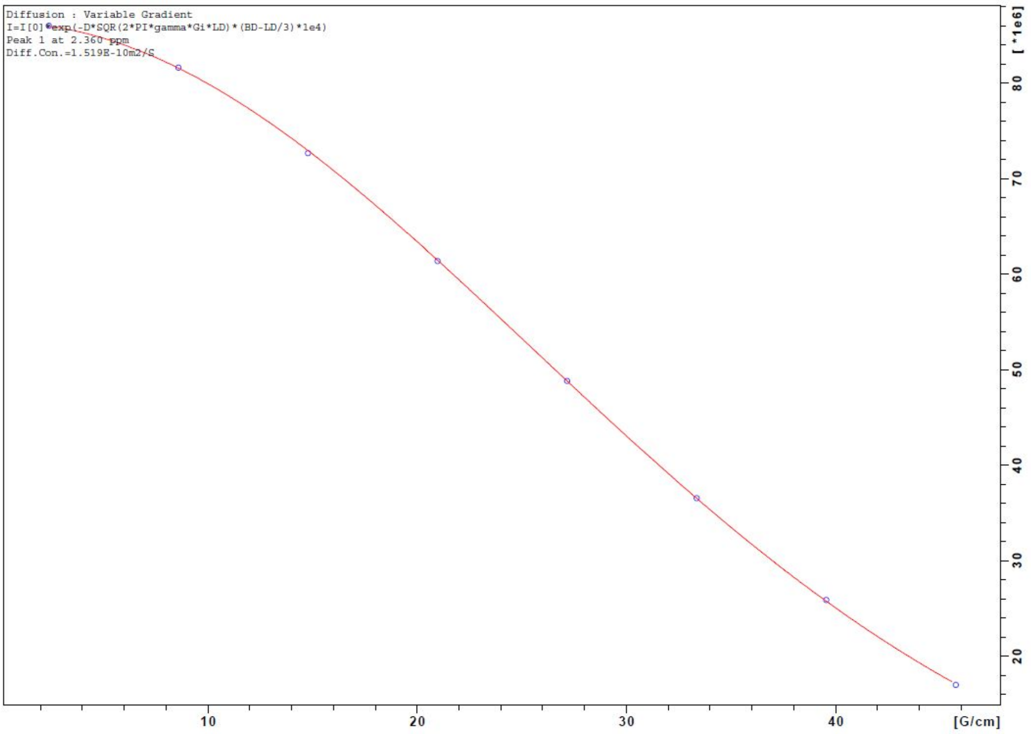

Figure S63: Exponential fit of the amplitude decay for selected proton signals at δ 2.36 ppm for **Rh(*meta*-Me)Cl** in DMSO-d_6.

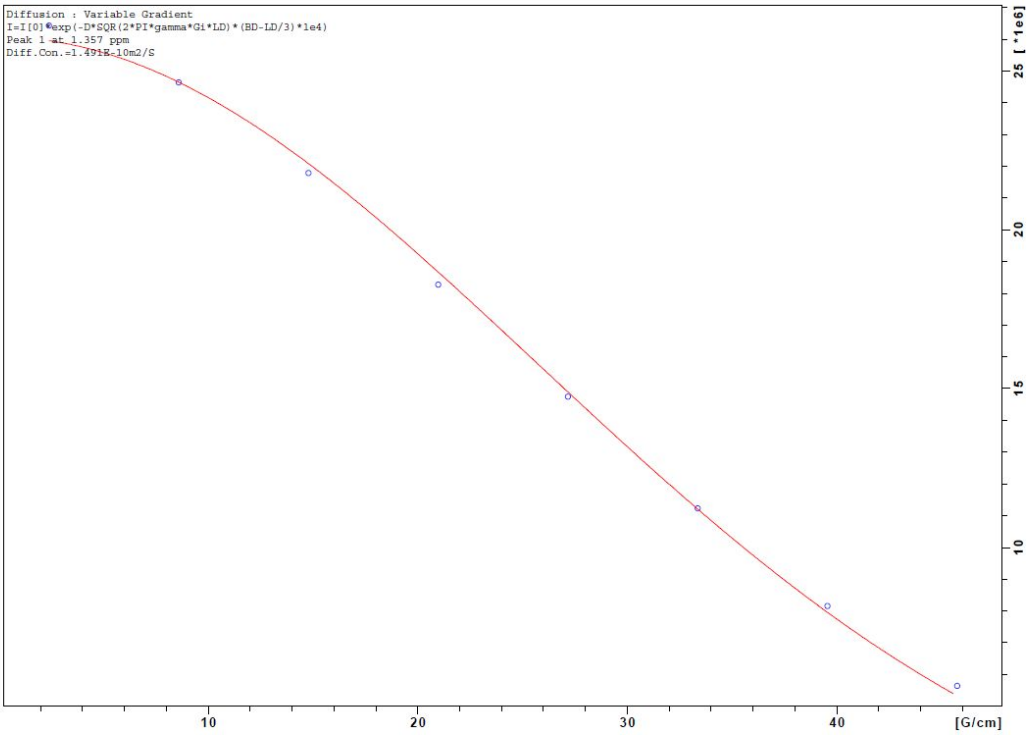

Figure S64: Exponential fit of the amplitude decay for selected proton signals at δ 1.36 ppm for **Rh(*para*-^{t}Bu)Cl** in DMSO-d_6.

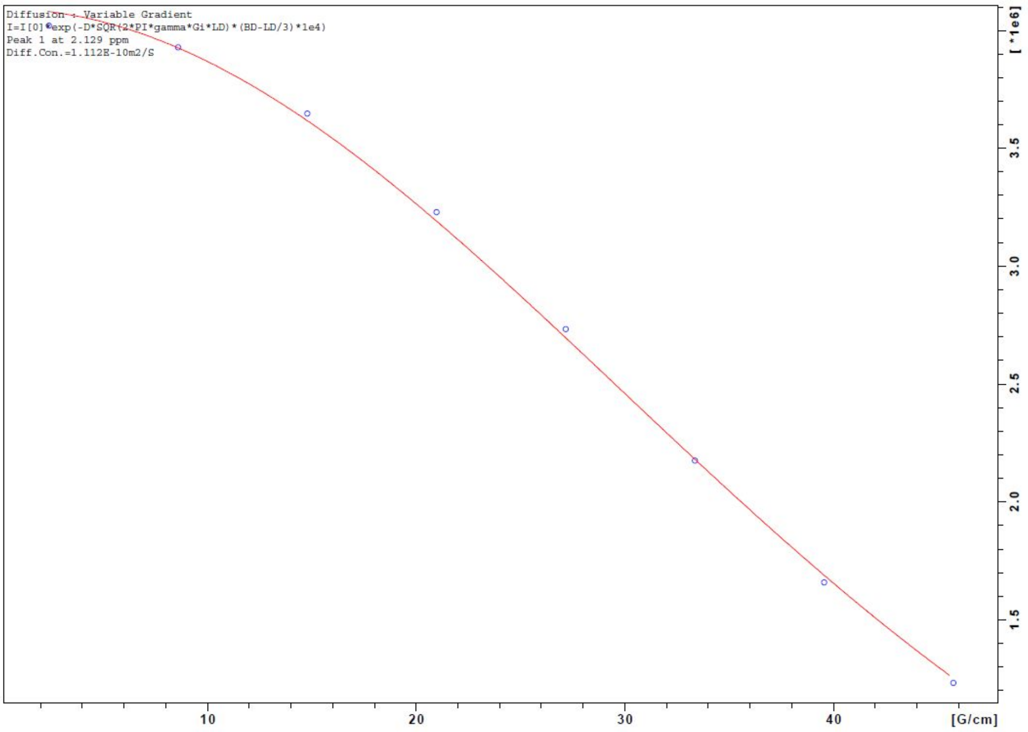

Figure S65: Exponential fit of the amplitude decay for selected proton signals at δ 2.13 ppm for **Rh(*para*-Me)Cl** in DMSO-d_6.

S.7. Crystallographic and Refinement Data

S.7.1. Crystal structure determinations

A suitable crystal was selected, and the crystal was mounted on a MITIGEN holder in perfluoroether oil on a STOE STADIVARI diffractometer. The crystal was kept at 150 K during data collection. Data reductions were performed using the CrysAlisPro program.[S11] Using Olex2,[S12] the structure was solved with the ShelXT[S13] structure solution program using Intrinsic Phasing and refined with the ShelXL[S14] refinement package using Least Squares minimization.

S.7.2. Refinement and Data Tables

Table S2: Selected crystallographic and refinement data.

	**[Rh(*meta*-Me)(1-CN)][PF6]**	**Rh(para-^{t}Bu)Cl**
Identification code	ajb395b_150K	ajb190b_150K
Empirical formula	$C_{47}H_{38}F_6N_4PRh$	$C_{121}H_{140.5}Cl_2N_{13.5}Rh_2$
Formula weight	906.69	2060.68
Temperature/K	150	150
Crystal system	monoclinic	triclinic
Space group	C2/c	P-1
a / Å	27.0306(16)	15.4965(2)
b / Å	19.6729(16)	17.2511(2)
c / Å	16.1010(10)	24.1115(3)
a /°	90	76.9710(10)
β / °	106.759(5)	74.3400(10)
γ / °	90	64.7490(10)
Volume / Å^3	8198.4(10)	5568.26(13)
Z	8	2
ρ_{calc}g/cm^3	1.469	1.229
μ/mm^{-1}	4.304	2.180
F(000)	3696.0	2170.0
Crystal size/mm^3	0.4 × 0.3 × 0.2	0.14 × 0.093 × 0.04
Radiation	Cu Kα (λ = 1.54186)	GaKα (λ = 1.34143)
2Θ range for data collection/°	10.668 to 150.506	5.596 to 111.54
Index ranges	-33 ≤ h ≤ 30, -24 ≤ k ≤ 19, -20 ≤ l ≤ 15	-17 ≤ h ≤ 19, -21 ≤ k ≤ 19, -28 ≤ l ≤ 29
Reflections collected	34867	83953
Independent reflections	8330 [R_{int} = 0.1408, R_{sigma} = 0.1058]	21276 [R_{int} = 0.0363, R_{sigma} = 0.0322]
Data/restraints/ parameters	8330/473/622	21276/212/1258
Goodness-of-fit on F^2	1.067	1.045
Final R indexes [I>=2σ (I)]	R_1 = 0.0833, wR_2 = 0.2063	R_1 = 0.0497, wR_2 = 0.1257
Final R indexes [all data]	R_1 = 0.1586, wR_2 = 0.2740	R_1 = 0.0601, wR_2 = 0.1338
Largest diff. peak/hole / e Å^{-3}	0.88/-0.80	1.07/-1.4
**CCDC Number**	**2483430**	**2483431**

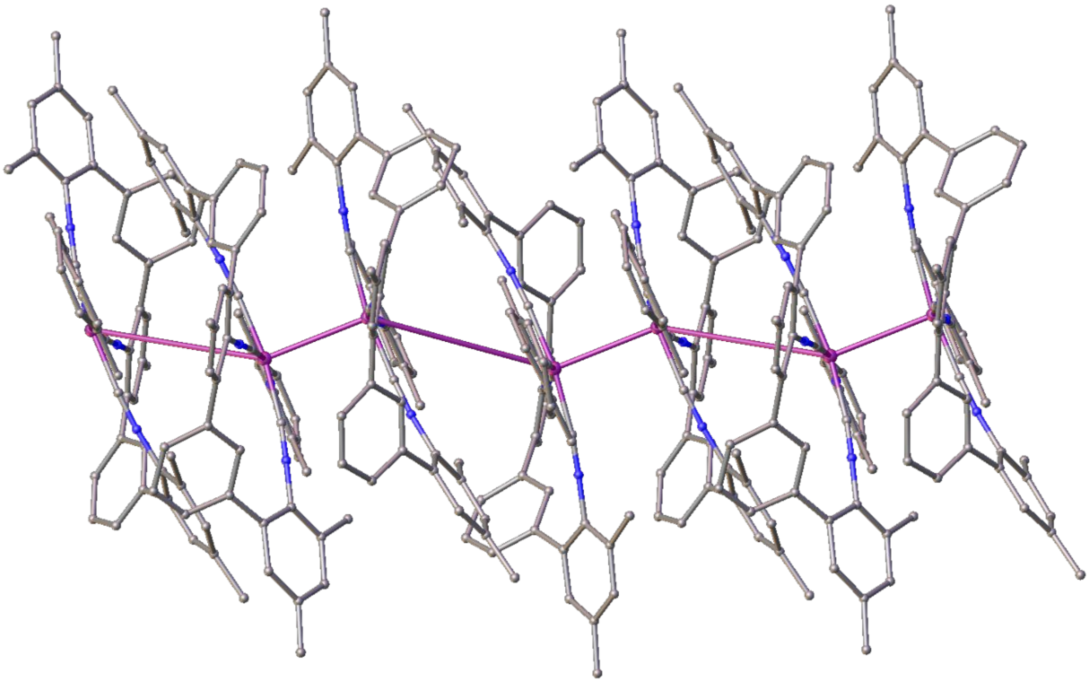

Figure S66: Ball-and-stick expanded solid-state packing structure of **[Rh(*meta*-Me)(1-CN)][PF$_6$].** Hydrogen atoms, solvents and PF_6^- counter anions removed for clarity.

S.8. References

S1: Wu D. H.; Chen A. D.; Johnson C.S.; An Improved Diffusion-Ordered Spectroscopy Experiment Incorporating Bipolar-Gradient Pulses. *J. Magn. Reson. A*, **1995**, 115, 260-264.

S2: Holz M.; Weingärtner H.; Calibration in accurate spin-echo self-diffusion measurements using ^{1}H and less-common nuclei. *J. Magn. Reson.* **1991**, *92*, 115-125.

S3: XWINNMR, Bruker Analytik GmbH, Software Dept., Rheinstetten, Germany.

S4: Smith O.; Popescu M. V.; Hindson M. J.; Paton R. S.; Burton J. W.; Smith M. D.; Control of stereogenic oxygen in a helically chiral oxonium ion. *Nature*, **2023**, *615*, 430.

S5: Wang J.; Yao E.; Chen Z.; Ma Y.; Fluorinated Nickel(II) Phenoxyiminato Catalysts: Exploring the Role of Fluorine Atoms in Controlling Polyethylene Productivities and Microstructures. *Macromolecules*, **2015**, *48*, 5504.

S6: Bilger J. B.; Kerzig C.; Larsen C.B.; Wenger, O.; A Photorobust Mo(0) Complex Mimicking [Os(2,2′-bipyridine)$_3$]$^{2+}$ and Its Application in Red-to-Blue Upconversion. *J. Am. Chem. Soc.*, **2021**, *143*, 1651.

S7: Herr, P.; Kerzig, C.; Larsen, C.B.; Häussinger D.; Wenger O. S.; Manganese(I) complexes with metal-to-ligand charge transfer luminescence and photoreactivity. *Nature Chem.*, **2021**, *13*, 956.

S8: Hu X.; Kang X.; Jian Z.; Suppression of Chain Transfer at High Temperature in Catalytic Olefin Polymerization. *Angew. Chem. Int. Ed.*, **2022**, *61*, e202207363

S9: Reuter, R; Wegner, H. A.; Synthesis and Isomerization Studies of Cyclotrisazobiphenyl. *Chem. Eur. J.,* **2011**, *17*, 2987.

S10: Luo Y.; Cheng S.; Peng Y.; Wang X.; Li J.; Gan C.; Luo S.; Zhu Q.; A New Saddle-Shaped Aza Analog of Tetraphenylene: Atroposelective Synthesis and Application as a Chiral Acylating Reagent. *CCS Chem*, **2022**, *4*, 2897.

S9: Oxford Diffraction (**2018**). CrysAlisPro (Version 1.171.40.37a). Oxford Diffraction Ltd., Yarnton, UK.

S10: Dolomanov, O. V.; Bourhis, L. J.; Gildea, R. J.; Howard, J. A. K; and Puschmann, H.; OLEX2: A Complete Structure Solution, Refinement and Analysis Program. *Al.*, *J. Appl. Cryst.*, **2009**, *49*, 339-341.

S11: Sheldrick, G. M.; SHELXT – Integrated space-group and crystal-structure determination. *Acta Cryst. A,* **2015**, *71*, 3-8.

S12: Sheldrick, G. M.; Crystal structure refinement with SHELXL. *Acta Cryst. C,* **2015**, *71*, 3-8.
